# A new southern Laramidian ankylosaurid, *Akainacephalus johnsoni* gen. et sp. nov., from the upper Campanian Kaiparowits Formation of southern Utah, USA

**DOI:** 10.7717/peerj.5016

**Published:** 2018-07-19

**Authors:** Jelle P. Wiersma, Randall B. Irmis

**Affiliations:** 1 Department of Geosciences, James Cook University, Townsville, QLD, Australia; 2 Natural History Museum of Utah, Salt Lake City, UT, USA; 3 Department of Geology & Geophysics, University of Utah, Salt Lake City, UT, USA

**Keywords:** Paleontology, Taxonomy, Biodiversity

## Abstract

A partial ankylosaurid skeleton from the upper Campanian Kaiparowits Formation of southern Utah is recognized as a new taxon, *Akainacephalus johnsoni*, gen. et sp. nov. The new taxon documents the first record of an associated ankylosaurid skull and postcranial skeleton from the Kaiparowits Formation. Preserved material includes a complete skull, much of the vertebral column, including a complete tail club, a nearly complete synsacrum, several fore- and hind limb elements, and a suite of postcranial osteoderms, making *Akainacephalus johnsoni* the most complete ankylosaurid from the Late Cretaceous of southern Laramidia. Arrangement and morphology of cranial ornamentation in *Akainacephalus johnsoni* is strikingly similar to *Nodocephalosaurus kirtlandensis* and some Asian ankylosaurids (e.g., *Saichania chulsanensis*, *Pinacosaurus grangeri*, and *Minotaurasaurus ramachandrani*); the cranium is densely ornamented with symmetrically arranged and distinctly raised ossified caputegulae which are predominantly distributed across the dorsal and dorsolateral regions of the nasals, frontals, and orbitals. Cranial caputegulae display smooth surface textures with minor pitting and possess a distinct conical to pyramidal morphology which terminates in a sharp apex. Character analysis suggests a close phylogenetic relationship with *N. kirtlandensis*, *M. ramachandrani*, *Tarchia teresae*, and *S. chulsanensis*, rather than with Late Cretaceous northern Laramidian ankylosaurids (e.g., *Euoplocephalus tutus*, *Anodontosaurus lambei*, and *Ankylosaurus magniventris*). These new data are consistent with evidence for distinct northern and southern biogeographic provinces in Laramidia during the late Campanian. The addition of this new ankylosaurid taxon from southern Utah enhances our understanding of ankylosaurid diversity and evolutionary relationships. Potential implications for the geographical distribution of Late Cretaceous ankylosaurid dinosaurs throughout the Western Interior suggest multiple time-transgressive biogeographic dispersal events from Asia into Laramidia.

## Introduction

**Figure 1:**
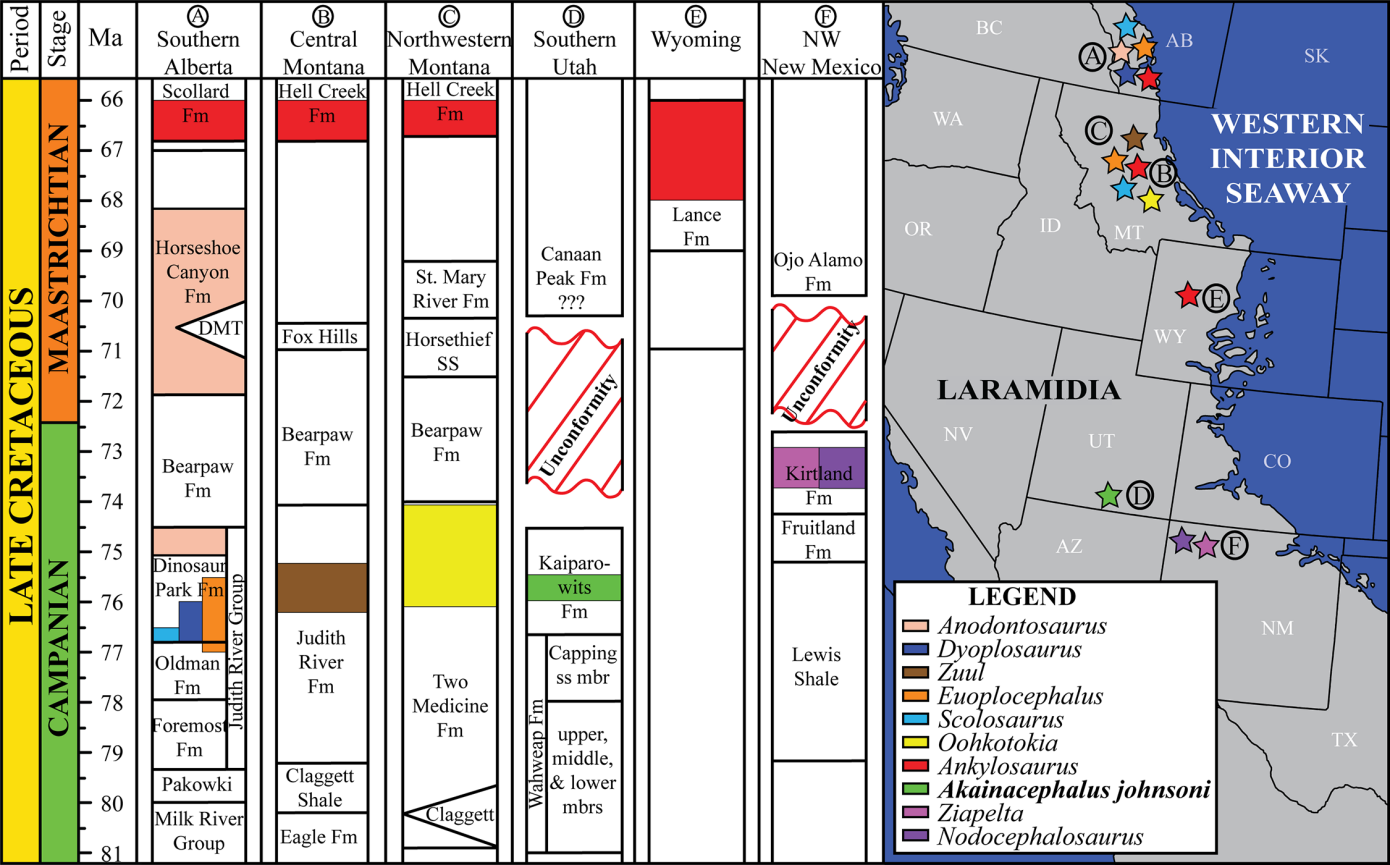
Distribution of Late Cretaceous Laramidian ankylosaurid dinosaurs. Overview of stratigraphic, temporal, and biogeographic distribution of Late Cretaceous (upper Campanian–upper Maastrichtian) ankylosaurid dinosaurs, including *Akainacephalus johnsoni*, across northern Laramidian (Alberta, Montana, Wyoming), and southern Laramidian (Utah, New Mexico) basins. Noticeable is the higher number of taxa and more widespread distribution of ankylosaurids in northern Laramidia. Colored bars within stratigraphic formations represent ankylosaurid taxa and their respective temporal range. Paleogeographic map modified after Sampson et al. (2010). Stratigraphic intervals modified after Arbour & Currie (2013a); Roberts et al. (2013); Johnson, Nichols & Hartman (2002).

The Ankylosauridae is a monophyletic clade of herbivorous, armored ornithischian dinosaurs that are predominantly recorded from the Late Cretaceous (Turonian—late Maastrichtian) of Asia and latest Cretaceous (early Campanian—late Maastrichtian) of western North America (Laramidia) (Lambe, 1902; Brown, 1908; Parks, 1924; Nopcsa, 1928; Sternberg, 1929; Gilmore, 1933; Maryańska, 1977; Sullivan, 1999; Carpenter, 2004; Miles & Miles, 2009; Arbour, Burns & Sissons, 2009; Arbour & Currie, 2013a, 2016; Arbour, Currie & Badamgarav, 2014; Arbour et al., 2014; Penkalski, 2014; Arbour, Zanno & Gates, 2016; Penkalski & Tumanova, 2017; Arbour & Evans, 2017). The majority of Laramidian ankylosaurid specimens are known from northern localities (Fig. 1) and include: *Euoplocephalus tutus*, *Dyoplosaurus acutosquameus*, and *Scolosaurus cutleri* from the Dinosaur Park and Scollard formations of Alberta, Canada; *Anodontosaurus lambei* from the Horseshoe Canyon Formation of Alberta, Canada; *Zuul crurivastator* from the Judith River Formation of Montana; *Oohkotokia horneri* from the Two Medicine Formation of Montana, USA; and *Ankylosaurus magniventris* from the upper Maastrichtian Lance, Hell Creek, and Scollard formations of Wyoming, USA and Alberta, Canada, respectively (Lambe, 1902; Brown, 1908; Parks, 1924; Nopcsa, 1928; Sternberg, 1929; Carpenter, 2004; Arbour, Burns & Sissons, 2009; Arbour & Currie, 2013a; Penkalski, 2014; Arbour & Evans, 2017). Ankylosaurid taxa from southern Laramidia were unknown until the discovery of *Nodocephalosaurus kirtlandensis* from the upper Campanian–Maastrichtian Kirtland Formation of New Mexico (Sullivan, 1999). However, the number of Late Cretaceous ankylosaurid taxa recorded from the Kirtland and Fruitland formations of New Mexico has tripled within the last 15 years, and additional taxa include *Ziapelta sanjuanensis* (Arbour et al., 2014) and *Ahshislepelta minor* (Burns & Sullivan, 2011a), leading to a rapidly increasing taxonomic diversity within southern Laramidian basins during the Late Cretaceous of western North America. In addition, several ankylosaurid specimens (UMNH VP 19472, UMNH VP 19473, UMNH VP 21000, UMNH VP 20202) have been recorded from the upper Campanian Kaiparowits Formation of southern Utah (Loewen et al., 2013a; Wiersma & Irmis, 2013) but UMNH VP 20202 is the first newly-described ankylosaurid taxon from the Late Cretaceous of Utah. Despite these recent discoveries from New Mexico and Utah, Late Cretaceous southern Laramidian ankylosaurid specimens remain rare from upper Campanian terrestrial deposits of the Kaiparowits, Kirtland, and Fruitland formations, and the majority of taxa are represented by a single specimen.

Here, we describe and compare a new genus and species of ankylosaurid dinosaur, *Akainacephalus johnsoni* (UMNH VP 20202), from the upper Campanian Kaiparowits Formation of southern Utah, to other known Late Cretaceous taxa from Asia and western North America. UMNH VP 20202 represents the most complete ankylosaurid specimen from the Kaiparowits Formation and southern Laramidia to date. The specimen consists of a complete skull and mandibles, a nearly complete synsacrum, various cervical, dorsal, and caudal vertebrae, including the tail club handle and knob, a large number of fore- and hindlimb elements, two cervical half rings, and a suite of postcranial osteoderms. The distinct combination and arrangement of conical and pyramid-shaped caputegulae, the massive and backswept postorbital horns, and the ventrally descending, triangular squamosal horns in *Akainacephalus johnsoni* make this specimen taxonomically unique among other Late Cretaceous ankylosaurid dinosaurs from Asia and western North America. The overall morphology of *A. johnsoni* suggests a close phylogenetic relationship with *Nodocephalosaurus kirtlandensis* and Late Cretaceous Asian ankylosaurids.

### Geologic setting

**Figure 2:**
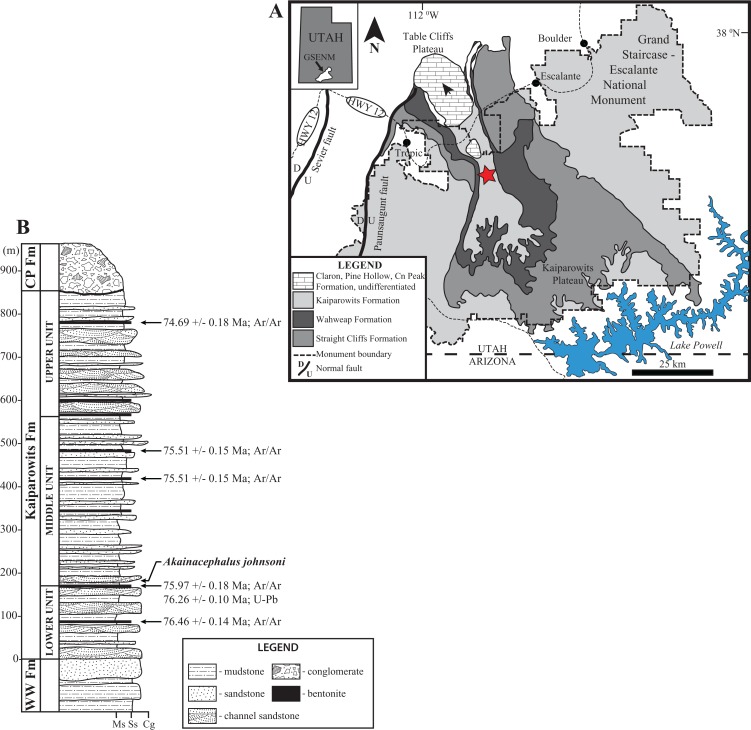
Location and stratigraphy of the Kaiparowits Formation. Map of Grand Staircase-Escalante National Monument (GSENM), southern Utah (A), and generalized stratigraphic section of the upper Campanian Kaiprowits Formation (B). The approximate stratigraphic position of *Akainacephalus johnsoni* is located near the base of the middle unit within the Kaiparowits Formation. The map highlights the GSENM boundary (dashed line), showing the geological distribution and outcrops of the Cretaceous Kaiparowits, Wahweap, and Straight Cliffs formations. The red star indicates the Horse Mountain area, from which *Akainacephalus johnsoni* was recorded. Map and stratigraphic column modified from Roberts (2005). Radioisotopic dates used from Roberts, Deino & Chan (2005) and Roberts et al. (2013), respectively.

*Akainacephalus johnsoni* was discovered in the Kaiparowits Formation; an ∼860-m-thick terrigenous siliciclastic stratigraphic succession (Fig. 2A) that crops out in the Kaiparowits Plateau within the Grand Staircase-Escalante National Monument (GSENM) in southern Utah, USA (Fig. 2B) and contributes to a substantial part of the 2 km thick Late Cretaceous stratigraphic sequence within the Kaiparowits Basin (Roberts, 2007). From a paleontological perspective, the Kaiparowits Formation preserves a unique record of Late Cretaceous terrestrial vertebrate ecosystems in the Western Interior of North America. This thick succession of strata was deposited at an unusually rapid rate within a time frame of ∼2 million years, making it one of the most rapidly deposited terrestrial formations in the world (Roberts, 2005). Recently recalibrated radioisotopic dates for the Kaiparowits Formation indicate a late Campanian age range of 76.46 ± 0.14 Ma for the upper portion of the lower unit to 74.69 ± 0.18 Ma for the uppermost portion of the upper unit of the Kaiparowits Formation (Fig. 2A) (Roberts, 2005; Roberts, Deino & Chan, 2005; Roberts et al., 2013), making it contemporaneous with dinosaur-bearing strata from the Dinosaur Park Formation, Alberta, Judith River and Two Medicine formations, Montana, Fruitland and Kirtland formations, New Mexico, and the Aguja Formation, Texas (Fig. 1).

Topographically, the strata within the Kaiparowits Formation are characterized by their badland-forming bluish-gray siltstone and mudstone and grayish sandstone outcrops, overlying the more cliff-forming predominantly yellow-brownish fluvial channel deposits that form the lower-middle Campanian Wahweap and upper Turonian–Santonian Straight Cliffs formations (Eaton et al., 2001; Roberts, 2005, 2007; Roberts, Deino & Chan, 2005; Jinnah et al., 2009; Jinnah, 2013; Roberts et al., 2013). *Akainacephalus johnsoni* (UMNH VP 20202) was discovered at the Horse Mountain Gryposaur (HMG) Quarry, UMNH VP Locality 1109, a multitaxic and multidominant bonebed, deposited in a fine- to medium-grained sandstone crevasse splay, intercalated within a silty mudstone. The quarry, and therefore UMNH VP 20202, is located within the lower section of the informal middle unit of the Kaiparowits Formation, approximately 190 m above the basal contact of the formation in the Horse Mountain region (Roberts et al., 2013). This bonebed has also produced a nearly complete skull and postcranial skeleton of the hadrosaurid ornithischian dinosaur *Gryposaurus* (UMNH VP 20181 in Gates et al., 2013), the type specimen of the baenid turtle *Arvinachelys goldeni* (Lively, 2015), a nearly complete articulated skull and postcranial skeleton of a new taxon of small alligatoroid (Irmis et al., 2013), and a poorly preserved partial skull of a small theropod dinosaur (Zanno et al., 2013).

## Materials and Methods

UMNH VP 20202 was prepared using small airscribes, dental tools, brushes, and a microscope. Cleaning was performed exclusively by using water and paper towels. Polyvinal acetate (Vinac™) dissolved in acetone was applied as a consolidant, and cyanoacrylate and two-part epoxy were used as adhesives. Two-part epoxy putty was applied to selected areas to complement stabilization or fill in large fractures but was not applied for reconstructive purposes. After preparation, the skull was subjected to computed tomography (CT) scanning in order to reveal the internal anatomy, particularly in the endocranial cavity and nasal regions. CT scanning was performed at the University of Texas High Resolution X-ray Computed Tomography Facility in Austin, TX, USA. UMNH VP 20202 was scanned in January 2014, using a NSI scanner equipped with a Titan GE source set at 450 kV and 3.0 mA and voxel size of 0.1692 mm. Anatomical comparisons were made with closely related taxa; comparisons to other taxa in the description are referenced, following standard modern paleontological descriptive practice, citing specimen numbers when material was observed personally, and citing published references when comparisons are made with the literature. A complete list of all examined taxa and specimen numbers are summarized in Table S1 (Supplemental Material S1). Measurements performed on UMNH VP 20202 are summarized in Supplemental Material S4.

UMNH VP 20202 is permanently reposited in the collections of the Natural History Museum of Utah, Salt Lake City, UT, USA. Detailed locality information is on file and available to qualified researchers as per museum policy. All specimens were collected under permits obtained from the United States Department of the Interior Bureau of Land Management (BLM) in the BLM-administered GSENM.

### Nomenclatural acts

The electronic version of this article in portable document format will represent a published work according to the International Commission on Zoological Nomenclature (ICZN), and hence the new names contained in the electronic version are effectively published under that Code from the electronic edition alone. This published work and the nomenclatural acts it contains have been registered in ZooBank, the online registration system for the ICZN. The ZooBank Life Science Identifiers (LSIDs) can be resolved and the associated information viewed through any standard web browser by appending the LSID to the prefix http://zoobank.org/. The LSID for this publication is: [urn:lsid:zoobank.org:pub:42CB4F68-9E0D-42BB-943D-8A66E3D9DA12]. The online version of this work is archived and available from the following digital repositories: PeerJ, PubMed Central, and CLOCKSS.

## Results

### Systematic paleontology

Dinosauria Owen, 1842 *sensu* Padian and May, 1993
Ornithischia Seeley, 1887 *sensu* Padian and May, 1993
Thyreophora Nopcsa, 1915 *sensu* Sereno, 1986
Ankylosauria Osborn, 1923 *sensu* Carpenter, 1997
Ankylosauridae Brown, 1908 *sensu* Sereno, 1998
Ankylosaurinae Brown, 1908 *sensu* Sereno, 1986
Ankylosaurini Arbour and Currie, 2016
*Akainacephalus*, gen. nov.
*Akainacephalus johnsoni*, sp. nov.

### Holotype

UMNH VP 20202, a partial skeleton comprising a complete skull, both mandibles, predentary, four dorsal, four dorsosacral, three sacral, one caudosacral, and eight caudal vertebrae, dorsal ribs, a complete tail club, both scapulae, left coracoid, right humerus, right ulna, partial left ilium, left femur, left tibia, left fibula, phalanx, two partial cervical osteoderm half rings, and 17 dorsal and lateral osteoderms of various sizes and morphologies.

### Type locality

UMNH VP Locality 1109 (“HMG Quarry”), Horse Mountain area, GSENM, Kane County, southern Utah, USA.

### Type stratigraphic horizon and age

UMNH VP Locality 1109 is a multitaxic bonebed deposited in a crevasse splay sandstone within the lower portion of the middle unit of the upper Campanian Kaiparowits Formation (Fig. 2A). The stratigraphic position of this site is approximately 190 m from the base of the formation (Roberts et al., 2013: fig. 6.3) and within approximately one meter stratigraphic proximity of the recently dated bentonite ash bed KP-07, which has produced a U-Pb zircon age of 76.26 ± 0.10 Ma (Roberts et al., 2013), providing a precise age constraint for *Akainacephalus johnsoni*.

### Etymology

The genus name is derived from the Greek *akaina*, meaning “thorn” or “spine,” referring to the thorn-like cranial caputegulae of the holotype; and “*cephalus*,” the Greek meaning for head. The specific epithet honors Randy Johnson, volunteer preparator at the Natural History Museum of Utah, who skillfully prepared the skull and lower jaws of UMNH VP 20202.

### Diagnosis

*Akainacephalus johnsoni* possesses the following autapomorphies: massive supraorbital bosses in lateral view, forming a tall backswept flange extending laterally over the orbits, and enveloping the anterodorsal and posterior margins of the orbit; nearly vertical projecting triangular quadratojugal horns; frontal possesses a large, flat, and centrally positioned hexagonal-shaped caputegulum; a combination of tightly spaced, symmetrically positioned pyramidal and conical-shaped caputegulae across the frontonasal region; a distinct midline row of conical-shaped caputegulae across the nasal region, symmetrically separating caputegulae situated dorsolaterally; basioccipital foramen anterior and dorsally to the occipital condyle. *A. johnsoni* also possesses a unique combination of character states: shares with *Nodocephalosaurus kirtlandensis* the presence of a large, laterally oriented supranarial osteoderm forming the postmaxillary/lacrimal ridge dorsal to the external nares; differs from *Tsagantegia longicranialis*, *Talarurus plicatospineus*, *Pinacosaurus grangeri*, all northern Laramidian taxa and *Ziapelta sanjuanensis* but shares with *Nodocephalosaurus kirtlandensis*, *Minotaurasaurus ramachandrani*, *Saichania chulsanensis*, and *Tarchia kielanae* the presence of well-pronounced cranial ornamentation located along the nasal and frontal regions of the skull that are characterized by a dense array of well-defined caputegulae with a distinct conical (*N. kirtlandensis*) and pyramidal (*M. ramachandrani*, *S. chulsanensis*, *T. kilanae*) morphology; shares with *Euoplocephalus* and *Zuul crurivastator* a globular surface texture on the tail club knob, which differs from the smoother texture in *Ankylosaurus magniventris*; differs from ZPAL MgD I/113, cf. *Pinacosaurus*, *Saichania chulsanensis*, and *Dyoplosaurus acutosquameus*, but similar to *Anodontosaurus lambei*, *Euoplocephalus tutus, Zuul crurivastator,* and *Ankylosaurus magniventris* in having a wider than long tail club knob ratio; and shares with ZPAL MgD I/113, cf. *Pinacosaurus*, *D. acutosquameus*, and *Zuul crurivastator* triangular osteoderms along the lateral surfaces on the proximal portion of the tail.

## Osteological and Comparative Description

### Preservation

UMNH VP 20202 is well-preserved overall and comprises ∼45% of the skeleton, including the armor. Among the postcrania, only a few elements are poorly preserved. The ulna and fibula are all heavily postdepositionally fractured, missing large portions of their proximal and/or distal ends. The skull is complete, including both mandibles and the predentary. Upon discovery, the skull was positioned vertically in the sediment, the snout facing downwards. Fractures along the posterior margins of the postorbital bosses and the anteroventral margin of the orbits suggests that the skull underwent hinge-like anteroposterior deformation. This resulted in anteroventral rotation of the posterior part of the skull, including the basicranial elements (pterygoids, quadrates, basisphenoid, basioccipital, supraoccipital, exoccipitals), which kept interelemental breakage to a minimum. Areas most prominently affected by the deformation include the premaxilla-maxillary and orbital regions, and the choanae. No teeth are preserved in the maxillae. The orbits are dorsoventrally oblong as a result of significant anteroposterior compression. A broken, partial right squamosal horn is preserved, and the left squamosal horn is completely broken away, making assessment of their original morphology impossible. Both mandibles are preserved but are incomplete due to postdepositional breakage and predepositional weathering along most of their surfaces. The anterior and dorsal surfaces of the dentaries are broken and preserve no teeth. Medially, the dentary, splenial, surangular, and prearticular are broken and show signs of predepositional weathering. The articular is broken away from the right mandible. The predentary is nearly complete but is missing the left lateral portion that articulates with the left dentary.

### Cranium

**Figure 3:**
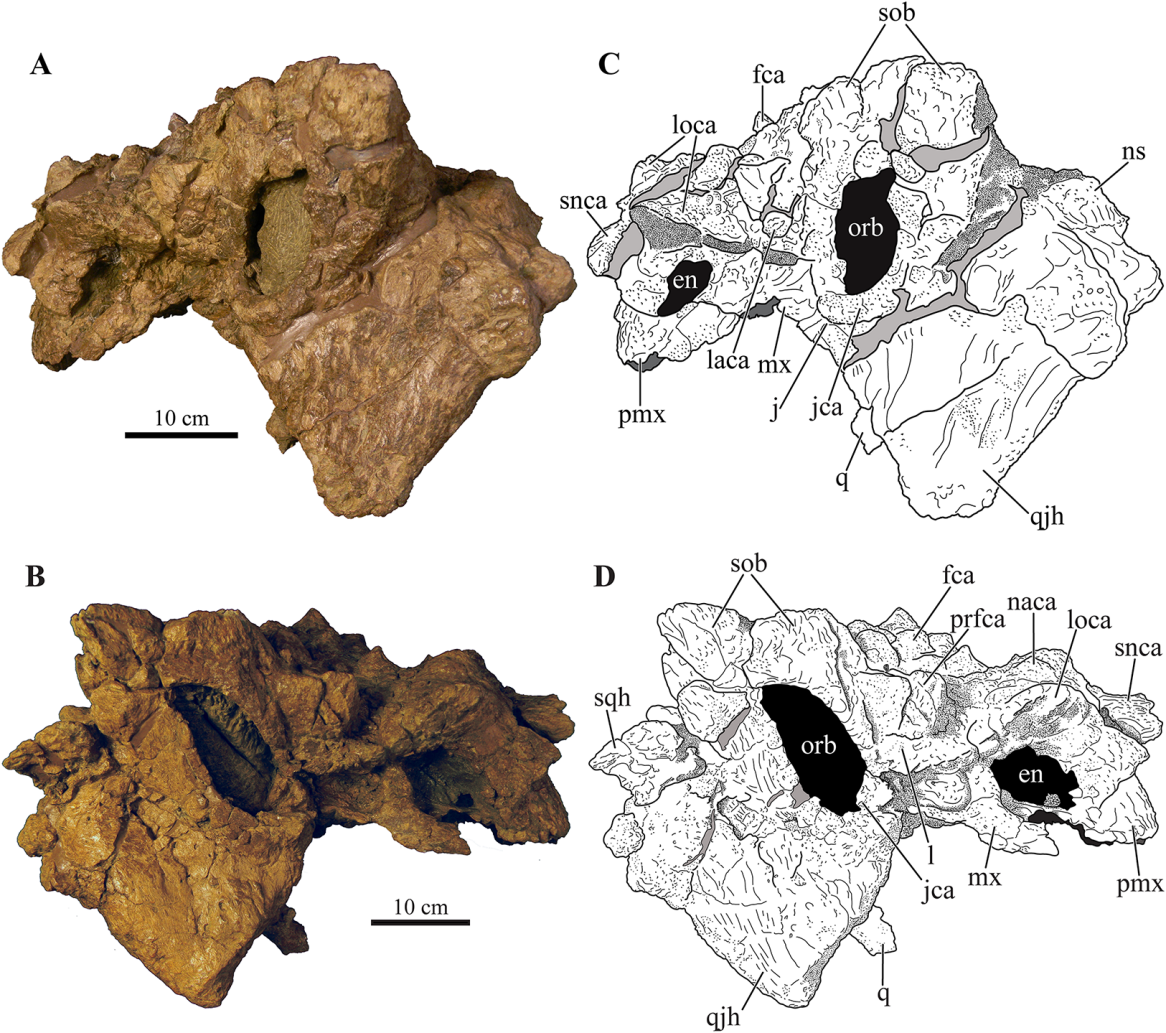
Skull of *Akainacephalus johnsoni* (UMNH VP 20202). Photographs of the skull of *Akainacephalus johnsoni* in (A), left lateral; and (B), right lateral views. Line drawings in (C), left lateral; and (D), right lateral views highlight major anatomical features. Study sites: en, external naris; fca, frontal caputegulum; j, jugal; jca, jugal caputegulum; l, lacrimal; laca, lacrimal caputegulum; loca, loreal caputegulum; mx, maxilla; n, nasal; naca, nasal caputegulae; ns, nuchal shelf; orb, orbit; pmx, premaxilla; prfca, prefrontal caputegulum; snca, supranarial caputegulum; sob, supraorbital boss; q, quadrate; qjh, quadratojugal horn; sqh, squamosal horn.

**Figure 4:**
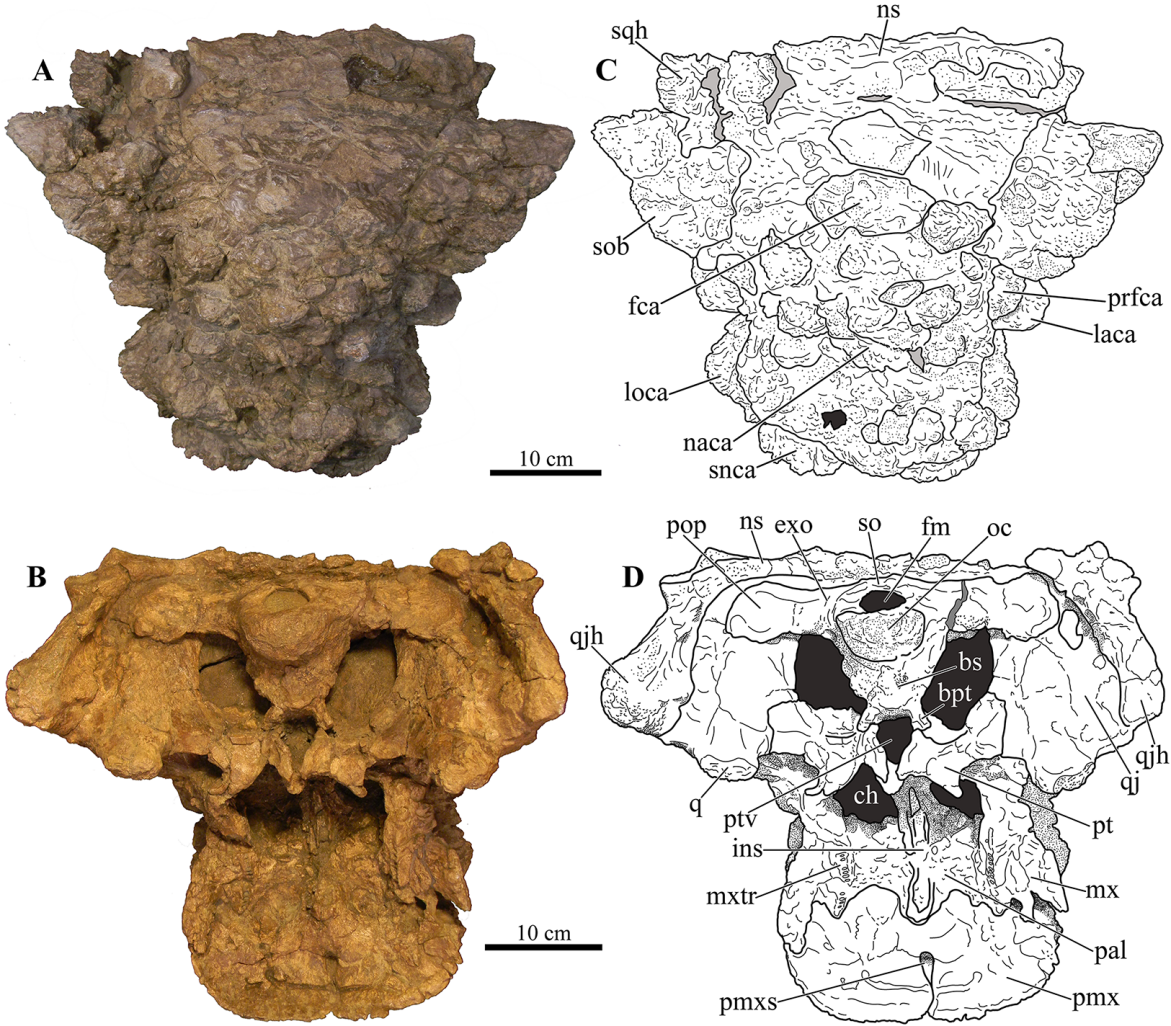
Skull of *Akainacephalus johnsoni* (UMNH VP 20202). Photographs of the skull of *Akainacephalus johnsoni* in (A), dorsal; and (B), ventral views. Line drawings in (C), dorsal; and (D), ventral views highlight major anatomical features. Study sites: bpt, basipterygoid; bs, basisphenoid; ch, choana; exo, exoccipital; fm, foramen magnum; fca, frontal caputegulum; ins, internarial septum; laca, lacrimal caputegulum; loca, loreal caputegulum; mx, maxilla; mxtr, maxillary tooth row; naca, nasal caputegulum; ns, nuchal shelf; oc, occipital condyle; pal, palatine; prfca, prefrontal caputegulum; pmx, premaxilla; pmxs, interpremaxillry suture with oblong depression; pop, paroccipital process; ptv, pterygoid vacuity; q, quadrate; qj, quadratojugal; qjh, quadratojuga horn; so, supra occipital; snca, supranarial caputegulum; sob, supraorbital boss; sqh, squamosal horn.

Perhaps the most striking feature of *Akainacephalus johnsoni* is the exterior surface of the skull (Fig. 3), which contains a unique suite of cranial ornamentation comprising several symmetrical rows of small pyramidal and conical caputegulae along the dorsolateral surface of the skull, including a distinct midline row of tall, well-pronounced caputegulae (Figs. 3, 4A and 4C). Ornamentation across the midline of the frontal region is limited to a single, transversely long, hexagonal-shaped caputegulum of low relief and contains minor rugose surface texture (Fig. 4A). The nuchal shelf is an anteroposteriorly short structure that is positioned transversely across the dorsal surface of the skull. It contacts the posteriormost margin of the frontals. The anterior portion of the nuchal area is devoid of caputegulae medially, and instead forms a thickened ossified region containing minor rugose surface textures; however, several prominent caputegulae are present on the left and right sides of the nuchal shelf that appear to fuse with the dorsal margin of the quadratojugal horn. In dorsal view, the nuchal shelf completely obscures the basioccipital (Figs. 4A and 4C). Overall, the ventral region of *Akainacephalus johnsoni* (Figs. 4B and 4D) is relatively complete and most breakage is caused by anteroposterior displacement along the transverse plane. Most of the ventral parts of the premaxillae are preserved but the premaxilla-vomer contact is broken away, as is the anterior-most part of the vomer. Posteriorly, the contacts between the vomer-secondary palate and vomer-pterygoid are largely preserved but are slightly offset due to transverse displacement. The secondary palate is nearly complete. Most of the endocranial elements are preserved including the supraoccipital, basioccipital, basisphenoid, exoccipitals, and paroccipital processes, but experienced significant predepositional weathering, removing most of the surface texture details and external margins of each element. In overall proportions, the mandibles are dorsoventrally tall compared to most other ankylosaurid mandibles (e.g., *Euoplocephalus tutus* [UALVP 31, AMNH FARB 5403, AMNH FARB 5405], *Ankylosaurus magniventris* [AMNH FARB 5214, CMN 8880], *M. ramachandrani* [INBR 21004], and *P. grangeri* [MPC 100/1014]), but are similar to *Saichania chulsanensis* (MPC 100/151). No teeth are preserved with the mandibles, but the alveolar cavities in the left dentary suggest the presence of 16–18 teeth during life. Both mandibles are heavily weathered and eroded, predominantly along the posterior and anterior margins, removing most of the articular surfaces that contact the quadrates as well as the mandibular symphyseal surfaces that articulate with the predentary.

### Major cranial fenestrae, foramina, and fossae

#### External nares

The external nares are oriented laterally, similar to *Nodocephalosaurus kirtlandensis* (SMP VP-900) and *Ankylosaurus magniventris* (Carpenter, 2004), and are fully obscured in anterior view, a condition that contrasts with many other Asian and Laramidian ankylosaurids in which the external nares have an anterior or anterolateral orientation, such as *P. mephistocephalus* (Godefroit et al., 1999), *M. ramachandrani* (INBR 21004), *Tsagantegia longicranialis* (Tumanova, 1993), *Euoplocephalus tutus* (AMNH FARB 5405, TMP 1991.127.1, UALVP 31), and *Anodontosaurus lambei* (CMN 8530, TMP 1997.132.1). Compared to the large loreal caputegulum, the external nares are relatively small, tear-shaped openings (Fig. 3A). A small bony fragment positioned on the posterior margin of the left nares might suggest the presence of internarial apertures as observed in *P. grangeri* (Maryańska, 1977; Hill, Witmer & Norell, 2003) and *M. ramachandrani* (Miles & Miles, 2009). However, these taxa possess very prominent and large external nares, containing at least three internarial apertures per naris, compared to the small external nares observed in *Akainacephalus johnsoni*. The dorsal and anterior margins of the external nares are bound by the supranarial caputegulum, whereas the posterior end of the premaxillary tomium defines the ventral boundary. Internally, the nares are separated by the internasal septum, which consists of a paired element positioned along the medial margins of the nasals.

#### Internal nares

The anteromedial surfaces of the maxillae and lateral surfaces of the internasal septum form the boundaries of the choanae (Figs. 4B and 4D). The anterior margins of the secondary palate and therefore the posterior boundary of the choanae are not well preserved, likely due to postdepositional crushing of the skull. This results in choanae that are anteroposteriorly short and mediolaterally broad, resembling an ellipsoid.

#### Orbit

The orbits are heavily distorted by anteroposterior deformation, resulting in an almond-shaped morphology (Fig. 3). Compressive deformation along the anteroposterior short axis displaced both the jugals slightly over the lacrimals by approximately 0.5 cm, shortening the orbits (Fig. 3). Both jugals are positioned on the ventral-most border of the orbit and possess a semilunate, dorsally concave morphology, forming a small cradle with a laterally extending, shallow shelf. The orbits are anterolaterally oriented.

#### Foramen magnum

**Figure 5:**
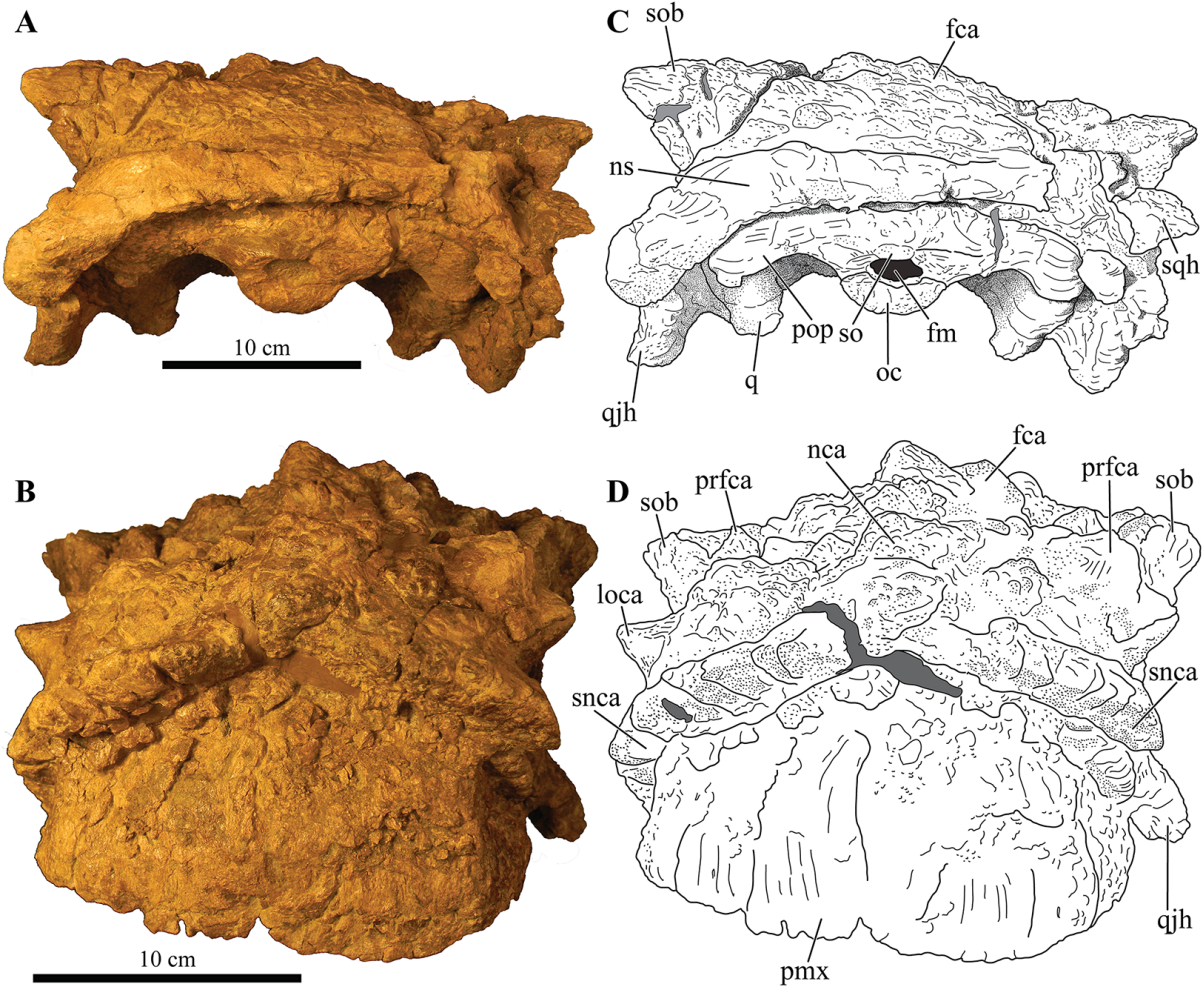
Skull of *Akainacephalus johnsoni* (UMNH VP 20202). Photographs of the skull of *Akainacephalus johnsoni* in (A), posterior; and (B), anterior views. Line drawings in (C), posterior; and (D), anterior views highlight major anatomical features. Study sites: fm, foramen magnum; fca, frontal caputegulae; loca, loreal caputegulum; naca; nasal caputegulae; ns, nuchal shelf; oc, occipital condyle; pmx, premaxilla; pop, paroccipital process; prfca, prefrontal caputegulum; q, quadrate; qjh, quadratojugal horn; snca, supranarial caputegulum; so, supraoccipital; sob, supraorbital boss; sqh, squamosal horn.

The foramen magnum (Fig. 5A) is semicircular with a mediolaterally oriented long axis and has a posteroventral orientation. It is comprised of the concave dorsal margin of the basioccipital, the medial margins of the exoccipitals, and the ventral margin of the supraoccipital. The dorsal margin of the occipital condyle (= basioccipital) extends slightly posterior to the foramen magnum. The foramen magnum is dorsally bound by two broken and isolated surfaces that form the remnants of a prominent pair of caudoventromedially oriented transverse nuchal crests (= oval tuberosities, (Nopcsa, 1928) and proatlas facet, (Carpenter, 2001)). Because both broken surfaces represent individual elements, it appears that an incisive notch (*sensu* Vickaryous, 2001) was present during life.

#### Cranial foramina

**Figure 6:**
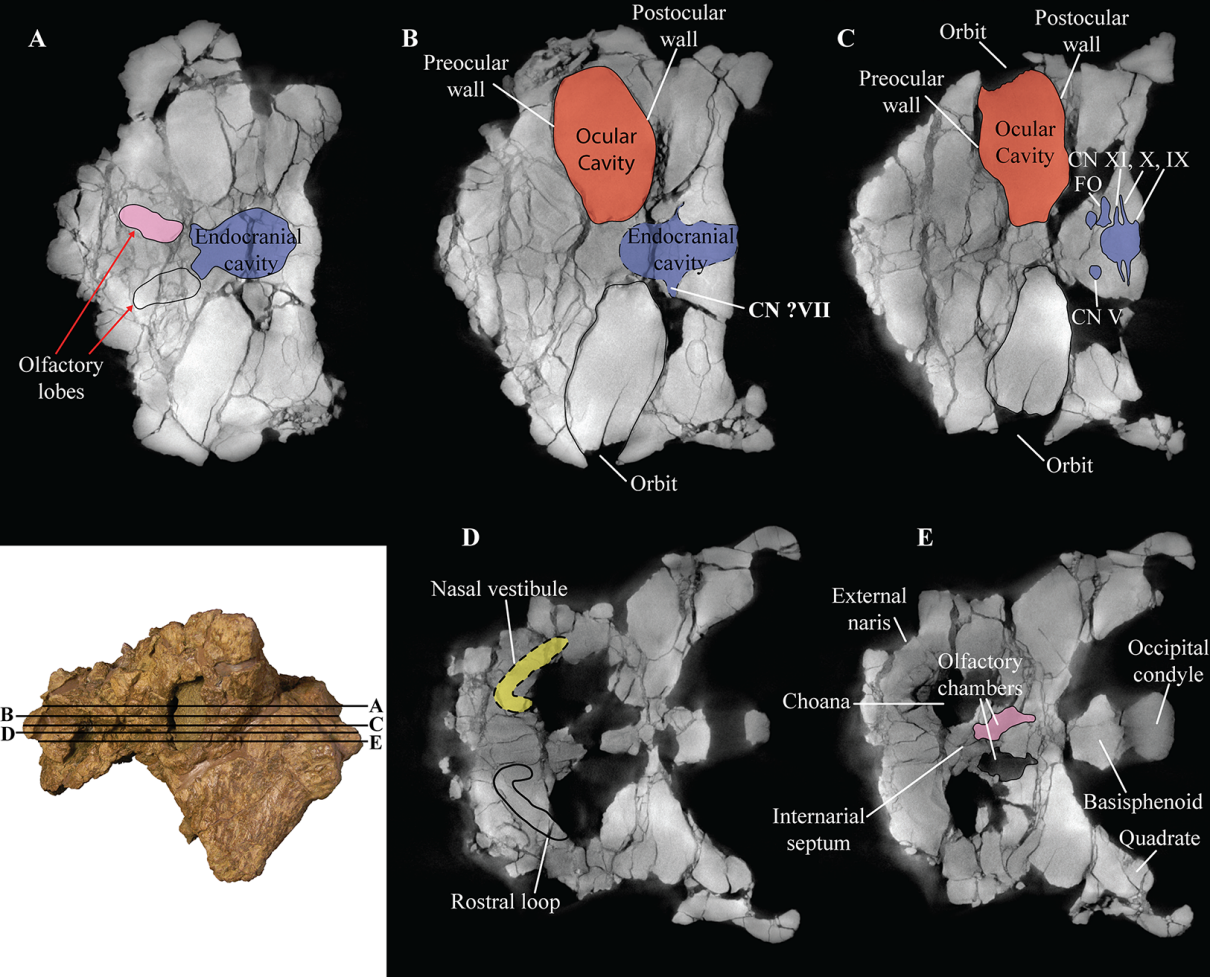
Computed tomography (CT) scans of *Akainacephalus johnsoni* (UMNH VP 20202). Transverse plane CT visualization of the skull of *Akainacephalus johnsoni* revealing important anatomical features, otherwise obscured by bone and/or matrix. Anatomical features in skull of *A. johnsoni* with horizontal lines indicating localities for the CT slices; (A), oblong-shaped olfactory lobes and outline of the endocranial cavity which provides space for the brain, including its olfactory lobes; (B), and (C), ocular cavities and their surrounding pre- and postocular walls. Presence of cranial nerves CN V, ?VII, IX, X, XI and the fenestra ovalis (FO) along the lateral surfaces of the endocranial cavity; (D), anatomical outline of a small portion of the complex nasal air passage system showing the rostral loop of the nasal vestibule; (E) outlines of the olfactory chambers which house the olfactory lobes. Images not to scale.

The endocranial cavity, including a total of five cranial fenestrae have been identified with the aid of CT scanning (Figs. 6A–6E). These foramina form the accommodation space for various cranial nerves (CN). The anterior-most cranial foramen forms the housing for trigeminal nerve (CN V) and is located on the ventral margin between the laterosphenoid and prootic bones (Vickaryous, Maryańska & Weishampel, 2004). In *Akainacephalus johnsoni*, the opening for the trigeminal nerve is circular in dorsal view (Fig. 6C). Situated posterior to CN V, and positioned between the prootic and opisthotic is the fenestra ovalis, which is a laterally oblong opening in dorsal view that exits directly anterior to the exoccipitals (Fig. 6C). Posterior to the fenestra ovalis are three foramina for the glossopharyngeal (CN IX), vagus (CN X), and accessory nerves (CN XI), respectively (Fig. 6C). The morphology of the openings are very narrow and needle-like. The foramen for CN IX forms a triple junction between the anteroventral portion of the exoccipital, posteroventral portion of the opisthotic, and dorsal portion of the basioccipital. CN X and CN XI are positioned between the ventral portion of the opisthotic and the dorsal portion of the basioccipital. In *Euoplocephalus tutus*, these foramina are positioned posteroventrally to the fenestra ovalis (Vickaryous, Maryańska & Weishampel, 2004). However, in *Akainacephalus johnsoni*, these foramina appear directly posterior to the fenestra ovalis, similar to *Saichania chulsanensis* (Maryańska, 1977).

### Dermatocranial horns, bosses, and caputegulae

**Figure 7:**
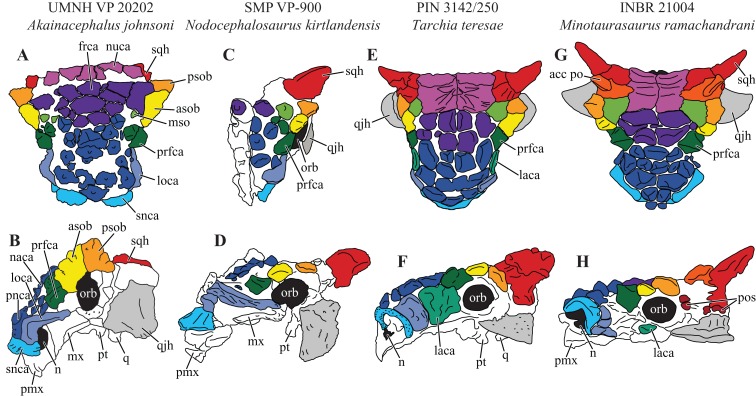
Variation in cranial ornamentation in selected Laramidian and Asian taxa, including *Akainacephalus johnsoni*. Comparative line drawings highlighting major areas of cranial ornamentation in *Akainacephalus johnsoni* and closely related Laramidian and Asian taxa. *Akainacephalus johnsoni* (UMNH VP 20202) in (A), dorsal; (B), left lateral view compared to *Nodocephalosaurus kirtlandensis* (SMP VP-900) in (C), dorsal; and (D) left lateral view; *Tarchia teresae* (PIN 3142/250) in (E) dorsal; (F), left lateral view and *Minotaurasaurus ramachandrani* (INBR 21004) in (G), dorsal; and (H), left lateral view. Study sites: acc po, accessory postorbital ossification; asob, anterior supraorbital boss; frca, frontal caputegulum; laca, lacrimal caputegulum; loca, loreal caputegulum; mso, medial supraorbital; mx, maxilla; n, external naris; naca, nasal caputegulae; nuca, nuchal caputegulae; orb, orbital; pmx, premxilla; pnca, postnarial caputegulum; pos postocular ossicles; prfca, prefrontal caputegulum; psob, posterior supraorbital boss; pt, pterygoid; q, quadrate; qjh, quadratojugal horn; snca, supranarial caputegulum; sqh, squamosal horn. Color scheme after Arbour & Currie (2013a). Dorsal view of *N. kirtlandensis* modified after Arbour et al. (2014). *T. teresea (=Saichania chulsanensis* in Arbour, Currie & Badamgarav, 2014) and *M. ramachandrani* modified after Arbour, Currie & Badamgarav (2014).

**Figure 8:**
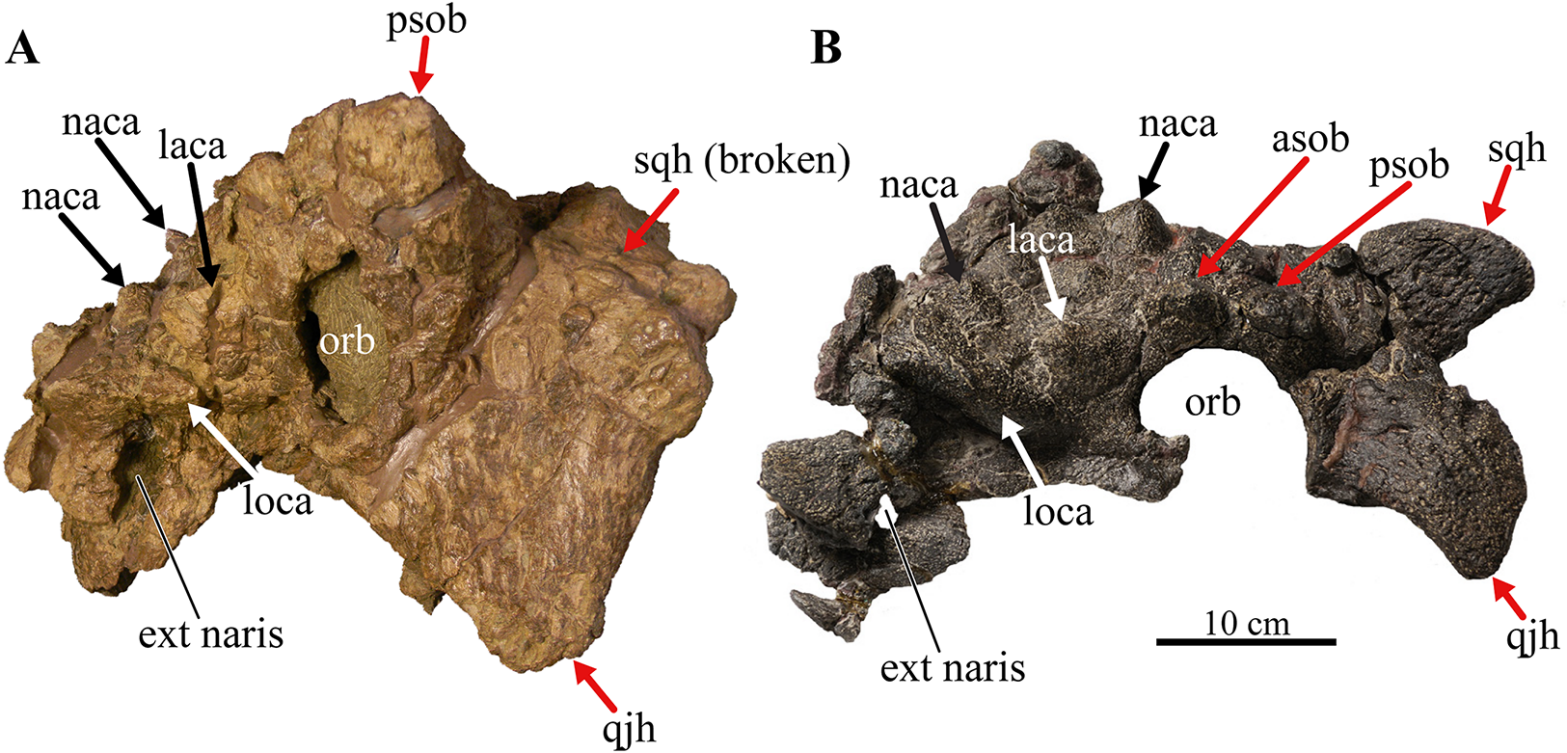
Skull of *Akainacephalus johnsoni* compared with *Nodocephalosaurus kirtlandensis*. Comparison of cranial features between closely related southern Laramidian taxa; (A), *Akainacephalus johnsoni* (UMNH VP 20202) from the Late Cretaceous Kaiparowits Formation of Utah; and (B), *Nodocephalosaurus kirtlandensis* (SMP VP-900) from the Late Cretaceous Kirtland Formation of New Mexico, in left lateral views. Various synapomorphies are shared with *N. kirtlandensis* (highlighted in black and white arrows) and includes “flaring nostrils”; enlarged, laterally projecting, loreal osteoderms that are situated directly dorsal to the external nares. Other synapomorphies include pyramid-shaped nasal and frontal osteoderms positioned on the dorsal regions of the skull. A number of significant differences have been observed between both specimens; in *A. johnsoni*, the anterior, and posterior supraorbital bosses form an enlarged element that is somewhat backswept, whereas in *N. kirtlandensis*, the posterior and anterior supraorbital bosses are clearly defined as individual osteoderms, and are much smaller in size. Additionally, the squamosal horn in *Akainacephalus* is very small but is prominent and tetrahedrally shaped in *Nodocephalosaurus*. The quadratojugal horn in *Akainacephalus* is massive, has a subtriangular morphology in lateral view and projects almost entirely ventral, whereas in *Nodocephalosaurus*, the quadratojugal horn is smaller and has a typical fin-shaped morphology. Study sites: asob, anterior supraorbital boss; ext naris, external naris; laca, lacrimal caputegulum; loca, loreal caputegulum; naca, nasal caputegulae; orb, orbit; psob, posterior supraorbital boss; qjh, quadratojugal horn; sqh, squamosal horn. Part B: Photograph of the holotype of *Nodocephalosaurus kirtlandensis* (SMP VP-900) is reproduced courtesy Robert M. Sullivan.

The dense array of tall, peaked caputegulae are positioned on a domed frontal-nasal region, that is, much more pronounced in *Akainacephalus johnsoni* compared to some Late Cretaceous ankylosaurids from Asia, including *M. ramachandrani* (INBR 21004), *Saichania chulsanensis* (Maryańska, 1977), and *Tarchia teresae* (Penkalski & Tumanova, 2017) (Fig. 7). Instead, the dome is morphologically similar to Late Cretaceous Laramidian ankylosaurids such as *Ankylosaurus magniventris* (Brown, 1908; Carpenter, 2004; Arbour & Mallon, 2017), *Anodontosaurus lambei* (CMN 8530; TMP 1997.132.1), *Euoplocephalus tutus* (e.g., ROM 1930; TMP 1991.127.1; UALVP 31), and *Ziapelta sanjuanensis* (Arbour et al., 2014). The dome covers most of the nasal region, extendingtowards the posteriormost part of the frontals, where it contacts the nuchal shelf. *Akainacephalus johnsoni* possesses two supranarial caputegulae that are morphologically similar to *Nodocephalosaurus kirtlandensis* (SMP VP-900); in both taxa, the supranarial caputegulae are anteriorly protruding, mediolaterally broad caputegulae, situated dorsally on the premaxilla (Figs. 3A–3D, 4A, 4C, 7A–7D and 8). Two flange-like and anteroposteriorly elongated loreal caputegulae cover the nares dorsally, each succeeded by a large, tetrahedral-shaped, prefrontal caputegulae. A small lacrimal caputegulum is positioned ventral to the prefrontal caputegulum. The postorbital horns are dorsoventrally tall, backswept, and project laterally in dorsal view. Only a partial squamosal horn is preserved but it is badly damaged and largely anatomically uninformative. The quadratojugal horns display a triangular morphology with a ventrally projecting apex and cover the majority of the ventral portion of the postocular region of the skull. Individual caputegulae are described below.

#### Supranarial caputegulae

The external nares in *Akainacephalus johnsoni* are oriented laterally and their position is placed posteriorly relative to the front of the snout, similar to *Nodocephalosaurus kirtlandensis* (Fig. 8). In most other ankylosaurids, including *M. ramachandrani* (INBR 21004), *Euoplocephalus tutus* (UALVP 31, AMNH FARB 5205), *Anodontosaurus lambei* (CMN 8350), *Tarchia teresae* (PIN 3142/250), and *Saichania chulsanensis* (MPC 100/151), the nares are oriented entirely anteriorly or anterolaterally and are dorsally and transversely ornamented with a thickened rim of ossification—the supranarial caputegulae (Arbour & Currie, 2013a). In *Akainacephalus johnsoni*, each naris is anteriorly ornamented with an anteroposteriorly broad, supranasal caputegulum that is positioned transversely and dorsolaterally along the premaxillary beak (Fig. 3). A similar caputegulum was described for *Nodocephalsaurus kirtlandensis* by Sullivan (1999). Given that the caputegulum is situated directly above the external nares, it seems appropriate to use the term supranarial caputegulum, which is consistent with the terminology used by Arbour & Currie (2013a, 2016). The surface texture of this large caputegulum is smooth, with some rugosity along the dorsal surface. The caputegulum is anteroposteriorly elongated (Figs. 4A and 4C) and contains a lateral margin that forms a longitudinal keel along the entire length of the caputegulum (Fig. 3). Posteriorly, the caputegulum contacts the anterior margin of the lacrimal portion of the circumorbital complex. Posterodorsally it is accompanied by a prominent, single, and tetrahedral-shaped lacrimal caputegulum with minimal rugose surface texture, which is present in *Nodocephalosaurus kirtlandensis* (SMP VP-900) and is referred to as the prefrontal caputegulum by Sullivan (1999). However, in *N. kirtlandensis* (SMP VP-900), the caputegulum is morphologically more conical rather than tetrahedral and is heavily pitted (Fig. 8). Anteriorly, each supranarial caputegulum contacts a large anterolaterally positioned caputegulum that forms the anterior-most ornamentation and is positioned transversely along the premaxilla, nearly identical to *N. kirtlandensis* (SMP VP-900).

#### Nasal caputegulae

At least eight, well-defined, caputegulae are preserved along the dorsal surface of the nasal region and are arranged in a symmetrical pattern, separated by a distinct midline row of pyramidal caputegulae (Figs. 4A and 4C). This symmetrical arrangement of caputegulae contrasts with those observed in Asian taxa such as *M. ramachandrani* (INBR 21004), *Saichania chulsanensis* (Maryańska, 1977), and *Tarchia teresae* (Penkalski & Tumanova, 2017), where the nasal caputegulae are symmetrically arranged in a paired configuration, lacking a midline row of caputegulae. Nasal caputegulae in Laramidian ankylosaurids such as *Euoplocephalus tutus* (ROM 1930, TMP 1991.127.1, UALVP 31), *Anodontosaurus lambei* (CMN 8530), and *Ziapelta sanjuanensis* (Arbour et al., 2014) are somewhat symmetrically arranged in a mosaic pattern. The largest caputegulae in *Akainacephalus johnsoni* form a sagittal midline row that are mostly hexagonal in morphology and terminate dorsally in a distinct apex (Figs. 3A and 5B), whereas in *Nodocephalosaurus kirtlandensis* (SMP VP-900) the nasal caputegulae are conical (Fig. 8). Both morphologies contrast with the nasal caputegulae in *M. ramachandrani* (INBR 21004), which are hexagonal, polygonal, and square at the base, and have well-defined facets that terminate in an apex. In *Akainacephalus johnsoni*, a single row of both polygonal- and pyramid-shaped caputegulae is situated laterally of the sagittal midline row of caputegulae (Figs. 4A, 4C, 7A and 7B). The anterior-most nasal caputegulae (the internarial caputegulum in Arbour, Currie & Badamgarav, 2014: fig. 4F) are anteriorly bordered by the supranarial caputegulae. All nasal caputegulae show a surface texture combination of deep pits and vascular grooves.

#### Loreal caputegulae

A very characteristic, large, and laterally oriented loreal caputegulum (= postmaxillary/lacrimal ridge in Sullivan, 1999) is positioned dorsally above the external nares where it ascends anteriorly and “flares” out laterally (Fig. 3). The loreal caputegulum extends posteriorly and contacts the anterior margin of the circumorbital complex, enveloping the external nares along the anterodorsal and posterior margins. Sullivan (1999) referred to this particular loreal caputegulum in *Nodocephalosaurus kirtlandensis* (SMP VP-900) as the postmaxillary/lacrimal ridge, but this term is somewhat misleading because the caputegulum in *Akainacephalus johnsoni* is positioned directly dorsal to the maxilla and obscures the lacrimal. The lateral projection of the nasal caputegulum forms a thickly rimmed, overhanging shelf, producing a very characteristic flaring nostril in lateral view. This condition is currently only observed in *Akainacephalus johnsoni* and *Nodocephalosaurus kirtlandensis* (Sullivan, 1999).

#### Prefrontal caputegulae

A distinct, tetrahedral/subtriangular caputegulum with a laterally projecting, keeled apex is positioned on the lateral side of each prefrontal (Figs. 4A and 4C). The caputegulum is directly anterior to the anterior-most supraorbital boss and dorsal to the flanged nasal caputegulum, and shows a morphology and position similar to the prefrontal caputegulae in *M. ramachandrani* (INBR 21004) and *Saichania chulsanensis* (Maryańska, 1977: fig. 5; Arbour, Currie & Badamgarav, 2014: figs. 4E–F and 5D). In UMNH VP 20202 it does not form a continuous surface with the supraorbital bosses, in contrast with *M. ramachandrani*. It is situated directly anterior to the circumorbital complex and dorsal to the supranarial caputegulum and lacrimal. The surface texture is similar to that of the supranarial caputegulum and is relatively smooth with some vascularity but no pitted textures.

#### Frontal caputegulae

Many caputegulae are eroded away but seven distinct caputegulae are preserved across the frontal region of the skull and are arranged in a mosaic pattern (Figs. 4A and 4C). The posterior-most caputegulum is the largest and positioned medially. It has a mediolaterally oblong, somewhat hexagonal base and a bluntly bulbous morphology with relatively smooth surface texture. This caputegulum is laterally and anteriorly surrounded by six smaller caputegulae. The morphology of the smaller surrounding caputegulae is very different from the large, posterior caputegulum, but similar to the nasal caputegulae; they preserve irregularly polygonal-shaped base, are sharply conical rather than bulbous, and possess a rugose and pitted surface texture. The arrangement of the frontal caputegulae in *Akainacephalus* appears similar to *Anodontosaurus lambei* (TMP 1997.59.1 (Arbour & Currie, 2013a: fig. 4.)) and *Ankylosaurus magniventris* (Brown, 1908; Carpenter, 2004; Arbour & Mallon, 2017); however, the morphology of these smaller caputegulae is more similar to those observed in *Nodocephalosaurus* (SMP VP-900) (Fig. 7C), and to some extent *M. ramachandrani* (INBR 21004) (Fig. 7G) and *Zaraapelta nomadis* (Arbour, Currie & Badamgarav, 2014), although the latter two taxa display a more pyramidal than conical morphology. Frontal caputegulae in other Asian taxa such as *Tarchia* (Tumanova, 1977; Penkalski & Tumanova, 2017) and *Saichania* (Maryańska, 1977) are morphologically very different from *Akainacephalus johnsoni* and display a more rectangular base and a nearly flat or slightly bulbous dorsal surface. In Laramidian ankylosaurid dinosaurs, including *Euoplocephalus tutus* (Arbour & Currie, 2013a) and *Ziapelta sanjuanensis* (Arbour et al., 2014), very few or no frontal caputegulae are known, either because they did not preserve or perhaps these taxa lack extensive cranial ornamentation in this region.

#### Nuchal caputegulae

The nuchal shelf forms a dorsoventrally raised, tabular shelf, similar to *Minotaurasaurus ramachandrani* (INBR 21004), *Tarchia kielanae* (Arbour, Currie & Badamgarav, 2014; Penkalski & Tumanova, 2017), *Tarchia teresae* (Penkalski & Tumanova, 2017), *Saichania chulsanensis* (Maryańska, 1977), and some specimens of *Euoplocephalus tutus* (ROM 1930), *Oohkotokia horneri* (MOR 433), and *Anodontosaurus lambei* (AMNH FARB 5238). In *Akainacephalus johnsoni*, the nuchal shelf shows little rugosity, but is damaged in several places across the dorsal surface and a large portion is broken away from the left side, leaving a transversely oblong cavity (Figs. 4A and 4C). Nuchal caputegulae are present on the posterior-most portion of the nuchal shelf. A total of three, poorly preserved caputegulae are visible (Fig. 4A), and their morphology varies between subrounded with a small apex to elongate polygonal with a small transverse dorsal ridge. This condition is dissimilar from other ankylosaurids such as *Ziapelta sanjuanensis* (Arbour et al., 2014), *Zaraapelta nomadis* (MPC D100/1338 (Arbour, Currie & Badamgarav, 2014)), *Pinacosaurus mephistocephalus* (IMM 96BM3/1 (Godefroit et al., 1999)), and *P. grangeri* (MPC 100/1014 (Tumanova, 1987)), in which the nuchal shelf and the nuchal caputegulae are flat. The surface texture of the nuchal caputegulae in *Akainacephalus johnsoni* is smooth with shallow pitting. A distinct furrow separates the nuchal caputegulae from the posterior supraorbital bosses.

#### Circumorbital complex

The large circumorbital complex consists of a series of co-ossified ornamental elements that surround the orbital cavity and are best preserved on the left lateral side of the skull (Fig. 3A). The complex comprises a large supraorbital horn, a small lacrimal caputegulum, a semicircular jugal osteoderm, and the thickened rim along the posterior margin of the orbital. The anterior- and posterior supraorbital caputegulae appear fused together, forming a large, and tall, postorbital horn with a prominent apex that projects posterolaterally, covering the entire dorsal surface of the supraorbital (Figs. 3 and 4);a condition unique to *Akainacephalus johnsoni*. In anterior and posterior view (Fig. 5), the postorbital horn projects dorsolaterally and laterally in dorsal view (Figs. 4A and 4C), forming the transversely widest part of the skull as preserved. Usually, cranial elements such as the squamosal horns and quadratojugal horns exceed the width of the postorbital horns as in *M. ramachandrani* (INBR 21004), *Tarchia teresae* (Penkalski & Tumanova, 2017), *Pinacosaurus mephistocephalus* (Godefroit et al., 1999). This cannot be evaluated in *Akainacephalus johnsoni* because the squamosal horns are badly damaged, and the quadratojugal horns are more vertically oriented compared to other ankylosaurid taxa (e.g., *M. ramachandrani* [INBR 21004], *Euoplocephalus tutus* [AMNH FARB 5405, UALVP 31], *Ankylosaurus magniventris* [AMNH FARB 5214], and *Anodontosaurus lambei* [CMN 8530]). In dorsal view, the postorbital horns are large and triangular with a posterolaterally projecting apex (Fig. 4A). The surface texture is coarsely rugose and bulbous. In lateral view, the apex projects posterodorsally and is less rugose compared to its dorsal surface. The lacrimal caputegulum is positioned ventral to the supraorbital boss and comprises a small tabular caputegulum with a much smoother surface texture. The jugal caputegulum forms the ventral-most border of the circumorbital complex and has a semilunate, dorsally concave morphology that cradles the orbit and possesses a relatively smooth surface texture. The caputegulum extends laterally and results in a small shelf. Posteriorly, several small, tabular caputegulae complete the circumorbital complex. They are more rugose than the lacrimal and jugal caputegulae but less so than the supraorbital boss.

#### Quadratojugal horns

The quadratojugal horns cover the majority of the ventral postocular region of the skull (Fig. 3). Their morphology and orientation are unusual in that they represent an asymmetrical triangle with a vertically positioned apex, which is somewhat similar to *Zaraapelta nomadis* (Arbour, Currie & Badamgarav, 2014) and *Shamosaurus scutatus* (Tumanova, 1983), except that the apices in *Z. nomadis* project lateroventrally, and *Akainacephalus johnsoni* lacks the presence of interstitial postocular ossicles, separating the quadratojugal from the squamosal horn and orbit. In anterior and posterior view, the quadratojugal horns project nearly vertically, which appears to be close to the original anatomical position of the horns, given that little deformation and breakage of other (basicranial) elements is expressed in this area of the skull. In lateral view, the anterior part of the quadratojugal cradles the ventral margin of the jugal portion of the circumorbital complex, whereas the posterior end extends posterodorsally and contacts the posterior margin of the circumorbital complex. The extent of the posterodorsal boundaries of the quadratojugal horns are poorly defined and blend together with the squamosal, making it difficult to assess their exact position. The quadratojugal horns are nearly straight or slightly convex laterally and have an overall smooth surface texture, accompanied by dorsoventral-oriented furrows or vascular grooves (Fig. 3). The quadrates are visible in lateral view, anterior to the quadratojugal horns, a condition similar to *Nodocephalosaurus kirtlandensis* (SMP VP-900), *Gobisaurus domoculus* (Vickaryous et al., 2001), *Shamosaurus scutatus* (Tumanova, 1983), and *Tarchia teresae* (Penkalski & Tumanova, 2017). In all other ankylosaurids (e.g., *Saichania chulsanensis*, *P. grangeri*, *Euoplocephalus tutus*, *Zuul crurivastator*, and *Anodontosaurus lambei*), the quadrates are obscured by the quadratojugal horns.

#### Squamosal horn

Only a partial right squamosal horn is preserved (Fig. 3B), but is largely broken, limiting the amount of observable anatomical detail. The right squamosal horn forms the posterior-most caputegulum on the skull (Fig. 3) and is positioned directly posterior to the supraorbital boss. Various Asian (e.g., *Tarchia kielanae* (Maryańska, 1977), *Saichania chulsanensis* (Maryańska, 1977), *Zaraapelta nomadis* (Arbour, Currie & Badamgarav, 2014), *Minotaurasaurus ramachandrani* [INBR 21004]) and most Laramidian taxa (e.g., *Anodontosaurus lambei* [CMN 8530], *Euoplocephalus tutus* [e.g., ROM 1930], *O. horneri* [MOR 433], *Ankylosaurus magniventris* (Carpenter, 2004), *Ziapelta sanjuanensis* (Arbour et al., 2014)) display a clear separation between the quadratojugal and squamosal horns, a space that is sometimes occupied by postocular ossicles and visible in several specimens of *Anodontosaurus lambei* (CMN 8530, NHMUK R4947, TMP 1997.132.1) and *Zaraapelta nomadis* (Arbour, Currie & Badamgarav, 2014), but this area is poorly defined in *Akainacephalus johnsoni* (Figs. 3 and 7). The left squamosal horn is not preserved and the area is damaged.

#### Mandibular caputegulum

**Figure 9:**
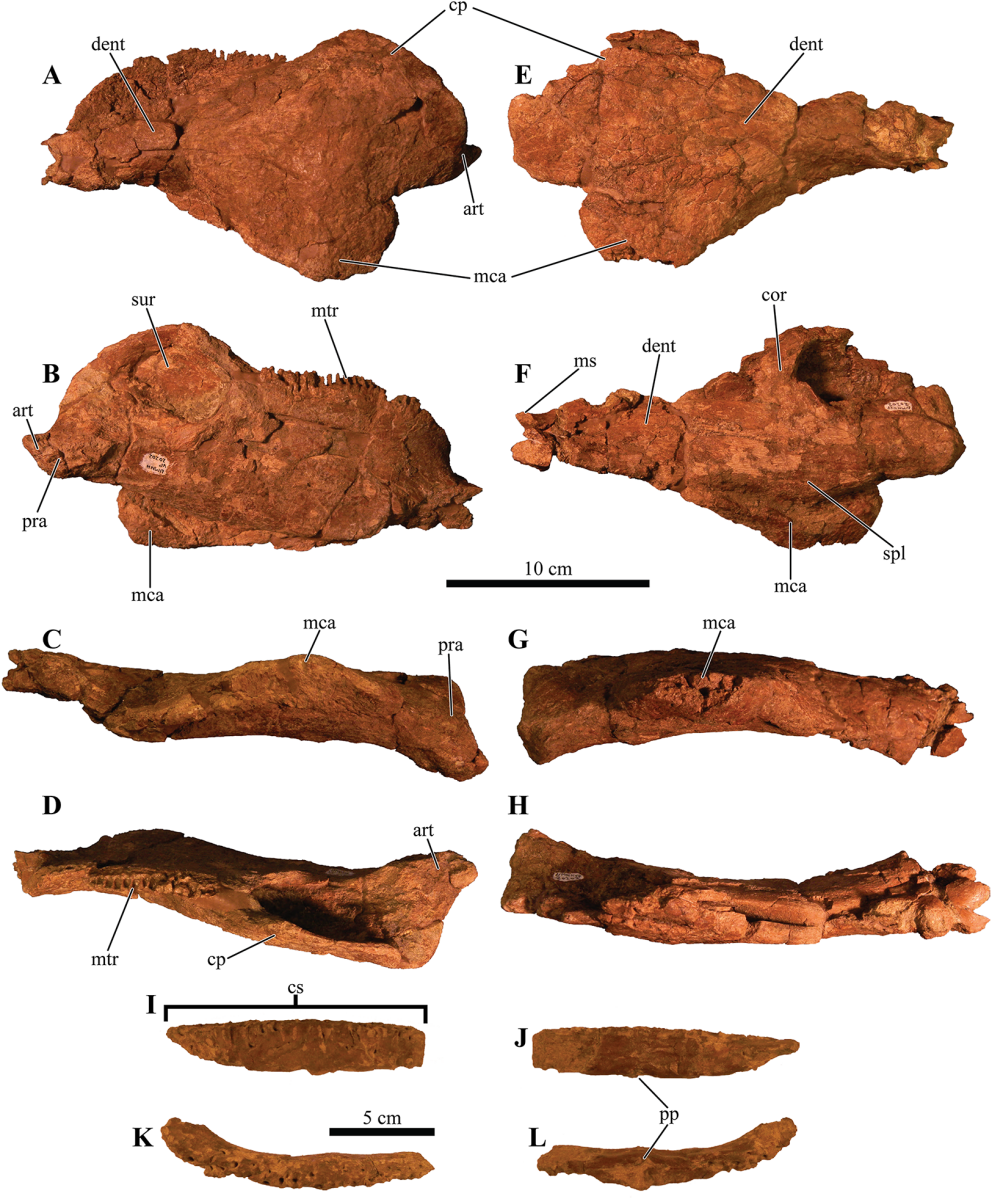
Mandibles and predentary. Photographs of the mandibles and predentary of *Akainacephalus johnsoni* (UMNH VP 20202). Left mandible in (A), lateral; (B), medial; (C), ventral; and (D), dorsal views. Right mandible in (E), lateral; (F), medial; (G), ventral; and (H), dorsal view. Predentary in (I), anterior; (J), posterior; (K), dorsal; and (L), ventral views. Study sites: cp, coronoid process; cs, predentary cutting surface; mos, mandibular osteoderm; ms, mandibular symphysis; mtr, mandibular tooth row; pp, predentary protuberance.

An anteroposteriorly elongated and dorsoventrally deep mandibular caputegulum forms the ventral border of the mandible (Fig. 9). Morphologically, the caputegulum is subtriangular with a blunt keel and a near-ventral projection; a condition unique to *Akainacephalus johnsoni*. The caputegulum is positioned along the ventrolateral margin of the jaw, covering the ventral, and lower half of the lateral portion of the angular. The total length of the mandibular caputegulum covers >50% of the entire anteroposterior length of the lower jaw. The mandibular caputegulum is short compared to other ankylosaurid taxa, including specimens of *Euoplocephalus tutus* (AMNH FARB 5403 and 5405), *M. ramachandrani* (INBR 21004), and to some extent, *Tarchia teresae* (=*Saichania chulsanensis* (Arbour, Currie & Badamgarav, 2014)), in which the caputegulae are dorsoventrally narrow and encompass nearly the entire length of the mandible. Although the orientation of the mandibular caputegulum is unique to *Akainacephalus johnsoni*, morphologically it shares some similarities to the mandibular caputegulum seen in the holotype of *Saichania chulsanensis* (MPC 100/151 (Arbour, Currie & Badamgarav, 2014)). In anterior and posterior view, the mandibular caputegulum is oriented ventrolaterally and extends well below the dentary and angular, forming the ventral-most portion of the jaw. Posteriorly, the caputegulum is convex and curves upward in a lobe-shaped morphology. It terminates against, and contacts with, the ventral border of the surangular. A distinct longitudinal furrow delineates the medial border of the mandibular caputegulum from the angular. The ventral surface is mediolaterally broad and horizontally flat. The external surface texture is smooth and shows no signs of furrowing and pitted textures. Measurements for individual elements are summarized in Supplemental Material S4.

### Bones of the dermatocranium

#### Premaxillae

The premaxillae form a broad U-shaped beak in ventral view (Figs. 4B and 4D), similar to *Euoplocephalus tutus* (ROM 1930; TMP 1997.132.1 (Arbour & Currie, 2013a: fig. 6A–B)), *Anodontosaurus lambei* (CMN 8530), and *Ziapelta sanjuanensis* (Arbour et al., 2014). In *Ankylosaurus magniventris* (AMNH FARB 5214), *Pinacosaurus mephistocephalus* (Godefroit et al., 1999), *P. grangeri* (Hill, Witmer & Norell, 2003), *Tarchia teresae* (Penkalski & Tumanova, 2017), *Saichania chulsanensis* (Maryańska, 1977), and *M. ramachandrani* (INBR 21004), the premaxillae slightly taper anteriorly, resulting in a narrower beak. In anterior view, the rostral portion of the premaxilla is transversely wide and tall, dorsally bound by a distinct transverse and ventrally concave-oriented nasal ridge which is bisected by two supranarial caputegulae (Fig. 5B). The ventral margin that forms the premaxillary tomium is eroded, but preserved areas reveal a ventrally convex morphology for each premaxilla. A significant portion of the right premaxillary tomium is broken. Anteriorly, the midline possesses the typical interpremaxillary suture (*sensu* Vickaryous, 2001) and is characterized by a small midline notch (*incisura premaxillaris*; Vickaryous, 2001) (= interpremaxillary notch; Sereno, 1999) bisecting the left and right premaxillae. In ventral view, the buccal margin of the premaxilla is U-shaped and shallow. The premaxillary palate is wider than it is long. The lateral halves of the premaxillary palate are slightly concave ventrally but become more convex medially, a morphology similar to *Euoplocephalus tutus* (ROM 1930) and *Ankylosaurus magniventris* (Carpenter, 2004: fig. 4C). The majority of the vomer is broken away along the rostral part of the skull and only a dorsal remnant that articulates with the roof of the oral cavity remains. The premaxilla-maxilla contact is the widest part of the premaxillae in ventral view. In lateral view, it borders the ventral margin of the external nares. The ventral cutting surface is convex and forms a distinct premaxillary notch along the midline, separating both premaxillae. This notch is clearly visible in ventral view and continues posteriorly to form a small tear drop-shaped foramen (Figs. 4B and 4D). The ventral portion of the premaxillary beak is anteroposteriorly shorter than in other ankylosaurids (e.g., *M. ramachandrani* [INBR 21004]) and contacts the anteriormost alveolar cavities of the maxillae. The premaxillary tomial crest is short and terminates well before the anterior-most maxillary alveolar cavity.

#### Maxillae

The maxillae are poorly preserved, and most of the surface textures and margins have been eroded to some extent. However, both maxillae in *Akainacephalus johnsoni* are unusually well exposed in lateral view, revealing important anatomical features (Fig. 3). In lateral view, the anterior margin is concave and borders the posterior and partial ventral portion of the external nares until it contacts the premaxilla. The ventral portion of the anterior half of the maxilla is convex but becomes concave along the posterior half, where it contacts the anteroventral margin of the circumorbital complex. A large but shallow sulcus forms the majority of the posterior half of the maxilla. In most ankylosaurids (e.g., *Pinacosaurus grangeri* [MPC 100/1014 (Hill, Witmer & Norell, 2003: fig. 4)], *Saichania chulsanensis* [MPC 100/151], *M. ramachandrani* [INBR21004], *Euoplocephalus tutus* [e.g., TMP 1991.127.1], *Anodontosaurus lambei* [CMN 8530], and *Ankylosaurus magniventris* [AMNH FARB 5214 (Carpenter, 2004: fig. 4C)]) only the ventral-most portion of the maxilla is visible in lateral view. Lateral and ventrally extending nasal and lacrimal caputegulae obscure the maxillae in the aforementioned taxa, but *Akainacephalus johnsoni* possess no such lateral covering, instead, the only ornamentation that is present along the maxilla is contributed dorsally by the supranasal caputegulum, and posteriorly by the anteroventral margin of the circumorbital complex (Fig. 3), similar to *Nodocephalosaurus kirtlandensis* (Sullivan, 1999). Approximately 75% of the entire tomial crest is located on the maxillae and terminates just dorsal of the ectopterygoid. Breakage and erosion have damaged both tooth rows, and the four to six anterior-most alveolar cavities on the right maxilla are eroded away. All the alveolar cavities on the posterior half of the left maxilla are eroded away, preserving only the anterior alveoli. A transverse fracture displaces the posterior half of the preserved alveoli, offsetting them medially. Preserved alveolar cavities suggest that each maxilla would have held at least 16 teeth during life. Both maxillae are laterally concave and together form an hourglass configuration, a condition typical for ankylosaurid and nodosaurid dinosaurs. The anterior half of the maxilla forms the widest part of the element and becomes the ventral border of the external nares. Posteriorly, the width of the maxilla decreases, and it contacts with the dorsal surface of the ectopterygoid. In lateral view, a deep sulcus is positioned between the supranarial caputegulum and the posterior half of the maxilla. Ventrally, the anterodorsal secondary palate forms the lingual part of the maxilla and is dorsally depressed compared to its anteriorly bordering premaxillary shelf (Figs. 4B and 4D).

#### Nasals

The nasals are completely obscured by the co-ossification of caputegulae along their entire dorsal and lateral surfaces. Ventrally, they are obscured by the maxillary secondary palate (Figs. 4B and 4D). Observations of the nasals have been made with the aid of CT scans where features are obscured. The internasal septum is morphologically similar to that described in *Pinacosaurus grangeri* (Maryańska, 1977; Hill, Witmer & Norell, 2003) and forms a paired element that resembles elongate wings that project ventrally and are formed by the medial margins of the nasal bones. Together, they comprise the insertion space for the vomer. Posteriorly, the internasal septum forms a steeply descending ventral flange that articulates with the midline of the pterygoid complex. Anteriorly, the septum expands transversely as well as anteroposteriorly, resulting in an elliptical, bulging process that articulates along the midline on the posterior margin of the premaxillae. The choanae are deep and have bony fragments encased in sandstone matrix in the dorsal-most regions, which might belong to the roof of the nasals.

#### Lacrimals

In ankylosaurs, the lacrimals form the anterior border of the orbit and circumorbital complex, and contact the maxillae anteroventrally, the supraorbitals posterodorsally, and the jugals posteroventrally (Vickaryous, 2001). Only a few ankylosaurian dinosaurs, such as juvenile *Pinacosaurus grangeri* (Vickaryous, 2001; Burns et al., 2011) and *Kunbarrasaurus ieversi* (Leahey et al., 2015), display clearly identifiable lacrimals, which form the anterior-most circumorbital bones. In *Akainacephalus johnsoni*, the lacrimals cannot be identified as distinct elements because of the presence of remnants of unprepared matrix along the medial and ventral portions of this region (Fig. 3). In addition, co-ossification of the rugose secondary surface on the lateral side fully obscures the elements. In his description of *Nodocephalosaurus kirtlandensis*, Sullivan (1999) mentioned the presence of a prominent lacrimal, comparable to *P. grangeri* (Vickaryous, 2001). In *Akainacephalus johnsoni* the lacrimals are poorly visible laterally, which is possibly due to deformation. Dorsolaterally, the lacrimals are ornamented with a tetrahedral-shaped lacrimal caputegulum (Fig. 3), which has a conical morphology in *N. kirtlandensis* (Sullivan, 1999). In contrast, in *Nodocephalosaurus kirtlandensis* the lacrimal is well defined in lateral view and has a rugose surface texture (Sullivan, 1999: fig. 2). In *Akainacephalus johnsoni*, the long axis of the lacrimals is oriented anteromedially in lateral view.

#### Postorbital

The postorbital forms the posterior-most contribution to the circumorbital complex and contacts anteriorly with the supraorbital and ventrally with the jugal (Fig. 3). Its exact morphology cannot be determined because it is co-ossified with caputegulae along its exterior surface and obscured by matrix medially. The ornamented lateral surface that forms the posterior border of the orbit is continuous with the posterodorsal ornamentation of the jugal. It forms a thin and narrow, dorsally projecting, and anteriorly concave wedge that expands dorsally and longitudinally where it contacts the ventral border of the supraorbital boss. The pre- and postocular walls border the orbital cavities but are only visible with the aid of CT data (Figs. 6B and 6C) because the orbits are infilled with matrix. They form a transverse wall of bone, occluding the anterior and posterior borders of the orbit.

#### Jugals

The right jugal is damaged by breakage and erosion (Fig. 3B). The left jugal is better preserved and comprises a small and transversely broad element that contacts the anterior-most part of the quadratojugal and the posterior margin of the maxilla (Fig. 3A). The long axis of the element is anteromedially oriented. The jugal curves anteromedially and ventrally towards the pterygoid, suggesting a contact between both elements; however, this area is damaged and a suture confirming this contact could not be observed. In lateral view, the jugal forms a dorsally concave semilunate shelf that is laterally ornamented with a small ridge of rugose co-ossified bone that forms the ventral border for both the orbit and the circumorbital complex.

#### Squamosals

Co-ossification along the lateral (Fig. 3) and dorsal surfaces (Figs. 4A and 4C) of the squamosal, and the paroccipital processes posteriorly, fully obscure the squamosal.

#### Quadratojugals

The quadratojugal forms the anterior extension of the quadratojugal horn. The left quadratojugal is best preserved. It is a small, anteriorly projecting wall of bone that appears as a small point in lateral view. It curves medially along its posterior half and contacts the lateral distal shaft and condyle of the quadrate. The anterior half contacts the jugal along its dorsal surface (Fig. 3).

#### Palatines

Only fragmentary remains of the posteroventral portion of the palatines are preserved, making it difficult to provide detailed anatomical descriptions of this element. These remnants are positioned medial to the maxillae, lateral to the internasal septum and jugal, and anteromedial to the pterygoids (Figs. 4B and 4D). The anterior margin is concave and shallow, forming the posterior border of the choanae. Posteriorly, the palatines reside entirely against the pterygoids to form a solid surface. Some taxa, such as *Pinacosaurus grangeri* (Hill, Witmer & Norell, 2003), *Anodontosaurus lambei* (CMN 8530), and *Euoplocephalus tutus* (e.g., Arbour & Currie, 2013a, TMP 1997.132.1, and ROM 1930) have a posteriorly located postpalatal fenestra or lateral palatal aperture which are absent in UMNH VP 20202.

#### Ectopterygoid

The ectopterygoid forms a poorly preserved, small, wedge-shaped bone that is morphologically consistent with other ankylosaurid dinosaurs (e.g., *Ankylosaurus*) and is positioned ventromedially to the left pterygoid flange (= pterygoid wing in Arbour & Currie, 2013a), and lateral to the maxilla (Figs. 4B and 4D). Similar to the description of the ectopterygoid by Vickaryous (2001), it contributes to the rostral border of the suborbital fenestra and the caudoventral secondary palate. Significant breakage is present along the ectopterygoid-pterygoid complex, resulting in the loss of the right ectopterygoid and the lateral-most portion of the right pterygoid-ectopterygoid articular surface.

#### Pterygoids

The pterygoid complex is preserved along its posterior region and a small portion of the left pterygoid flange is visible, but it is completely missing on the right side (Figs. 4B and 4D). The pterygoid shields are ventrally concave and medially curve downward to form a blunt keel along the posterior margin. Together with the basipterygoids, they form a large, anteriorly tapering, teardrop-shaped pterygoid vacuity, similar to *Pinacosaurus grangeri* (Hill, Witmer & Norell, 2003), *M. ramachandrani* (INBR 21004), *Euoplocephalus tutus* (e.g., TMP 1997.132.1; ROM 1930), and *Zuul crurivastator* (ROM 75860). In *Saichania chulsanensis* (Maryańska, 1977), this vacuity is much smaller. The medial margins along the anterior portion of the pterygoid shields do not contact each other but extend anteriorly where they contact the internasal septum. Laterally, the pterygoid shields contact the medial margin of the quadrate shaft. The anterolateral half becomes the pterygoid flange, is ventrally convex, and forms the contact for the ectopterygoid. The pterygoid flanges extend anterior to the quadratojugal horn and are visible in lateral view.

### Bones of the chondrocranium

#### Laterosphenoid

The laterosphenoids are not visible in external view because they are entirely obscured by matrix. CT scans reveals little detail, but do allow visualization of the position of cranial fenestrae that accommodated CN IX and X (Fig. 6C), confirming the position of the laterosphenoid. Additionally, the large fenestra ovalis (*fenestra vestibuli*) is clearly visible and positioned dorsally and anterior to the aforementioned fenestrae (Fig. 6C), demarcating the boundary between the posterior border of the prootic and anterior border of the opisthotic bones. The laterosphenoid forms the dorsal accommodation space for cranial fenestrae IX and X and contacts the anterior surface of the supraoccipital. Transverse plane CT imaging of the laterosphenoid show it broadening posterolaterally and transitioning into the exoccipitals, but the boundary between these elements is not clear. The laterosphenoid forms the lateral walls of the posterior portion of the endocranial cavity.

#### Prootic

Similar to the laterosphenoid, the prootic is not distinguishable in external view due to heavily eroded external surfaces and partial obscural by matrix, making anatomical descriptions and comparison with other taxa difficult. Internally, CT scan data reveal little anatomical detail as well, because this region is severely fractured (Figs. 6A–6E). However, these CT data do provide information regarding the position of the prootic with respect to the endocranial cavity and, together with the laterosphenoid and opisthotic, indicate it forms the lateral portion of the endocranial cavity. Anatomically, the prootic is positioned anterior to the opisthotic and posterior to the laterosphenoid (Vickaryous, Maryańska & Weishampel, 2004), but sutures between these elements are fully obliterated. Nonetheless, the presence of the fenestra ovalis (Fig. 6C) confirms the position of the prootic in UMNH VP 20202.

#### Opisthotic

Only the anterior and ventral portions of the left opisthotic are exposed. In ventral view it displays a wedge-like morphology, widest medially and tapering laterally until it forms a continuous surface with the shaft of the exoccipital process. A small depression on the ventral side of the opisthotic, just anterior to the exoccipital, suggests the presence of a foramen. The exoccipitals and paroccipital processes are transversely straight and dorsoventrally short, forming a mediolaterally long structure along the majority of the occiput which extends from the lateral borders of the foramen magnum onto the quadrates. Dorsally and ventrally they are slightly concave and only the dorsolateral margin contacts the parietals. Ventrally, the lateral surfaces contact the posterior surfaces of the quadrates. Dorsolateral to the foramen magnum two broken and isolated buttresses form the remnants of a prominent pair of caudoventromedially oriented transverse nuchal crests (= oval tuberosities of Nopcsa, 1928; and proatlas facet of Carpenter, 2001). The inclination of the nuchal crest corresponds to the orientation of the foramen magnum. Dorsal to each of the nuchal crests are two shallow, mediolaterally transverse furrows. The long axis of the exoccipitals is mediolaterally oriented in posterior view and laterally oriented in dorsal view, a condition common in other ankylosaurid dinosaurs from both Asia and western North America, including *Saichania chulsanensis* (Maryańska, 1977), *Tarchia teresae* (Penkalski & Tumanova, 2017), *M. ramachandrani* (INBR 21004), *Euoplocephalus tutus* (Arbour & Currie, 2013a, TMP 1997.132.1, ROM 1930), *Anodontosaurus lambei* (CMN 8530), *Ankylosaurus magniventris* (Carpenter, 2004), and *Oohkotokia horneri* (MOR 433). In posterior view, the distal ends of the paroccipital processes are rounded, bend ventrally and are dorsoventrally expanded (Fig. 5A).

#### Supraoccipital

The supraoccipital is a single subrectangular element that laterally contacts the exoccipital processes and dorsally contacts the parietals (Fig. 5A). It is dorsoventrally broad, similar to *M. ramachandrani* (INBR 21004), *Saichania chulsanensis* (Carpenter et al., 2011), *Zaraapelta nomadis* (Arbour, Currie & Badamgarav, 2014), and *Euoplocephalus tutus* (ROM 1930). Its ventral border preserves a small posteriorly oriented ridge that forms the roof of the foramen magnum. The dorsal contact with the parietals is poorly exposed. Much of the original posterior surface texture is eroded away and distinct boundaries and sutures are no longer visible. Only small remnants of the proatlas facets are preserved and would extend posteriorly to the same extent as the occipital condyle to form the articular surface for the atlas. There appears to be no indication of posterior thickening along the ventral margin of the supraoccipital, a condition that is unique to *M. ramachandrani*.

#### Basioccipital

The basioccipital is an unpaired, transversely broad midline element that contacts the basisphenoid anteriorly and opisthotic laterally (Figs. 4B and 4D). The ventral surface of the basioccipital is transversely convex and anteroposteriorly concave and lacks a distinct foramen located on the ventral midline, similar to *Zaraapelta nomadis* (Arbour, Currie & Badamgarav, 2014). A basioccipital foramen is clearly present in some other ankylosaurids, including *Saichania chulsanensis* (Maryańska, 1977; Arbour, Currie & Badamgarav, 2014) and *Nodocephalosaurus kirtlandensis* (Sullivan, 1999). Posteriorly, the basioccipital is widest and extends laterally beyond the occipital condyle (Fig. 4B), whereas in *N. kirtlandensis* the basioccipital is laterally concave, not extending beyond the occipital condyle (Sullivan, 1999: fig. 5B). The basioccipital-basisphenoid complex in *Akainacephalus johnsoni* tapers anteriorly along their lateral margins, preserving a triangular morphology. However, both elements are heavily eroded along their lateral margins, so this shape is not entirely original (Figs. 4B and 4D). In lateral view, the basioccipital in *Akainacephalus johnsoni* forms a shallow ventrally concave depression, whereas in *Nodocephalosaurus kirtlandensis* there is a distinct, deeply excavated, and saddle-shaped concavity (see Fig. 5A in Sullivan, 1999). The dorsal portion of the basioccipital contacts the opisthotic and exoccipital bones and also forms the ventral accommodation space for three cranial foramina that would house CN IX, X, and XI (Fig. 6C). The occipital condyle lacks a neck that elevates it from the rest of the basioccipital and therefore contributes to the ventral-most border of the foramen magnum. Most of the ventral surface of the occipital condyle is eroded away but the overall morphology is reniform and it is oriented posteroventrally.

#### Basisphenoid

Anterior to the basioccipital is the basisphenoid; an anteroposteriorly short and unpaired midline element that contacts the pterygoid complex anteriorly with the basipterygoid processes, the basioccipital posteriorly, and the laterosphenoid and prootic dorsally (Figs. 4B and 4D). Two thin but long basipterygoid processes are positioned along the anterolateral margin of the basisphenoid and have an anterolateral orientation. The basipterygoids articulate with the posterior surface of the pterygoids. In ankylosaurs, contact between the basioccipital and basisphenoid is often characterized by presence of a mediolateral transversely running basal tuberum, but superficial erosion along the ventral surface has removed any evidence of this structure in UMNH VP 20202. Instead, the ventral surface of the basisphenoid forms a continuous surface with the basioccipital and is longitudinally strongly concave and transversely convex, similar to *Nodocephalosaurus kirtlandensis* (SMP VP-900), *M. ramachandrani* (INBR 21004), and *Euoplocephalus tutus* (e.g., TMP 1997.132.1, UALVP 31). The anterior-most portion of the basisphenoid curves sharply ventral to form a vertical projection. Erosion has removed most of the lateral margins of the basisphenoid.

### Bones of the splanchnocranium

#### Quadrates

The quadrates are relatively robust and tall elements that have a strong anterior inclination of >60° in lateral view. The dorsal half contacts with the exoccipitals posteriorly, but the exoccipitals do not appear to be fused with the quadrates. Laterally and medially the quadrate shaft expands to contact the medial face of the quadratojugals and the lateral face of the pterygoids, respectively. Ventrally, the medially expanded portion of the shaft possesses a sharp keel (Figs. 4B and 4D). Both the quadrates are clearly visible in lateral view and the quadrate condyles are positioned anterior to the quadratojugal horns (Fig. 3). In most Laramidian (e.g., *Euoplocephalus tutus* [e.g., ROM 1930, TMP 1991.127.1, UALVP 31), *Ankylosaurus magniventris* (Carpenter, 2004), *Anodontosaurus lambei* [CMN 8530]) and Asian ankylosaurids (e.g., *Tarchia teresae* (Penkalski & Tumanova, 2017), *Saichania chulsanensis* (Maryańska, 1977)) the quadrates are entirely obscured by the quadratojugal horn. In contrast, *Nodocephalosaurus kirtlandensis* (SMP VP-900) and a single specimen of *Euoplocephalus tutus* (AMNH FARB 5337) possess a condition in which the quadrates extend ventrally below the quadratojugal horns, possibly caused by breakage or deformation of the quadrate shaft. Despite being rotated anteriorly due to crushing, the quadrates, the immediately surrounding basicranial elements (pterygoids, exoccipitals) and the quadratojugal horns appear to be anatomically intact. Sutural contacts between the quadrate-pterygoid, pterygoid-basipterygoid, quadrate-exoccipital, and contacts between the quadrate-quadratojugal horns show no signs of breakage or offset. In *Akainacephalus johnsoni* the quadrate condyles are largely eroded away and are therefore anatomically uninformative.

#### Predentary

Most of the predentary is preserved, but the left lateral-most side is broken away as is the posterior-most portion along the right side that articulates with the anterior region of the dentary (Figs. 9I–9L). It is an unpaired, dorsoventrally depressed, and anteroposteriorly broad element that forms the mandibular counterpart to the premaxillary rostrum (Vickaryous, Maryańska & Weishampel, 2004) and articulates with the mandibular symphysis.

Very few ankylosaurid specimens preserve the predentary, and it has been primarily described in *Pinacosaurus grangeri* (Hill, Witmer & Norell, 2003), *M. ramachandrani* (INBR 21004), *Tarchia teresae* (Penkalski & Tumanova, 2017), and *Saichania chulsanensis* (Maryańska, 1977). In *Akainacephalus johnsoni*, the predentary is transversely almost straight, a condition observed in other ankylosaurid dinosaurs (e.g., *M. ramachandrani* [INBR 21004], and *P. grangeri* [MPC 100/1014]). The posterior-most portion of the right lateral side of the predentary is missing, but still preserves the reminiscence of a crescent-shaped morphology of the predentary in dorsal view (Figs. 9K–9L). Most of the left lateral margin is broken away. The external surface is irregular, highly rugose, and heavily ornamented with shallow and short vertical fovea and deeply pitted nutrient foramina. Its ventral midline is characterized by a distinct, ventrally oriented, sagittal protuberance (*tuberculum predentale*). Posteriorly, the predentary has a smooth surface texture.

#### Dentaries

The dentary constitutes the largest portion of the mandible and is morphologically similar to the same element in *Saichania chulsanensis* (no dentary is known for *Nodocephalosaurus*). In *Euoplocephalus tutus* (UALVP 31, Arbour & Currie, 2013a), and *Ankylosaurus magniventris* (Carpenter, 2004), the dentaries are dorsoventrally shallower. The dentary contacts the angular ventrally and the surangular posteriorly in lateral view. The dorsal surface of the right dentary is nearly entirely eroded away, preserving no alveoli and overall providing little anatomical information for this element (Figs. 9E and 9H). The left dentary is well-preserved and posseses a tall, anterodorsally convex alveolar border which is medially inset from the lateral margin of the jaw. This differs from other ankylosaurids such as *Zuul crurivastator* (Arbour & Evans, 2017), *Euoplocephalus tutus* (Vickaryous & Russell, 2003), *Ankylosaurus magniventris* (Carpenter, 2004), *M. ramachandrani* (INBR 21004) and *Tarchia teresae* (Penkalski & Tumanova, 2017), in which the dentaries are dorsoventrally shallower. The anterior margin of the alveolar border curves steeply ventrally, forming a deep concave opening between the alveolar border and mandibular symphysis. The alveolar border forms a sinuous tooth row in dorsal view (Figs. 9C and 9G). At least sixteen alveolar cavities are present but the anterior- and posterior-most alveoli have been eroded away, thus the total number of alveoli remains unknown. Most of the mandibular symphyses are missing, but a small remnant of the predentary sulcus (= predentary groove in Carpenter, 2001) is present on the lateral side of the left dentary and is positioned directly anterior to the alveolar cavities (Fig. 9A). A small keel runs longitudinally along the lateral surface of the mandibular symphysis and turns lateroventrally towards its anterior margin, slightly rotating the axis of this portion of the dentary. In medial view, the anterior-most portion of the alveolar border and mandibular symphysis gently curve anterolaterally. Medially, the dentary contacts the splenial and the coronoid, and a small portion of the Meckelian groove is visible. In lateral view, the anterior half of the dentary is dorsoventrally robust and laterally somewhat concave (Figs. 9A and 9E).

#### Splenials

The splenial is an anteroposterior, long, and thin bone that is positioned along the ventromedial surface of the mandible. It is ventrally convex and bordered by the mandibular caputegulum (Figs. 9A and 9E). In ventral view, the splenial has an undulating shape, medially convex along the anterior half and becoming medially concave along its posterior terminus where it contacts the prearticular. This morphology differs greatly from *M. ramachandrani* (INBR 21004) and *Pinacosaurus grangeri* (Hill, Witmer & Norell, 2003), which have splenials that are strictly concave medially, similar to *Ankylosaurus magniventris* (Carpenter, 2004) and *Euoplocephalus tutus* (Vickaryous & Russell, 2003). In their general description of ankylosaurid mandibles, Vickaryous, Maryańska & Weishampel (2004) mention that the splenial has a lengthy contact with the angular, but this contact is not visible in *Akainacephalus johnsoni* even though the region is clearly visible (i.e., not obscured by the mandibular caputegulum). A subtle ridge is present along the posterodorsal region of the splenial, cradling the prearticular, and appears to be the splenial–prearticular contact. This contact is anterodorsally inclined and curves sharply upward along its anterior margin, where it dorsally terminates at the ventral border of the coronoid, forming a triple contact between the prearticular, coronoid, and splenial. A large intermandibular foramen (*foramen intermandibulare caudale*) pierces the splenial. Vickaryous, Maryańska & Weishampel (2004) report that the intermandibular foramen is positioned ventral to the posterior-most tooth in *Euoplocephalus tutus*. Although the posterior margin of the alveoli are broken in *Akainacephalus johnsoni*, the location of the intermandibular foramen appears to be consistent with this description. This contrasts with some ankylosaurids such as *M. ramachandrani* (INBR 21004), where this foramen is located posterior to the posterior-most alveolus. The anterior margin of the splenial—dentary contact is not visible and the presence of a Meckelian groove can only be inferred. This area is damaged in both mandibulae, but a longitudinal furrow is present that might be part of the Meckelian groove.

#### Surangulars

Together with the posterior margin of the dentary, the surangular contributes to the tallest portion of the mandible, forming the coronoid process that terminates in a broadly triangular apex (Figs. 9A, 9B, 9E and 9F). Compared to other ankylosaurid taxa (i.e., *Tarchia teresae* (Penkalski & Tumanova, 2017), *Saichania chulsanensis* (Maryańska, 1977), *M. ramachandrani* [INBR 21004], *Euoplocephalus tutus* (Vickaryous & Russell, 2003), and *Ankylosaurus magniventris* (Carpenter, 2004)) in which the coronoid process only projects slightly more dorsally than the rest of the dentary, they extend much further dorsally in *Akainacephalus johnsoni*. Its posterior margin is convex and descends down where it curves medially and contacts with the prearticular and articular. Medially, the surangular surrounds a large and deep mandibular fossa, situated between the prearticular, coronoid, and angular. The anterior suture with the dentary and the medial sutures with the coronoid and splenial are not visible, but this condition is not observed in all ankylosaurids. In *M. ramachandrani* (INBR 21004), for example, the surangular—dentary contact is characterized by a distinct saw-tooth suture pattern.

#### Coronoids

The coronoid is difficult to identify and rarely observed in ankylosaurian dinosaurs (Vickaryous, Maryańska & Weishampel, 2004). In *Akainacephalus johnsoni*, the coronoid is present on both mandibles but is best preserved on the left mandible (Fig. 9B). It forms a dorsally concave sheet of bone that contacts the medioposterior surface of the alveolar border, the anteromedial margin of the surangular, the anterodorsal border of the prearticular, and the posterodorsal border of the splenial.

#### Prearticulars

The prearticular is a subrectangular element that contacts the surangular medially, and the anteroventral margin contacts the splenial (Figs. 9A, 9B, 9E and 9F). Together with the surangular, the prearticular contributes to the retroarticular process. In dorsal view, the posterior half of the prearticular is the widest and gradually tapers to form a medially concave surface that joins with the splenial and the coronoid. Its dorsal surface is nearly horizontal but the posterior half inclines posteroventrally to form a shallow, dorsally concave surface.

## Axial Skeleton

### Cervical vertebrae

**Figure 10:**
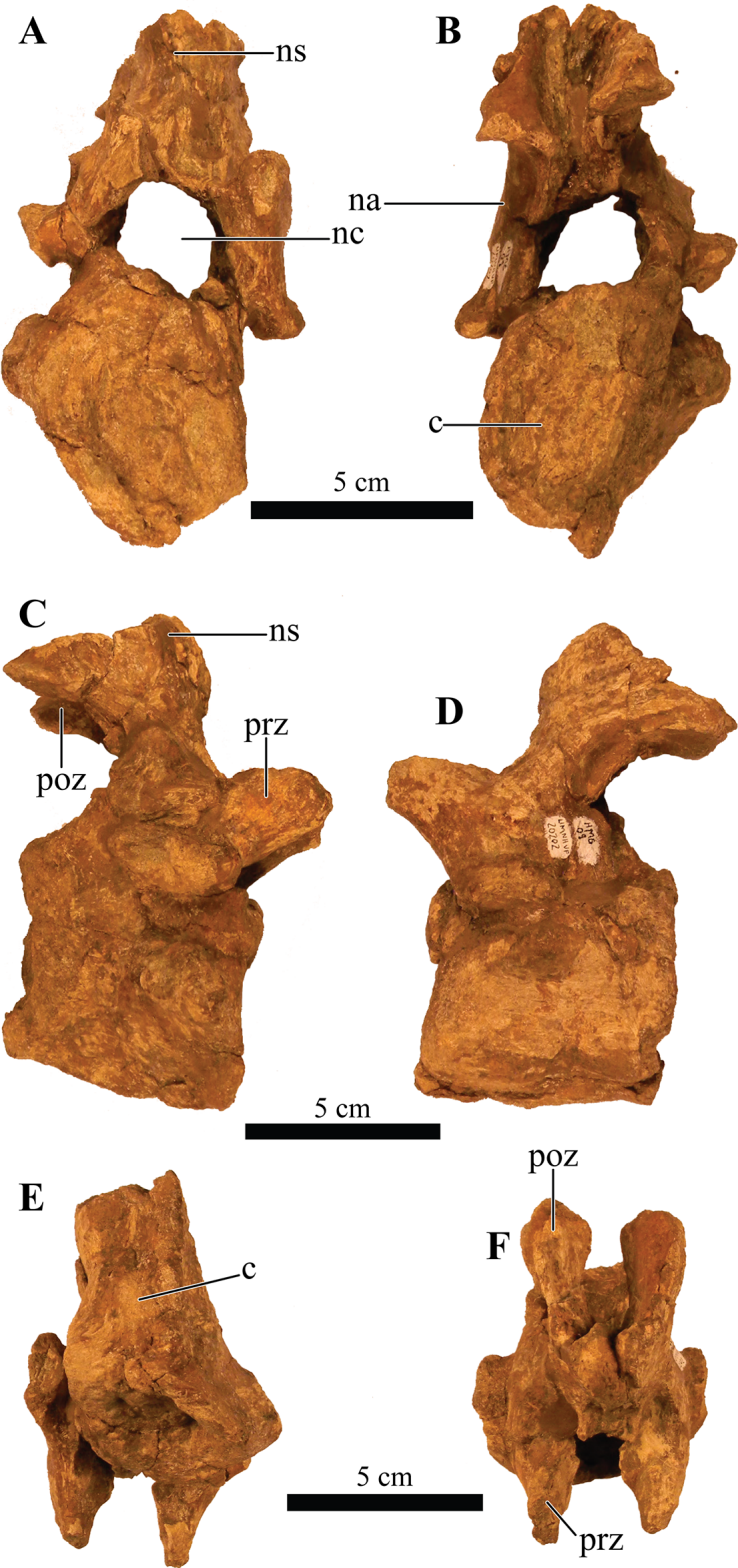
Cervical vertebra. Photographs of the cervical vertebra of *Akainacephalus johnsoni* (UMNH VP 20202) in (A), anterior; (B), posterior; (C) right lateral; (D), left lateral; (E), ventral; and (F), dorsal views. Study sites: c, centrum; na, neural arch; nc, neural canal; ns, neural spine; poz, postzygopophyses; prz, prezygopophyses.

A total of two partial cervical vertebrae are preserved with the skeleton; an isolated neural arch, and a nearly complete cervical vertebra which exhibits postdepositional lateral compression in addition to breakage that removed the posterior right half of the articular surface of the centrum (Figs. 10B and 10D). The cervical vertebra is morphologically similar to those observed in other ankylosaurids (e.g, *Euoplocephalus tutus* (Vickaryous, Maryańska & Weishampel (2004); Arbour & Currie, 2013a), *Ankylosaurus magniventris* (Brown, 1908; Carpenter, 2004), and *Scolosaurus cutleri* (Penkalski & Blows, 2013)). The vertebra is relatively compact and contains a subrounded, anteroposteriorly longer than dorsoventrally tall and mediolaterally wide, spool-shaped centrum, suggesting it is a posterior cervical vertebra, similar to the sixth cervical of *Ankylosaurus magniventris* (Brown, 1908; Carpenter, 2004; Vickaryous, Maryańska & Weishampel, 2004). Articular facets of the centrum are amphicoelous, and the posterior face is dorsally elevated compared to the anterior face. A distinct, longitudinal keel is present on the ventral side of the centrum. The parapophyses are positioned on the anterodorsal area of the centrum and the diapophyses are located anterolaterally along the neural arch, similar to *Euoplocephalus tutus* (Arbour & Currie, 2013a) and *Ankylosaurus magniventris* (Carpenter, 2004). The neural canal is large and round and is roofed by a short and broad neural spine from which the dorsal-most portion is missing. The prezygopophyses are positioned at the base of the neural canal, forming stout and anterodorsally projection elements which are broken terminally, preserving no articular facets. The dorsoposterior surface of the neural spine accommodates the postzygopophyses. The articular facets of the postzygopophyses are nearly horizontal with a ventral orientation.

### Dorsal vertebrae

**Figure 11:**
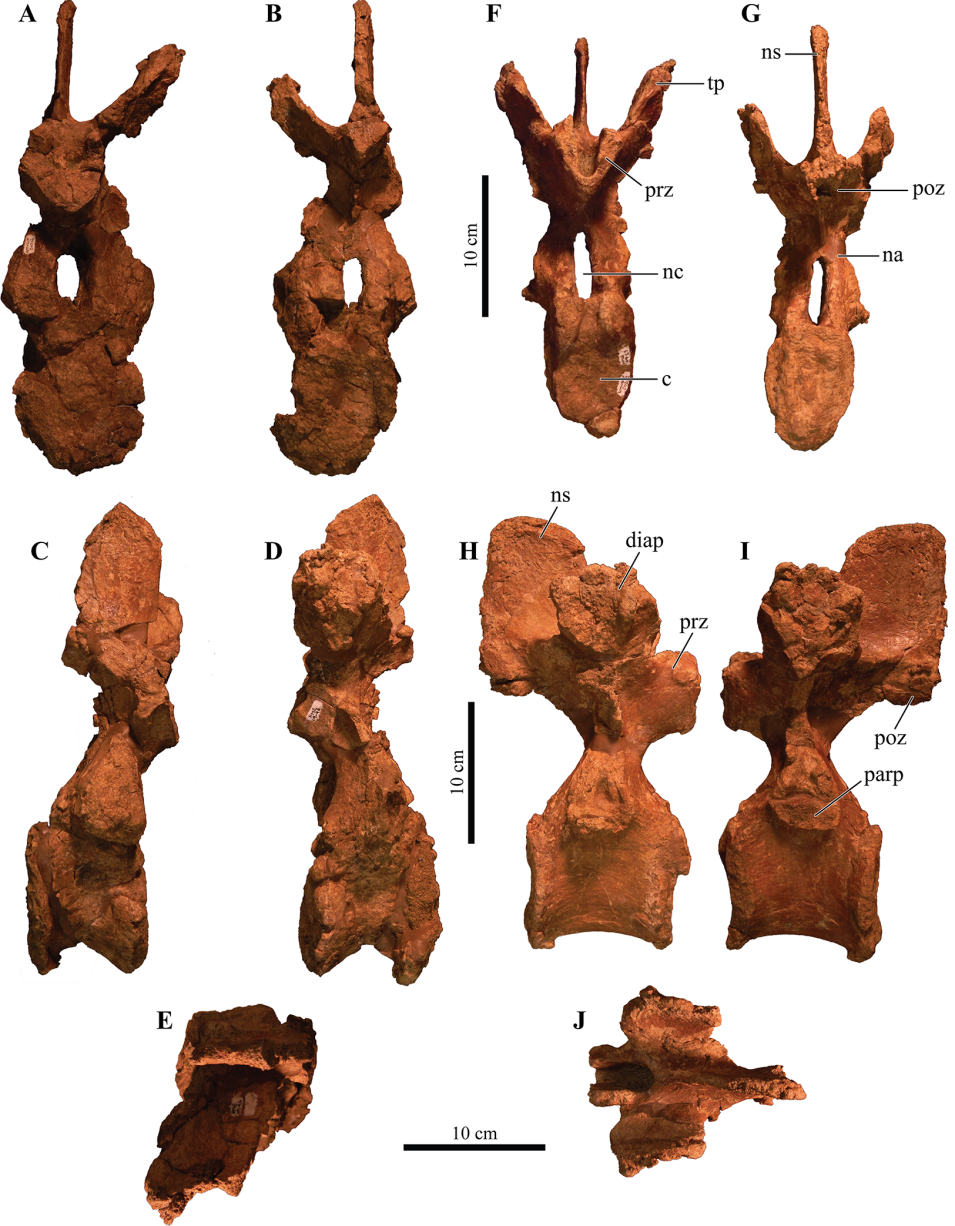
Free anterior dorsal vertebrae. Photographs of the two anterior dorsal vertebrae of *Akainacephalus johnsoni* (UMNH VP 20202) in (A) and (F), anterior; (B) and (G), posterior; (C) and (H), right lateral; (D) and (I), left lateral; (E) and (J), dorsal views. Ribs are broken away in both vertebrae but articulation with the diapophyses and parapophyses are clearly visible. Study sites: c, centrum; diap, diapophysis; na, neural arch; nc, neural canal; ns, neural spine; parp, parapophysis; poz, postzygopophyses; prz, prezygopophyses; tp, transverse process.

A total of four dorsal vertebrae are preserved. Two anterior dorsal vertebrae display heavy postdepositional deformation, lateral compression, and lack the fused ribs (Fig. 11). The centra are spool-shaped, laterally constricted, and anteroposteriorly longer than dorsoventrally tall. Because the vertebrae are laterally compressed, the centra appear dorsoventrally oblong in anterior and posterior views. A single dorsal vertebra preserves the notochordal protuberance on the posterior facet of the centrum. A small, recurving, hook-like projection is present ventrally on the posterior surface of the centrum (Figs. 11H–11I) contrary to other ankylosaurids (e.g., *Talarurus plicatospineus* (Maleev, 1956); *Ankylosaurus magniventris* (Carpenter, 2004); AMNH FARB 5337 (Arbour & Currie, 2013a); cf. *Pinacosaurus* (MPC 100/1305 [=*Saichania chulsanensis*, Carpenter et al., 2011; Arbour & Currie, 2013b)). This projection is anteriorly followed by the presence of a shallow and longitudinal keel (= ventral medial ridge in Carpenter, 2004) that extends along the ventral length of the centrum. The articular surface on the anterior facet of the centra is slightly elevated with respect to the posterior facet, which is consistent in ankylosaurian dinosaurs. The neural canals are oblong, with the long axis running dorsoventrally in both vertebrae; this is a condition common in the anterior dorsal vertebrae of ankylosaurids (Vickaryous, Maryańska & Weishampel, 2004). No ribs are preserved with the dorsal vertebrae, but the parapophyses are present at the junction of the neurocentral suture and the diapophyses are located on the distal ends of the transverse processes. The prezygopophyses are positioned directly above the neural canal, merge medially, and form a rigid, single structure with a distinct V-shape in which the articular surfaces for the postzygopophyses face mediodorsally. Some of the prezygopophyses do not extend beyond the anterior portion of the centrum. The postzygopophyses form a laterally thickened structure, at the ventral continuation of the neural spine, and the articular surfaces face ventrolaterally. Two steeply dorsolaterally oriented transverse processes and a anteroposteriorly broad neural spine form the roof of the neural canal.

**Figure 12:**
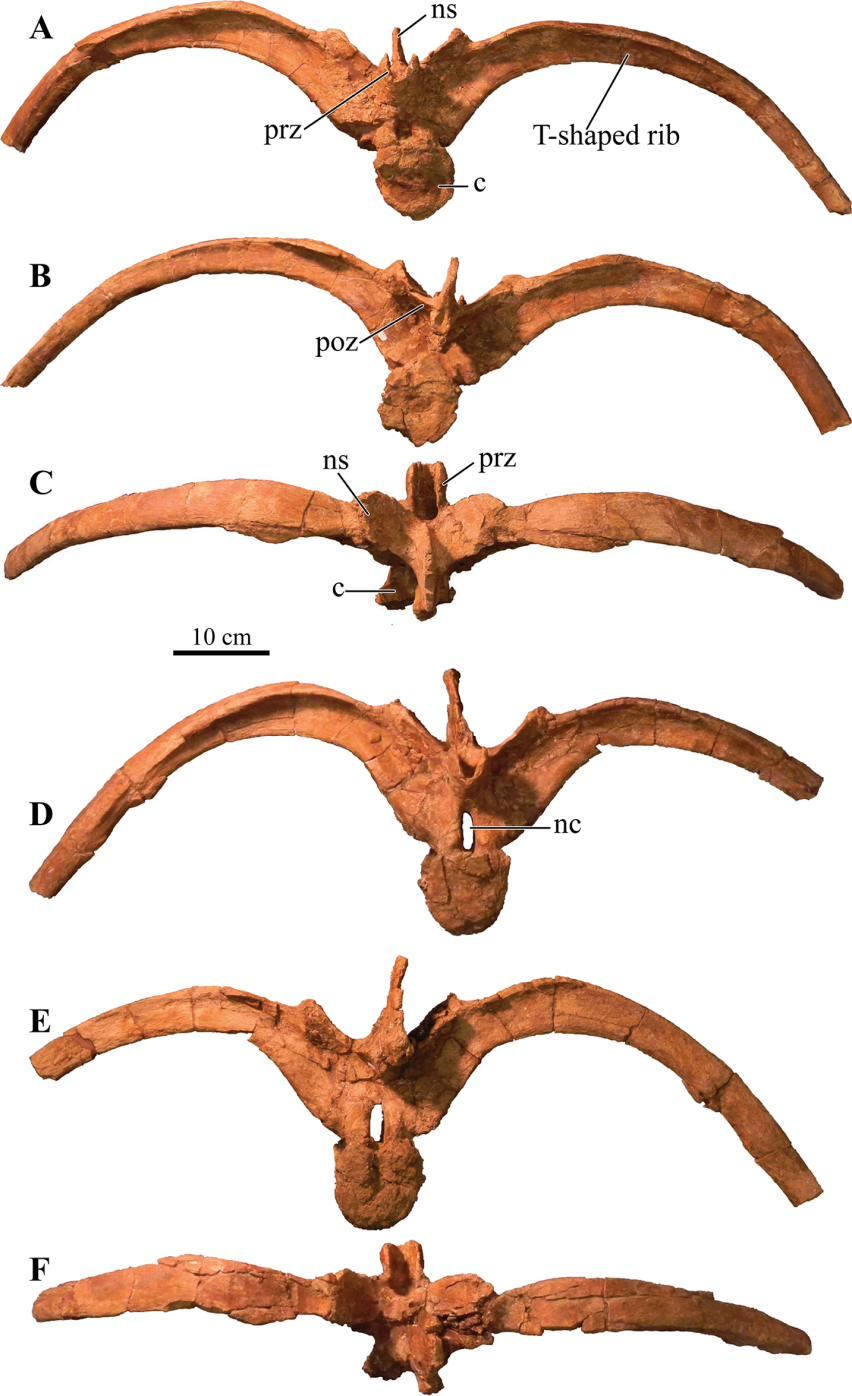
Free mid-dorsal vertebrae. Photographs of two mid-dorsal vertebrae with fused ribs of *Akainacephalus johnsoni* (UMNH VP 20202) in (A) and (D), anterior; (B) and (E), posterior; (C) and (F), dorsal views. Study sites: c, centrum; nc, neural canal; ns, neural spine; poz, postzygopophyses; prz, prezygopophyses.

The other two dorsal vertebrae preserve large portions of the fused ribs (Fig. 12). The morphology of the centra, neural canals, and prezygopophyses, suggests that they belong to the mid-dorsal series. Laterally, the vertebrae show minor deformation. In anterior and posterior view, both centra are severely broken and weathered but possess a substantially rounder morphology compared to the anterior dorsal vertebrae in *Akainacephalus johnsoni*. They are somewhat ovoid compared to the round dorsal vertebrae observed in *Ankylosaurus magniventris* (Carpenter, 2004). Although the neural canal becomes significantly rounder in the posterior dorsal vertebrae in ankylosaurids (e.g., *Ankylosaurus magniventris* (Carpenter, 2004), *Euoplocephalus tutus* (Vickaryous, Maryańska & Weishampel, 2004; Arbour & Currie, 2013a), and *Pinacosaurus grangeri* (Hill, Witmer & Norell, 2003)), it retains an ovoid morphology with a vertical long axis in *Akainacephalus johnsoni*, likely caused through postdepositional mediolateral deformation. Similar to the anterior dorsal vertebrae, the prezygopophyses are obliquely oriented dorsally and merge medially to form a V, with the articulation surfaces oriented dorsomedially. The postzygopophyses are situated directly below the posterior extension of the neural spine and articulation surfaces are oriented ventrolaterally. The fused ribs are T-shaped in cross section proximal to their articulation with the transverse processes of the vertebrae, but this morphology becomes distally more ovoid and the distal portions are broken away. The ribs extend from the vertebrae in a curved manner at approximately 45° ventrolaterally. The diapophyses are located on the ventral surface of the transverse processes and form the surface upon which the capitulum fuses, whereas the tubercle fuses on the parapophyses, which are located on the lateral side of the vertebra, between the centrum and neural arch. A dorsoventrally tall and anteroposteriorly broad neural spine is present on all the dorsal vertebrae, but is shorter in comparison to the neural spines on the anterior dorsal vertebrae. The anterior end of the neural spine forms a steeply anteroventral descending margin, whereas the posterior margin is vertical. Measurements for the dorsal vertebrae are summarized in Supplemental Material S4.

### Sacral vertebrae

**Figure 13:**
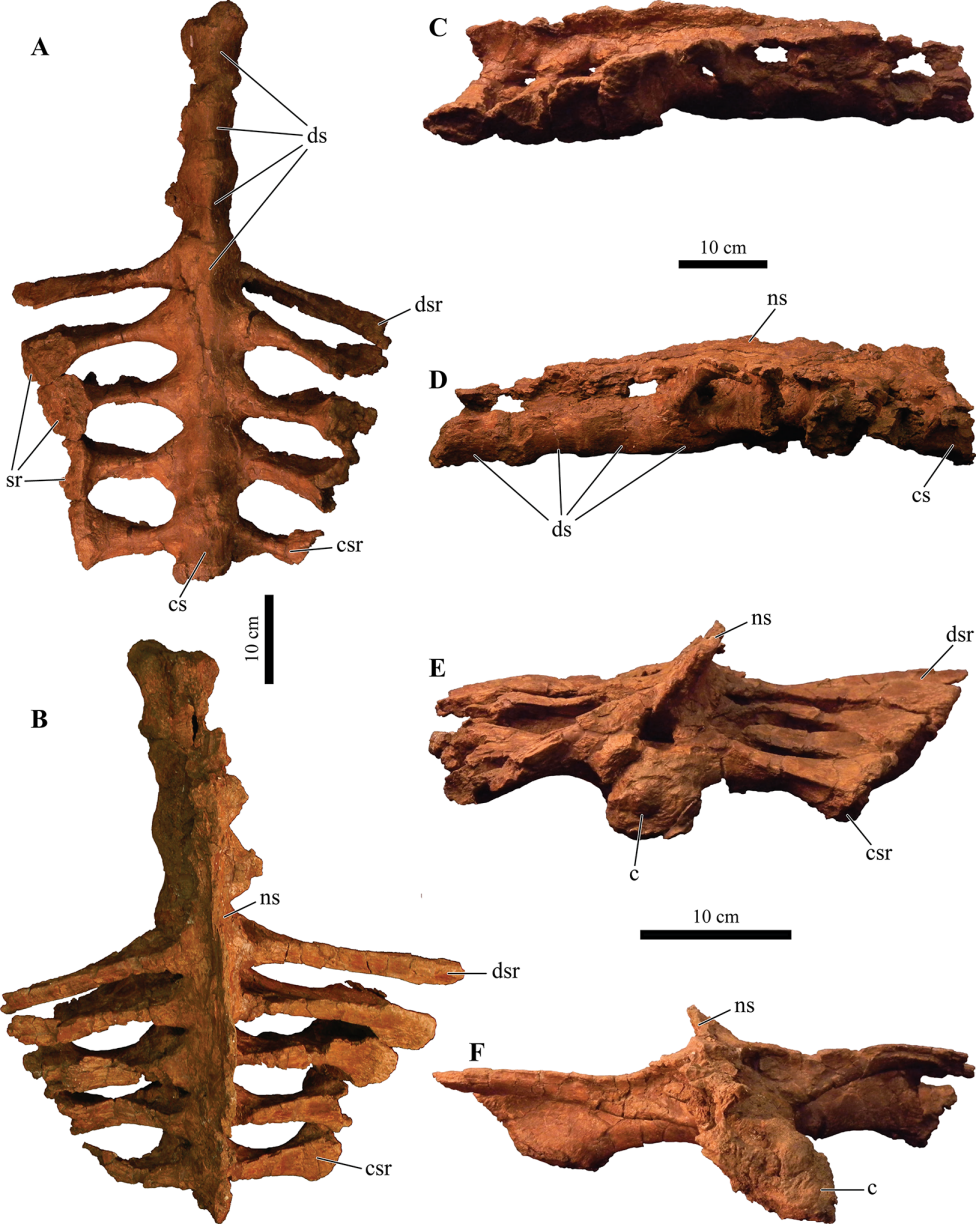
Sacrum, presacral, and caudosacral vertebrae. Photographs of the sacrum, presacral rod, and caudosacral vertebrae of *Akainacephalus johnsoni* (UMNH VP 20202) in (A), ventral; (B), dorsal; (C), right lateral; (D), left lateral; (E), posterior; and (F), anterior views. Study sites: c, centrum; cs, caudosacral vertebra; csr, caudosacral ribs; ds, dorsosacral vertebrae; dsr, dorsosacral ribs; ns, neural spine; sr, sacral ribs.

The well-preserved, nearly complete synsacrum contains eight co-ossified vertebrae, including dorsosacral, sacral, and caudosacral vertebrae (Fig. 13). The synsacrum experienced slight postdepositional dextral deformation (Figs. 13E and 13F). A total of four dorsosacral, three sacral vertebrae (a first primordial, inserted, and second primordial sacral (cf. Nesbitt, 2011: fig. 29)), and a single caudosacral vertebra make up the synsacrum. All vertebrae are fused along the centra and the neural spines, forming an immobile element. Sutural contacts are nearly obliterated between individual vertebrae of *Akainacephalus johnsoni*, unlike the synsacrum of some ankylosaurid specimens such as *Euoplocephalus tutus* (AMHH FARB 5409 (Coombs, 1971)), in which the sutures are clearly visible, which could be an ontogentic feature. Individual vertebrae are longer than wide, spool-shaped, and display laterally compressed, concave surfaces (Figs. 13A and 13B). The neural arches are tall and narrow on the dorsosacral vertebrae but are short and wider on the caudosacral vertebra. Neural canals on the sacral vertebrae are difficult to observe, because they are obscured by matrix and slightly dorsoventrally compressed. The neural spines are broken on the dorsosacral vertebra but overall are short and fused into a large sheet that runs across the dorsal surface of the sacral vertebrae. The three sacral and single caudosacral vertebrae are morphologically almost identical; however, the caudosacral vertebra possesses a well-defined articular pleurofossal facet for the first free caudal vertebra. In addition, a prominent notochordal protuberance is present in the middle of the posterior face of the centrum, indicating this is the terminal vertebra in the synsacrum.

The ribs and transverse processes on dorsosacral vertebrae 1–3 are broken away from their respective centra (Figs. 13A–13D). The remaining vertebrae contain short, horizontally oriented ribs that are fused to the centrum and transverse processes and typically decrease in length with each successive posterior vertebra. Distally, the ribs expand anteroposteriorly and dorsoventrally to form broad, ovoid contact surfaces that articulate with the medial surface of the ilium (Figs. 13A and 13C).

Complete ankylosaurid synsacra are only known for a few taxa and display both ontogenetic and taxonomic variation seen in juvenile and adult specimens of *Pinacosaurus* (Maleev, 1954), cf. *Pinacosaurus* (MPC 100/1305 (=*Saichania chulsanensis*, Carpenter et al., 2011; Arbour & Currie, 2013b)), *Talarurus plicatospineus* (Maleev, 1952, 1956), *Euoplocephalus tutus* (Coombs, 1979; Arbour & Currie, 2013a), but depend heavily on the preserved condition of the specimen. Distinguishing between dorsosacral and sacral vertebrae can be a challenging undertaking. In their taxonomic revision of *Euoplocephalus tutus*, Arbour & Currie (2013a) distinguish between the dorsosacral and sacral ribs based on their cross-sectional morphology; they argue that the dorsosacral ribs express the typical T-shaped morphology in cross section, whereas the sacral ribs do not express any dorsal expansion and are ellipsoid in cross section, and that the sacral ribs exclusively bracket the acetabulum. Carpenter et al. (2011) provided a more detailed anatomical description of the morphology and position of the dorsosacral and sacral ribs but ignored the specific location of the sacral ribs with respect to the acetabulum. Nesbitt (2011) discusses how the sacral ribs articulate with the ilium, which subsequently allows for the identification of the primordial and inserted sacral vertebrae.

In this section, the criteria provided by Carpenter et al. (2011), Nesbitt (2011), and Arbour & Currie (2013a) are combined in order to differentiate between the dorsosacral and sacral verterbrae of *Akainacephalus johnsoni*. The sacral ribs are directed nearly horizontal and expand distally to form the broad surface that articulates with the medial face of the ilium. These features are present on all synsacral vertebrae with the exception of dorsosacral vertebrae 1–3 (Fig. 13). The ribs of dorsosacral 4 are long and possess a narrow shaft. Ventrally, the surface of the proximal half is narrow, whereas the dorsal surface expands horizontally, forming a well-defined T-shaped morphology. Distally, the ribs flatten dorsoventrally, losing the T-shape morphology, and the distal ends articulate with the medial face of the ilium but do not form a broad contact. Medially, the dorsosacral ribs articulate with the dorsolateral surface of the centrum and the ventral surface of the tranverse processes. In contrast, the sacral and caudosacral ribs fuse to the ventral side of both the centra and transverse processes, forming dorsoventrally tall and robust ribs (Fig. 13). The articular surface on the sacral rib of sacral vertebra one (= primordial sacral one of Nesbitt, 2011) is dorsoventrally ovoid, anteroposteriorly narrow, and articulates with the preacetabular process of the ilium. The articular surface of the sacral rib on sacral vertebra three (= primordial sacral two of Nesbitt, 2011) is larger than in sacral rib one and is teardrop shaped; it articulates with the postacetabular process of the ilium. Situated between the primordial sacral vertebrae is the inserted sacral vertebra (sensu Nesbitt, 2011). Its sacral rib possesses a dorsoventrally tall and broad articular surface that contacts the mediodorsal portion of the acetabulum. The dorsal surfaces of the sacral ribs expand horizontally but are less developed in this respect compared to the ribs on the dorsosacral vertebrae, resulting in a much less pronounced T-shaped cross-sectional morphology. This differs from the sacral ribs of cf. *Pinacosaurus* (MPC 100/1305 (=*Saichania chulsanensis*, Carpenter et al., 2011; Arbour & Currie, 2013b)) which represent an I-beam in cross section (Carpenter et al., 2011). The sacral and caudosacral ribs are much shorter and decrease in length with each posteriorly successive vertebra.

Ventrally, the synsacral vertebrae lack the presence of a longitudinal groove, a condition commonly observed in other ankylosaurids (Carpenter et al., 2011) (e.g., *Euoplocephalus tutus* [AMNH 5409], MPC 100/1305, and *Anodontosaurus lambei* (Arbour & Currie, 2013a)). Longitudinal grooves along the ventral surface of the sacral vertebrae have been observed in most nonankylosaurid thyreophoran dinosaurs, e.g., *Gastonia burgei*, *Edmontonia longiceps*, *Nodosaurus textilis*, and *Cedarpelta bilbeyhallorum* (Kirkland, 1998; Carpenter et al., 2008; Loewen & Kirkland, 2013), but they have been reported to be absent in the nodosaurid *Silvisaurus condrayi* (Carpenter & Kirkland, 1998). The sacral vertebrae are bowed to form a shallow, ventrally concave arch.

### Caudal vertebrae

**Figure 14:**
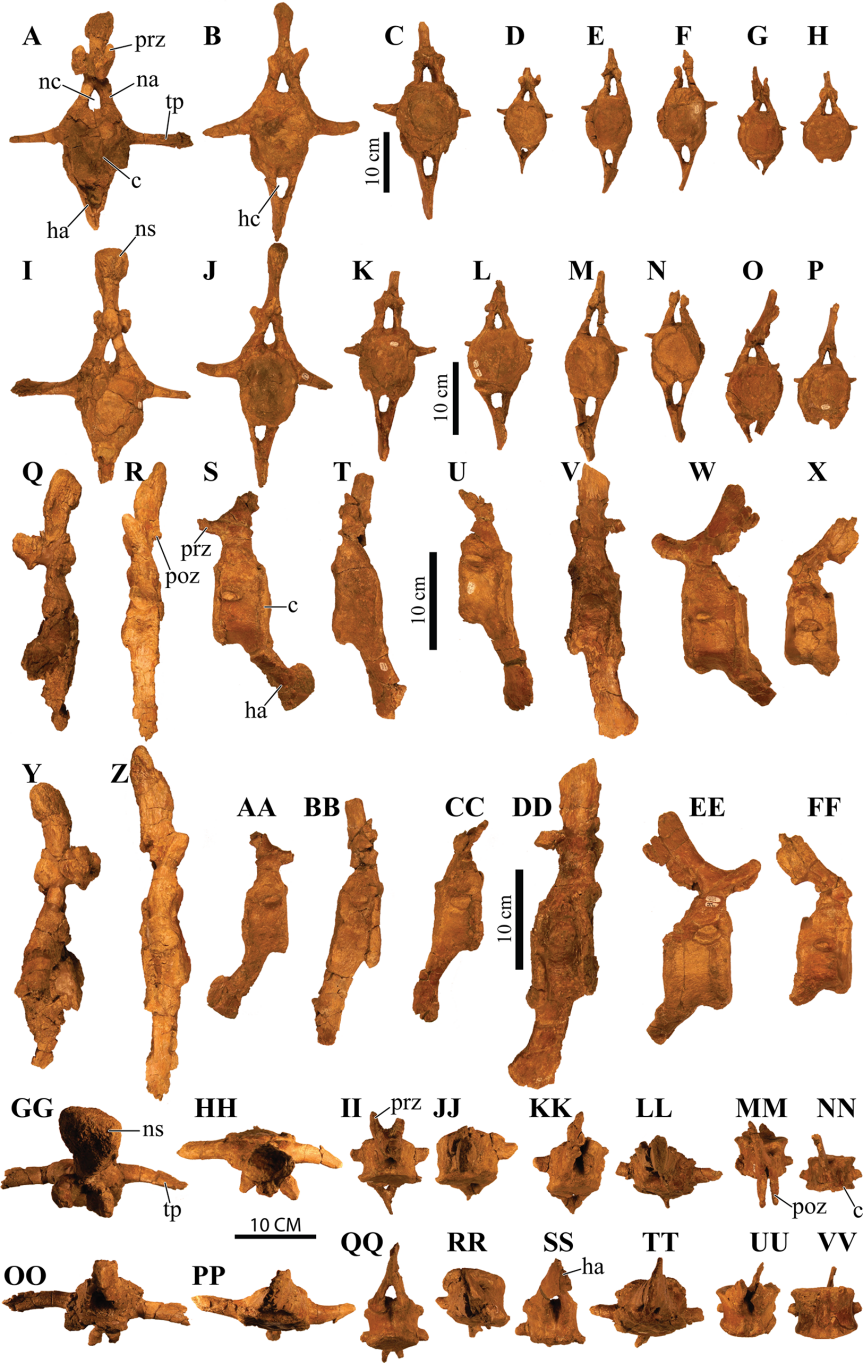
Anterior caudal vertebrae. Photographs of eight anterior caudal vertebrae of *Akainacephalus johnsoni* (UMNH VP 20202) in (A–H), anterior; (I–P), posterior; (Q–X), left lateral; (Y–FF), right lateral; (GG–NN), dorsal; and (OO–VV), ventral views. Study sites: c, centrum; ha, haemal arch; hc, haemal canal; na, neural arch; nc, neural canal; ns, neural spine; poz, postzygopophyses; prz, prezygopophyses; tp, transverse process.

A total of eight anterior caudal vertebrae are preserved and form the mobile portion of the tail (Fig. 14). All caudal vertebrae experienced crushing, breakage, and deformation to some extent, so none are fully complete. All vertebrae preserve dorsoventrally tall haemal arches, which are tightly fused onto the ventral part of the centrum, and possess a distinctly flattened keel that expands anteroposteriorly, forming a short but tall boot (Figs. 14S, 14U, 14V, 14AA, 14CC and 14DD). Centra are laterally constricted and preserve spool-shaped, slightly amphicoelous, anterior and posterior articular surfaces, similar to those observed in *Talarurus plicatospineus* (Maleev, 1956), *Oohkotokia horneri* (MOR 433), SMP VP-1149, and SMP VP-1743, with the latter two previously assigned to *Nodocephalosaurus kirtlandensis* (Sullivan & Fowler, 2006). In *Ankylosaurus magniventris* (AMNH FARB 5895) and *Anodontosaurus lambei* (CMN 8530), the anterior and posterior faces of the centra are more deeply excavated, resulting in more pronounced amphicoelous conditions. The neural spines are tall on all caudal vertebrae and, including the neural arches, account for nearly 50% of the total vertebral height.

The first two anterior caudal vertebrae are the largest and most complete vertebrae preserved in the free caudal series (Figs. 14A, 14B, 14I, 14J, 14Q, 14R, 14Y and 14Z) and are morphologically most similar to *Ankylosaurus magniventris* (Carpenter, 2004) and *Euoplocephalus tutus* (Arbour & Currie, 2013a). The centra are ellipsoid in anterior and posterior view, a condition that is consistent across all eight proximal caudal vertebrae; however, the central facets become more round in the more distal caudals of the proximal series. Additionally, the anteroposterior length of these caudals is strongly abbreviated (Figs. 14Q, 14R, 14Y and 14Z), but this becomes less prominent in the remaining caudal vertebrae. Their centra become anteroposteriorly longer further down the series. The transverse processes in the first and second caudal vertebrae are long, dorsally concave, and ventrally convex surfaces with a nearly horizontal projection in anterior and posterior view (Figs. 14A, 14B, 14I and 14J) similar to *Ankylosaurus magniventris* (Carpenter, 2004). This condition differs from *Talarurus plicatospineus* (Maleev, 1956), *Dyoplosaurus acutosquameus* (ROM 784), and *O. horneri* (MOR 433), in which the transverse processes are directed obliquely ventrally in anterior and posterior view, and directed obliquely anteriorly in lateral view. The transverse processes curve slightly anteriorly in dorsal view. The pre- and postzygopophyses are positioned above the neural arch but are mostly broken or missing. The prezygopophyses are short and robust in these vertebrae compared to the smaller but anteroposteriorly longer prezygopophyses in the remaining caudals of the series. The neural arches are tall and form the lateral border of a subtriangular to vertically oval neural canal, dorsally roofed by a tall neural spine. The neural spine comprises mediolaterally narrow shaft that strongly expands at its dorsal-most region. In *Ankylosaurus magniventris* (Carpenter, 2004) and *Euoplocephalus tutus* (Arbour & Currie, 2013a) this region is also expanded but to a much lesser extent. Anterior caudals three through six are morphologically similar to the first two caudal vertebrae, but the centra are anteroposteriorly less abbreviated. Most of the neural spines and the majority of the transverse processes are postdepositionally broken away. The articular facets of the centra in the third through sixth caudal vertebrae are round in anterior and posterior view and the anterior facet is dorsally elevated compared to the posterior facet.

Anterior caudals seven and eight are the smallest of the preserved caudal series and the interecentral articular surfaces are nearly round (Figs. 14G, 14H, 14O and 14P). Although they represent the smallest of the free caudal series, the anteroposterior length of their centra is relatively larger compared to the first six caudal vertebrae (Figs. 14Q–14FF). Similar to the other six anterior caudal and the two dorsal vertebrae, the anterior facets of the centrum are dorsally elevated relative to the posterior facets. Similar to caudals three through six, the transverse processes form reduced, horizontally directed projections with their distal-most ends eroded away. The transverse processes are positioned on the dorsal half of the centrum. Caudal vertebrae seven and eight in *Akainacephalus johnsoni* differ strongly from SMP VP-1149 and SMP VP-1743, which preserve more robust, and ventrolaterally projecting transverse processes. The transverse processes on the distal vertebrae of the free caudal series in *Andodontosaurus lambei* appear to have been fully lost (Arbour & Currie, 2013a: fig. 9K–I). Only the proximal-most portions of the haemal arches are preserved and are firmly fused onto the ventral margin of the centrum. The neural canal is vertically oblong-subtriangular. This condition differs from SMP VP-1149 and SMP VP-1743, which possess round neural canals in the distal vertebrae of the proximal caudal series. The neural spines are dorsoventrally shorter and directed more obliquely posterior compared to the other free caudal vertebrae. Contrary to the mediolaterally expanded distal ends of the neural spines in the first six anterior caudals, the distal ends of the neural spines in caudals seven and eight are mediolaterally flat, similar to a distal anterior caudal vertebra preserved in *Anodontosaurus lambei* (Arbour & Currie, 2013a).

### Fused posterior caudal vertebrae (handle) and knob

#### Handle

**Figure 15:**
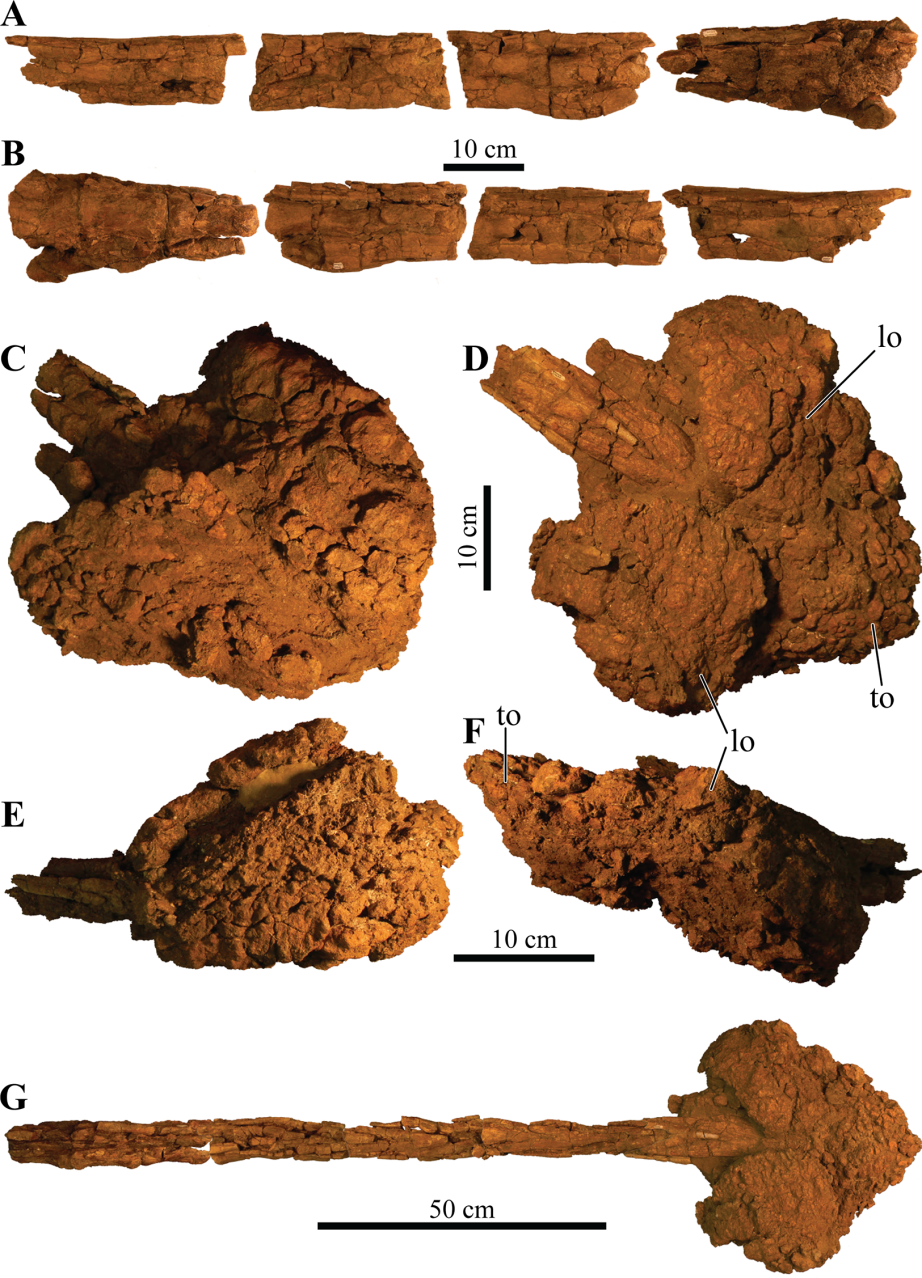
Fused caudal vertebrae (handle) and tail club knob. Photographs of distal caudal vertebrae (handle) and tail club of *Akainacephalus johnsoni* (UMNH VP 20202). Handle in (A), left lateral; and (B), right lateral view. Tail club in (C), dorsal; (D), ventral; (E), left lateral; and (F), right lateral views. Reconstructed handle and tail club in (G), dorsal view. Study sites: lo, lateral osteoderms; to, terminal osteoderms.

The well-preserved handle comprises eleven fused vertebrae (Figs. 15A and 15B) and terminates in a large tail club knob of two lateral osteoderms and what appears to be a single distal osteoderm (Figs. 15C, 15D, 15E, 15F and 15G). Although the overall morphology of the handle and tail club of *Akainacephalus johnsoni* differs from *Dyoplosaurus acutosquameus* (ROM 784), the number of fused caudal vertebrae is identical; this character varies among different ankylosaurid taxa and can be as many as seventeen, as has been observed in the handle of ZPAL MgD I/113 (previously referred to *Tarchia* cf. *gigantea* (Arbour et al., 2013)). The centra are spool-shaped with laterally-compressed concave surfaces. Each individual vertebra is nearly twice as long as wide and vertebrae are approximately equal in size, ranging between 98 and 105 mm in anteroposterior length, but the width and height of the centra decrease posteriorly across successive vertebrae. The handle is dorsally reinforced by posteriorly elongated, wedge-shaped prezygopophyses that extend across nearly 50% of the length of the adjacent vertebra and interlock with the shorter postzygopophyses (Fig. 15G). Chevrons are fused onto the ventral surface of the centra and dorsoventrally compressed so that they look like elongated rods in lateral view. In ventral view, the chevrons are posterior-oriented V-shaped structures that interlock with one another and reinforce the handle ventrally. Some ankylosaurids (e.g., *D. acutosquameus* [ROM 784], cf. *Pinacosaurus* [MPC 100/1305], ZPAL MgD I/113, and UMNH VP 19472) preserve a dense network of ossified tendons along the lateral sides or the handle, but these are not preserved in *Akainacephalus johnsoni*.

#### Knob

A large, wider than long, robust tail club knob is fused onto the two posterior-most caudal vertebrae and comprise three distinct osteoderms; two anteroposteriorly elongated lateral major osteoderms and what appears to represent a single, subtriangular terminal minor osteoderm (Figs. 15C and 15D), revealing no evidence of multiple minor osteoderms, but its poor preservation makes this uncertain. In dorsal and ventral view, the knob itself displays an overall subtriangular morphology with a broad and blunt apex, a condition uncommon in other Laramidian ankylosaurids in which the knobs are rounder (e.g., *Ankylosaurus magniventris* (Carpenter, 2004), CMN 349, TMP 1983.36.120, TMP 2001.42.9, and SMP VP-2074 (Burns & Sullivan, 2011b)) and more elongate (e.g., *Zuul crurivastator* (Arbour & Evans, 2017)). Because some of the lateral and dorsal areas are eroded, the original morphology was possibly rounder along the lateral margins. The tail club knob morphology of *Akainacephalus johnsoni* also differs from UMNH VP 19472, the North American *D. acutosquameus* (ROM 784) and some Asian taxa (e.g., cf. *Pinacosaurus* [MPC 100/1305], and ZPAL MgD I/113). In these taxa, the knobs are much smaller and the major osteoderms are transversely narrower. The tail club in *Akainacephalus johnsoni* has a posterodorsal inclination of 10–15° in lateral view (Figs. 15E and 15F). The medial bases of the major osteoderms that make up the tail club knob are deeply excavated and envelope the distal-most caudal vertebrae.

Both major osteoderms are anteroposteriorly longer than they are mediolaterally wide in dorsal and ventral views, similar to *Euoplocephalus tutus* (CMN 2251), *Ankylosaurus magniventris* (AMNH FARB 5214), and other specimens referred to Ankylosauridae such as USNM 10753, CMN 349, TMP 1983.36.120, UALVP 16247 (Arbour & Currie, 2013a), and SMP VP-2074. In dorsal view, the osteoderms are badly damaged but preserve and overall rounded morphology along the medial and lateral margins. The lateral margins are clearly rounded, convex, and differ from *Anodontosaurus lambei* (Arbour & Currie, 2013a), in which the lateral margins terminate in a distinct apex and curve dorsolaterally upward. In ventral view, the osteoderms have a semicircular morphology and the posterior halves extend further medially and contact each other, enclosing the ventral surface on the posterior end of the tail club and nearly obscuring all of the ventral portion of the minor osteoderm, forming a small semicircular wedge.

The posterior portion of the knob is far smaller compared to the anteriorly positioned lateral osteoderms, and forms the terminal part of the tail club. Dorsally, this area is transversely wider than it is long and has a diamond-shaped morphology, most similar to certain tail club knob specimens assigned to *Anodontosaurus lambei* (AMNH FARB 5216) and Ankylosauridae *indet*. (UALVP 16247). The posterior-most end of the knob forms a small apex. The surface texture of the osteoderms is deeply pitted, displaying a highly irregular and globular surface texture, possibly a result of extensive weathering. A similar condition is also observed in tail club knobs belonging to *Euoplocephalus tutus* (Burns & Sullivan, 2011b; Arbour & Currie, 2013a) and TMP 2001.42.19 (Arbour & Currie, 2013a) (= *Oohkotokia horneri* (Penkalski, 2014)). Isolated tail club knob remains (SMP VP-1632, SMP VP-1646, SMP VP-2074) from the Kirtland Formation of New Mexico display far smoother surface textures (Burns & Sullivan, 2011b). Because these specimens were not associated with cranial material, they could belong to either *Nodocephalosaurus kirtlandensis* (SMP VP-900), *Ziapelta sanjuanensis* (Arbour et al., 2014), or other undiscovered taxa.

### Dorsal ribs

**Figure 16:**
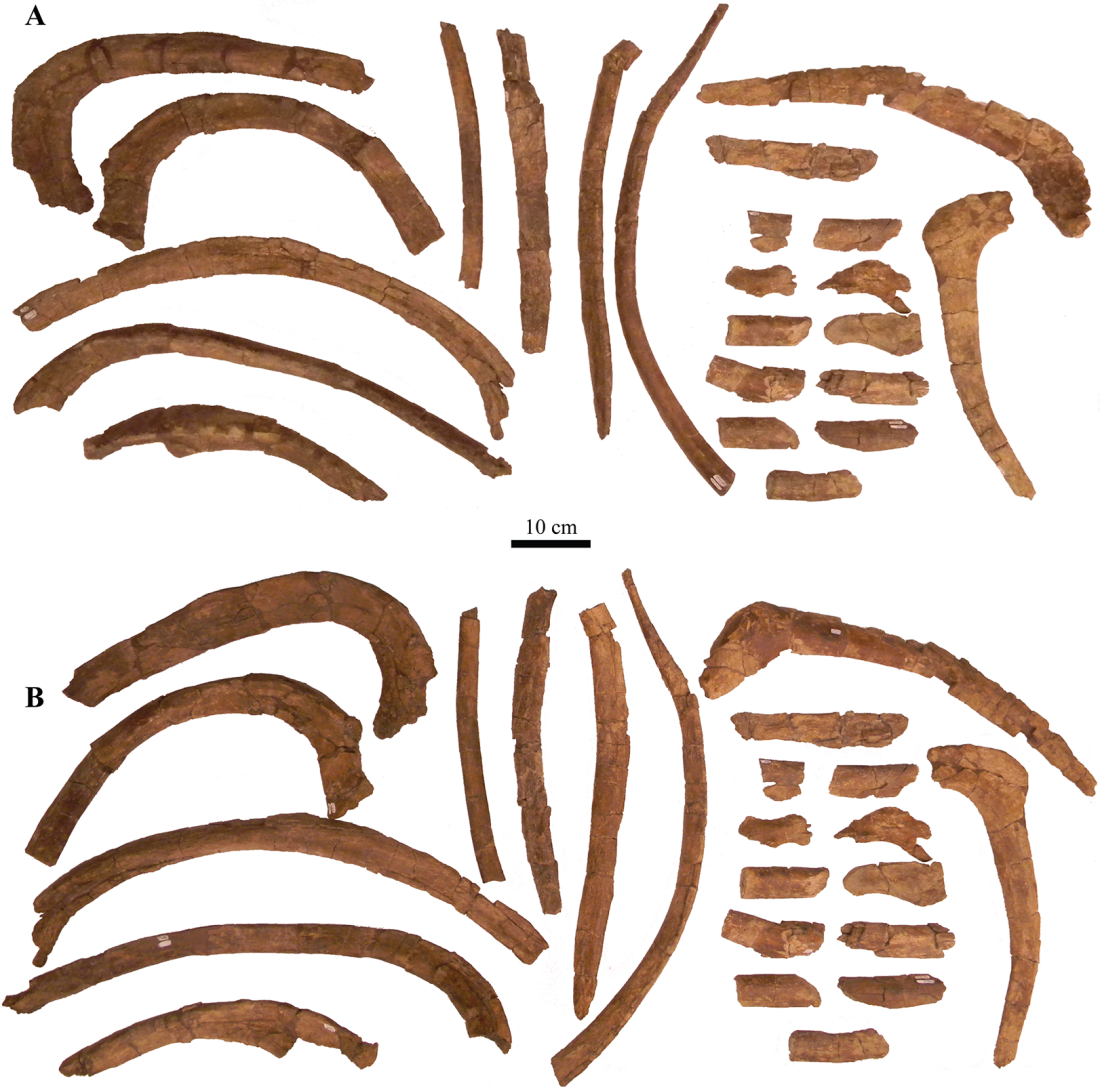
Ribs and rib fragments. Photographs of ribs and rib fragments of *Akainacephalus johnsoni* (UMNH VP 20202) in (A), anterior; and (B), posterior views.

In addition to the ribs fused to the posterior dorsal vertebrae (see above), a suite of 12 large dorsal rib fragments is preserved and represents eight proximal and four distal shafts, various mid shafts, and a number of small fragments (Fig. 16). The proximal rib fragments are typical of ankylosaur morphology, possessing horizontally expanded dorsal surfaces which form a T- or L-shaped cross-sectional morphology. Three proximal rib fragments possess an L-shaped morphology and arch sharply ventrally. Most of the capitula and tubercula are eroded away. The proximal shaft is dorsoventrally tall but tapers distally along the rib length until it becomes subrounded, and this morphology continues along the remainder of the rib, based on the four preserved distal fragments. A total of five T-shaped proximal rib fragments are preserved and possess wide horizontal expansion on the dorsal surface of both sides of the ribs. The T-shaped ribs arch less sharply ventrally compared to the L-shaped ribs, which might suggest that the T-shaped ribs are situated further posterior along the vertebral column, where the wider part of the rib cage is positioned. Many smaller rib fragments comprise 5–10-cm-long sections of the distal shafts.

## Appendicular Skeleton

### Scapula and coracoid

**Figure 17:**
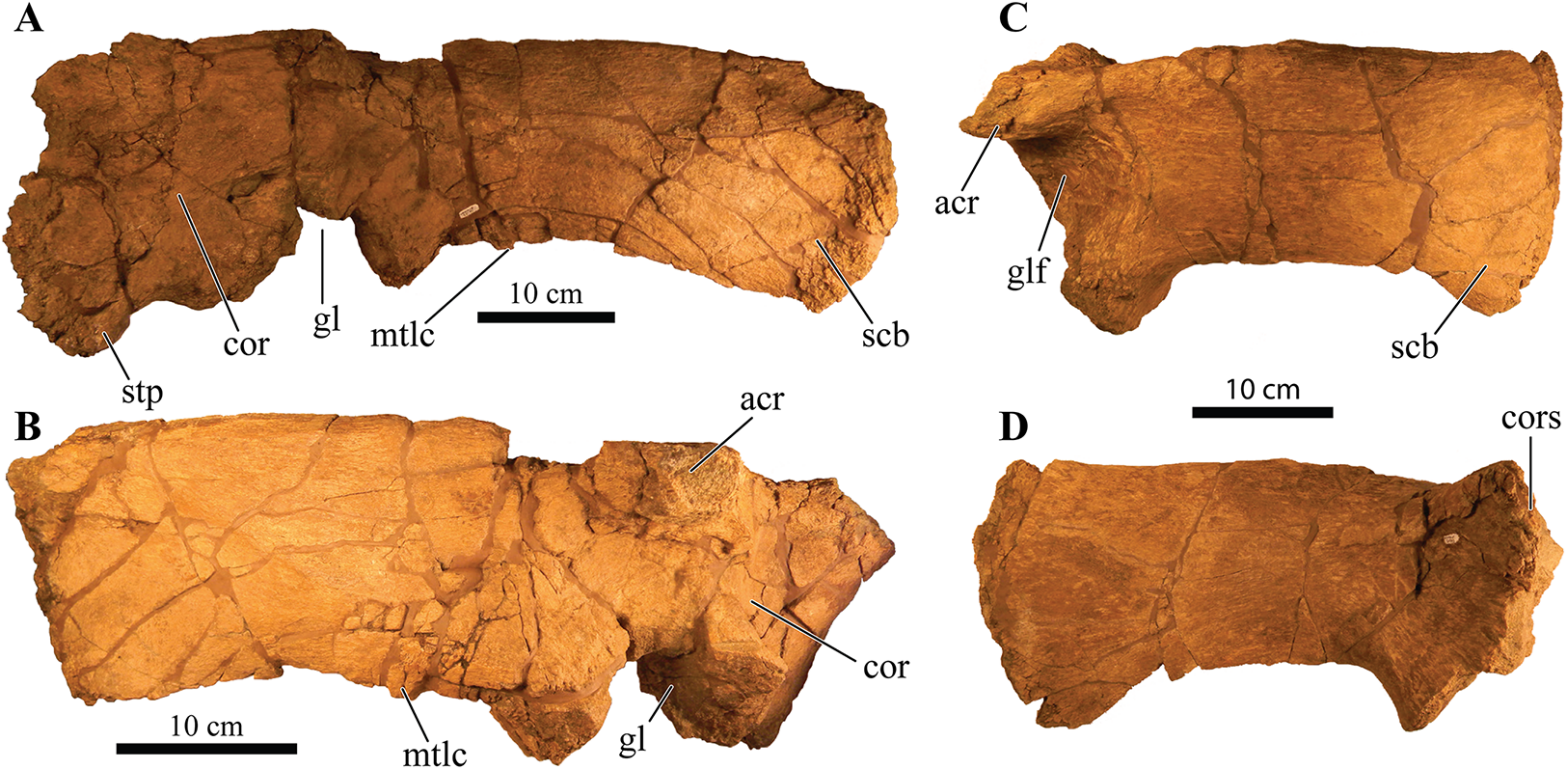
Left and right scapulae and right coracoid. Photographs of the left and right scapulae and right coracoid of *Akainacephalus johnsoni* (UMNH VP 20202). Right scapula and coracoid in (A), medial; and (B), lateral views. Left scapula in (C), lateral; and (D), medial views. Study sites: acr, acromium; cor, coracoid; gl, glenoid; glf, glenoid fossa; mtlc, enthesis of *M. triceps longus caudalis*; scb, scapular blade; stp, sternal process.

Both scapulae and a left coracoid are preserved (Fig. 17). The scapulae are laterally convex and medially concave in dorsal and ventral view, curving gently along the long axis of the scapular blade. The dorsal scapular process contributes partially to the articular contact with the coracoid and is positioned directly adjacent to the acromion process. The dorsal process expands medially but is still less than 75% of the minimum dorsoventral height of the scapular blade. The acromion process is positioned on the dorsal surface of the scapula, directly above the glenoid fossa, and forms a dorsoventrally thick and laterally protruding flange. This particular morphology is also observed in *Euoplocephalus tutus* (Arbour & Currie, 2013a) and *Ankylosaurus magniventris* (Carpenter, 2004). In *Ahshislepelta minor* (Burns & Sullivan, 2011a), the acromion process is positioned further caudally and is above the posterior half of the glenoid. In cross section, the acromion process projects perpendicular to the lateral surface of the scapular blade. Anteriorly, the acromion process contributes partially to the articular contact with the coracoid. Together with the scapular portion of the glenoid fossa, they form a deep sulcus on the lateral side of the scapula (Fig. 17C). The glenoid fossa is a ventrally oriented, bipartite structure composed of the larger anteroventral margin of the scapula and the adjacent, smaller, posteroventral margin of the coracoid. The dorsal margin of the glenoid fossa expands laterally, forming a shallow, lateroventrally oriented dorsal shelf that surrounds the humeral head (Figs. 17A and 17B). In other ankylosaurid dinosaurs (e.g., *Ahshislepelta minor* (Burns & Sullivan, 2011a), *Euoplocephalus tutus* (Arbour & Currie, 2013a), *Ankylosaurus magniventris* (Carpenter, 2004), and *Pinacosaurus grangeri* (Hill, Witmer & Norell, 2003)), the posterior half of the glenoid fossa that contributes to the scapula is laterally thicker, anteroposteriorly larger, and dorsoventrally taller compared to the anterior half of the glenoid fossa that articulates with the coracoid. In lateral view, the morphology of the scapular blade is nearly straight, with a minor convex dorsal surface, similar to *Euoplocephalus tutus* (Arbour & Currie, 2013a). In contrast, in *Ankylosaurus magniventris* (Carpenter, 2004) this dorsal surface is strongly convex dorsally, and in *Saichania chulsanensis* (Maryańska, 1977) it is slightly concave. A distinct surface for the insertion of the *M. triceps longus caudalis* is situated on the ventral surface of the scapular blade, directly posterior to the area where the blade is at its minimum dorsoventral height. In lateral view, the scapular blade is paddle-shaped with a convex distal margin, similar to *Euoplocephalus tutus* (Arbour & Currie, 2013a). This morphology is the result of dorsoventral expansion of the distal margin, primarily along the ventral margin, and the total expansion attributes more than 150% of the minimum dorsoventral height of the scapular blade.

The somewhat square coracoid is anteroposteriorly longer than it is dorsoventrally tall, and is fused to the right scapula. The coracoid is oriented anteromedially compared to the scapular blade. The posteromedial surface that articulates with the scapula is medially expanded to form a uniform structure with the dorsal scapular process (Figs. 17A and 17B). A large oval coracoid foramen is situated directly dorsal to the glenoid fossa. The dorsal and anterior surfaces are largely broken but appear to be concave. In cross section, the dorsal border of the coracoid is convex, whereas the ventral border is concave. A short anteroventral process is present and is anteriorly separated from the glenoid by a distinct ventrally concave notch.

### Humerus

**Figure 18:**
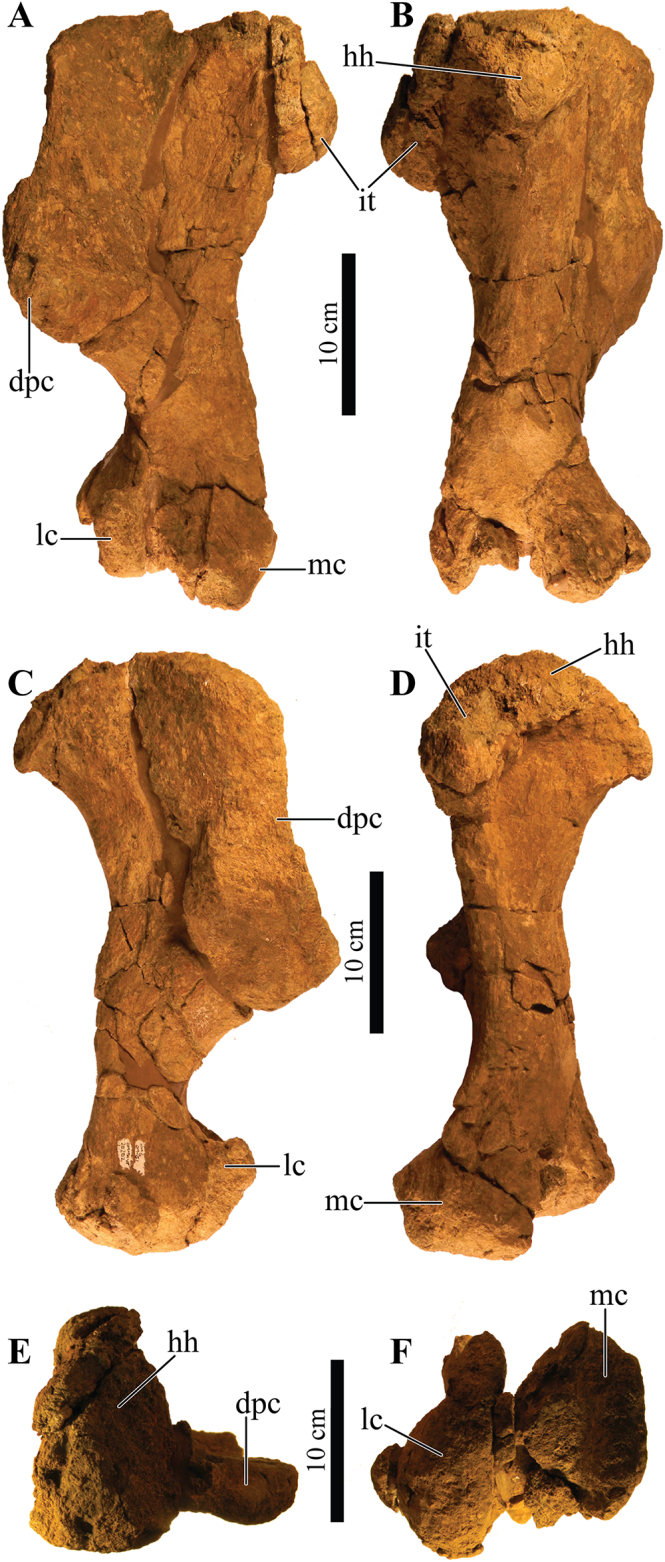
Right humerus. Photographs of the right humerus of *Akainacephalus johnsoni* (UMNH VP 20202) in (A), anterior; (B), posterior; (C), lateral; (D), medial; (E), dorsal; and (F), ventral views. Study sites: dpc, deltopectoral crest; hh, humeral head; it, internal tuberosity; mc, medial condyle; lc, lateral condyle.

A complete right humerus and a partial left humeral head are preserved. The right humerus is robust like other ankylosaurids; an enlarged and tall deltopectoral crest spans >50% of the total humeral length and is followed by a short and thick humeral shaft that comprises the ventral half of the humerus. Most of the external surface experienced predepositional weathering, removing most of the original surface texture and subsequently eroding away the medial and dorsal margins of the humeral head. The remaining portion of the humeral head is separated from the greater tubercle by a small notch which is commonly seen in most other ankylosaurids, except *Ankylosaurus magniventris* (Carpenter, 2004) and *Crichtonpelta benxiensis* (=*Crichtonsaurus*, Junchang et al., 2007), which lack a notch and form a continuous surface between these elements. Posterior and directly ventral to the humeral head is a large, well-expressed, posteroventrally projecting process that terminates in a ventrally projecting hook (Figs. 18C and 18D). In some ankylosaurids this process is absent (i.e., AMNH FARB 5337) but expressed to some extent in others (e.g., AMNH FARB 5404), however, it is prominent *Akainacephalus johnsoni*. Directly ventral to the process, the humeral shaft expands posteriorly, resulting in distinctly separated humeral heads of the triceps (Figs. 18A and 18C). Anteriorly, the area between the humeral head and the greater tubercle (supracoracoideus) form a large but shallow sulcus. The lateral external margin of the greater tubercle curves downward and forms a continuous surface that transitionss into the mediotransversely expanded and strongly anterolaterally oriented deltopectoral crest. A large and round protuberance is situated on the lateral side of the deltopectoral crest and forms the articulation surface for the *M. latissimus dorsi*. The internal tuberosity is damaged but large and well expressed (Figs. 18A, 18B and 18D). Distally, the humeral shaft is followed by the medial and lateral epicondyles. Both epicondyles suffered postdepositional breakage and deformation, resulting in the removal of the articular surface on the medial epicondyle (Fig. 18D), whereas the semicircular articular surface on the lateral epicondyle is preserved and bears a prominent protuberance on the anterior margin (Fig. 18C). The medial epicondyle extends slightly more distal compared to the lateral epicondyle (Figs. 18A and 18B). In anterior and posterior view, the epicondyles are separated by a shallow concave sulcus (Figs. 18A and 18B); however, the ventral-most portion is deeply excavated due to breakage and erosion (Fig. 18F). Measurements of the humerus are summarized in Supplemental Material S4.

### Ulna

**Figure 19:**
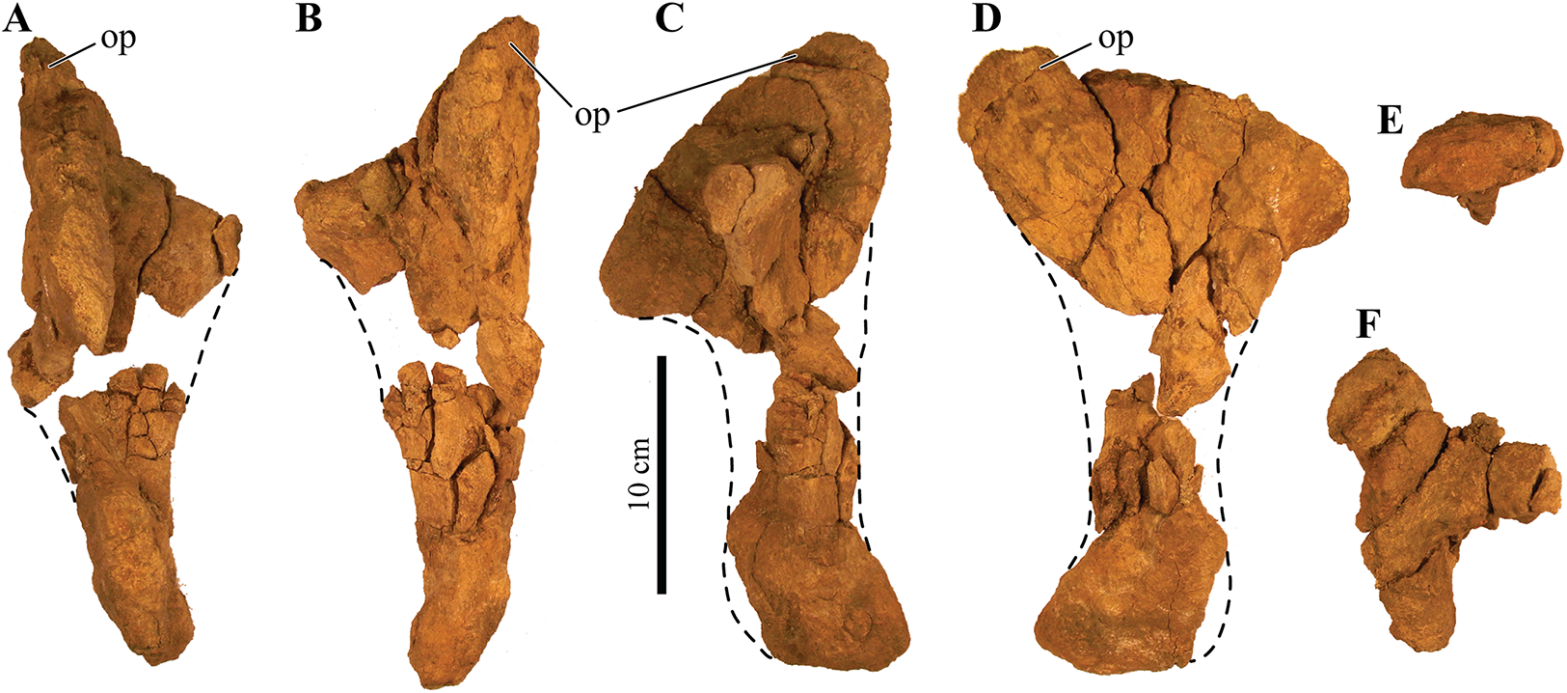
Left ulna. Photographs of a partial left ulna of *Akainacephalus johnsoni* (UMNH VP 20202) in (A), anterior; (B), posterior; (C), lateral; (D), medial; (E), distal; and (F), proximal views. Study sites: op, olecranon process.

Only the left ulna is present, but it is broken along the mid-shaft and largely incomplete, preserving only the olecranon process and a partial shaft (Fig. 19). Most of the olecranon process is broken away, leaving only the base preserved. The coronoid process is transversely distorted and is poorly preserved, missing most of the ventral margin. The radial notch has a typical ankylosaur morphology and is mostly intact, forming a large, laterally projecting flange with a ventrally tapering shaft, from which small fragments are broken away. Little is left of the distal end of the ulna, but the posteriorly oriented flange of the ulna head is still visible.

### Ilium

**Figure 20:**
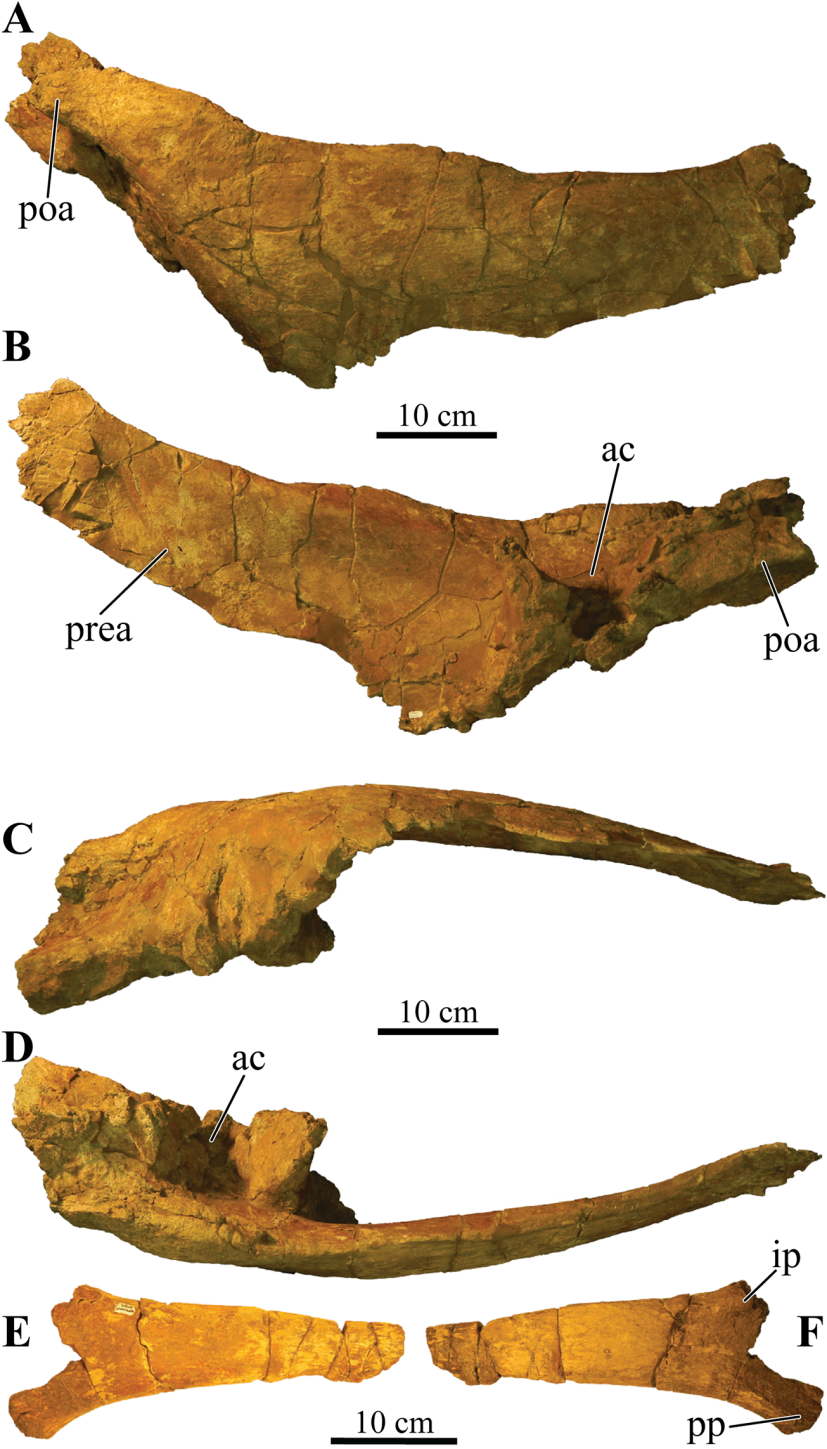
Left ilium and right ischium. Photographs of the left ilium and right ischium of *Akainacephalus johnsoni* (UMNH VP 20202). Left ilium in (A), dorsal; (B), ventral; (C), lateral; and (D), medial views. Right ischium in (E), medial; and (F), lateral views. Study sites: ac, acetabulum; ip, iliac peduncle; poa, postacetabular process; pp, pubic peduncle; prea, preacetabular process.

A nearly complete left ilium is preserved and shows some breakage along the acetabulum, ventral margin of the ilium, and the distal margins on the pre- and postacetabular processes (Figs. 20A–20D). In dorsal view, the ilium is broadly concave along the lateral margin and nearly straight medially, but dorsally convex in medial and lateral views (Figs. 20A, 20C and 20D). The dorsal portion of the acetabular region curves laterally and ventrally, forming a broad supra-acetabular shelf that extends laterally and obscures the acetabulum in lateral view (Fig. 20C). Articular surfaces for the attachement of sacral ribs are not preserved on the ilium and are difficult to identify on the along the medial margin of the acetabulum, pre- and postacetabular processes. The large preacetabular process forms >50% of the entire iliac length and projects anterolaterally at an angle of ∼23°. Most of the medial and anterior margins are broken and would have provided the contact surfaces for the dorsosacral ribs. Posteriorly, the postacetabular process forms a short buttress, but it is longer than the acetabulum and curves strongly posteroventrally at ∼45°. A large portion of the posterior margin is postdepositionally broken. A deep, concave sulcus is located on the medial side of the ilium and forms the ventrally oriented, closed acetabulum. The acetabulum itself is only partially preserved, and it shows breakage particularly around the articular surfaces that contact the proximal margins of the pubis and ischium.

### Ischium

A partial, right ischium is preserved (Figs. 20E and 20F) and has a typical ankylosaurid morphology; it is Y-shaped in lateral and medial view, and comprises a laterally compressed shaft, containing virtually no anterior curvature, consistent with ankylosaurid ischia (Vickaryous, Maryańska & Weishampel, 2004). The lateral surface of the ischium is relatively straight in anterior and posterior view but the medial surface is slightly concave. Most of the proximal part of the ischium that forms the ischial and pubic peduncles are broken away, making it difficult to distinguish between a left or right ischium. A small remnant of the lateral sulcus is preserved on the dorsolateral part of the ischium and contributes to the closed acetabulum. The distal-most end of the ischium is also broken so an exact length of the element cannot be determined.

### Femur

**Figure 21:**
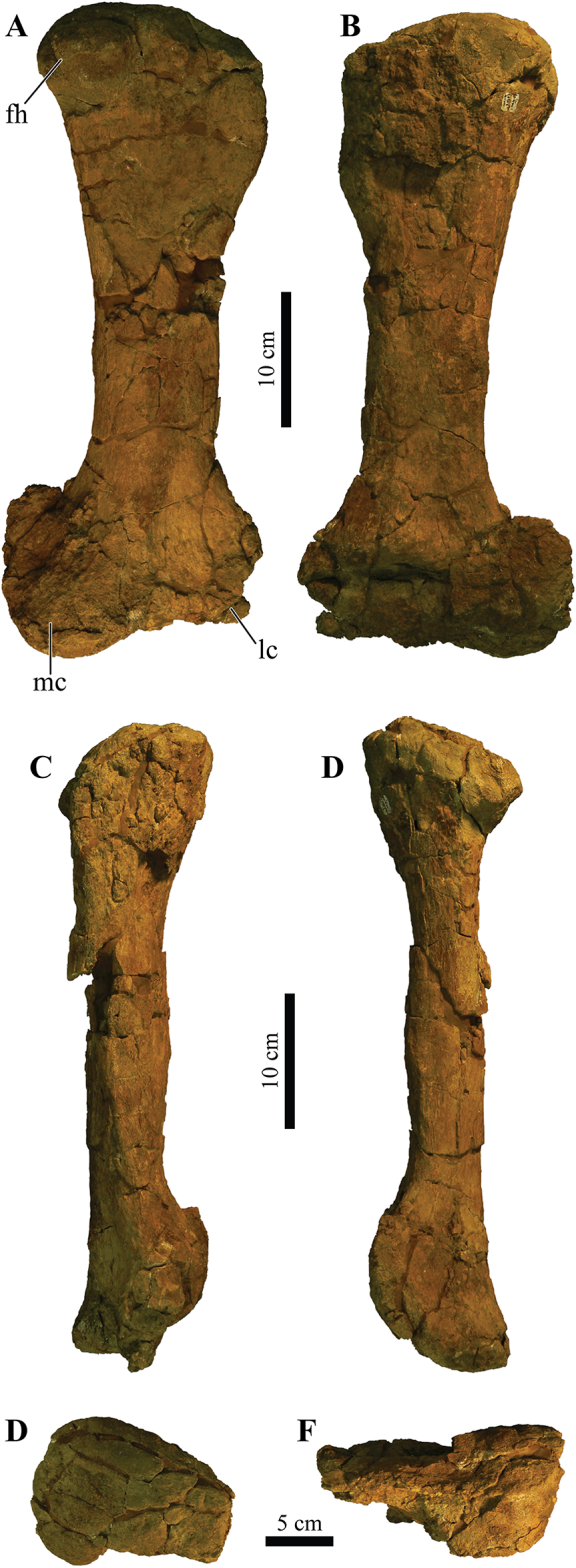
Left femur. Photographs of the left femur of *Akainacephalus johnsoni* (UMNH VP 20202) in (A), anterior; (B), posterior; (C), lateral; (D), medial; (E), dorsal; and (F), ventral views. Study sites: fh, femoral head; lc, lateral condyle; mc, medial condyle.

A partially broken and anteroposteriorly compressed left femur is preserved (Fig. 21). Both the medial and lateral condyles are heavily distorted and compressed against the femoral shaft. Most of the condylar surfaces are eroded away, leaving a broken distal surface, which is particularly evident on the lateral condyle. The femoral head is an ellipsoid, convex articular surface that nearly expands the entire width of the proximal femur. In anterior and posterior view, the femoral head slopes downward laterally, forming an asymmetrical but continuous surface with the lateroproximally positioned greater trochanter, which is appressed with the femoral shaft. The dorsal surface between the femoral head and the greater trochanter is slightly elevated, forming a small protuberance. On the femur of *Euoplocephalus tutus* (AMNH FARB 5404), this protuberance is clearly visible (Arbour & Currie, 2013a: fig. 12I). This condition appears common among most other ankylosaurids; however, a shallow notch (“neck” in Carpenter, 2004) between the femoral head and greater trochanter has been observed in *Dyoplosaurus acutosquameus* (ROM 784) and *Ankylosaurus magniventris* (AMNH FARB 5214). An appressed greater trochanter is present in both stegosaurs and most ankylosaurs (Sereno, 1999), but appears separated in some polacanthids. The long axis of the femoral head in *Akainacephalus johnsoni* forms an angle of ∼70° with the long axis of the femoral shaft, indicating a near horizontally oriented femoral head. This condition is common in all Late Cretaceous Laramidian ankylosaurids, whereas all contemporaneous Asian ankylosaurids where the element is preserved possess femora with angles less than 70°, forming a more horizontal orientation of the femoral head. The femoral trochanter is a longitudinal ridge (Fig. 21C) that is positioned medially along the distal half of the femoral shaft.

### Tibia

**Figure 22:**
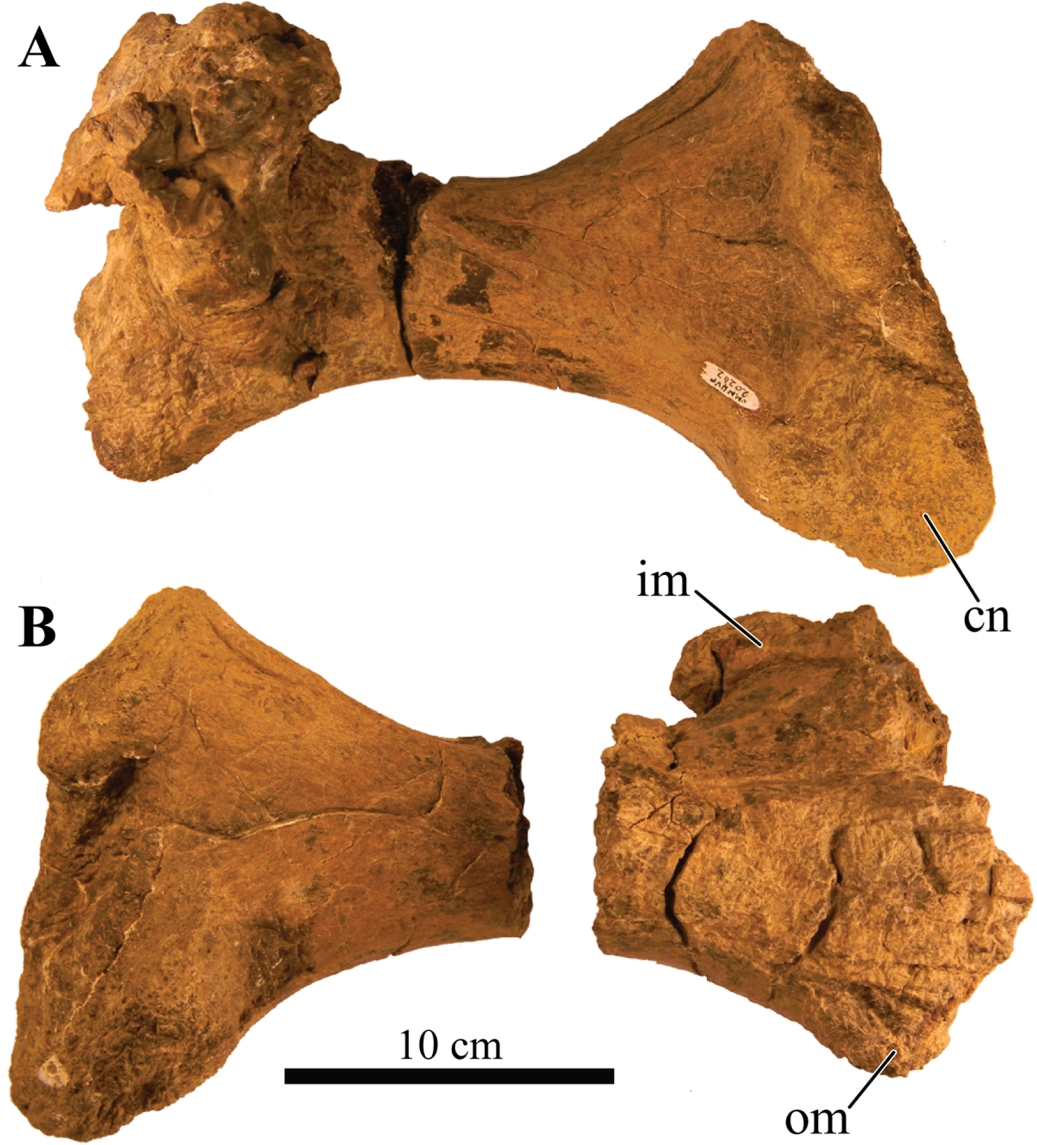
Right tibia. Photographs of the right tibia of *Akainacephalus johnsoni* (UMNH VP 20202) in (A), anterior; and (B), posterior views. Study sites: cn, cnemial crest; im, inner malleolus; om, outer malleolus.

A partial right tibia is preserved (Fig. 22). A portion of the short and robust midshaft is missing. Overall, it represents a fairly robust element, with a short diaphysis and broad articular ends typical for thyreophoran dinosaurs; however, it is heavily deformed proximally, making it difficult to interpret the original morphology. The element experienced extensive plastic deformation, flattening the distal condyles anteroposteriorly, but the general morphology is similar to tibiae observed in other ankylosaurids. In addition, the dorsal part of the tibial crest experienced diagenetic alteration through development of small iron concretions along the dorsal and anterior portions of the crest. The posteromedially situated cnemial crest is severely distorted and mostly broken off. Along the lateral side on the distal end, the lateral malleolus is anterolaterally deformed and therefore extends further laterally than in life. The medial malleolus is broken away. Furthermore, additional erosion has removed the original distal surface texture.

### Fibula

**Figure 23:**
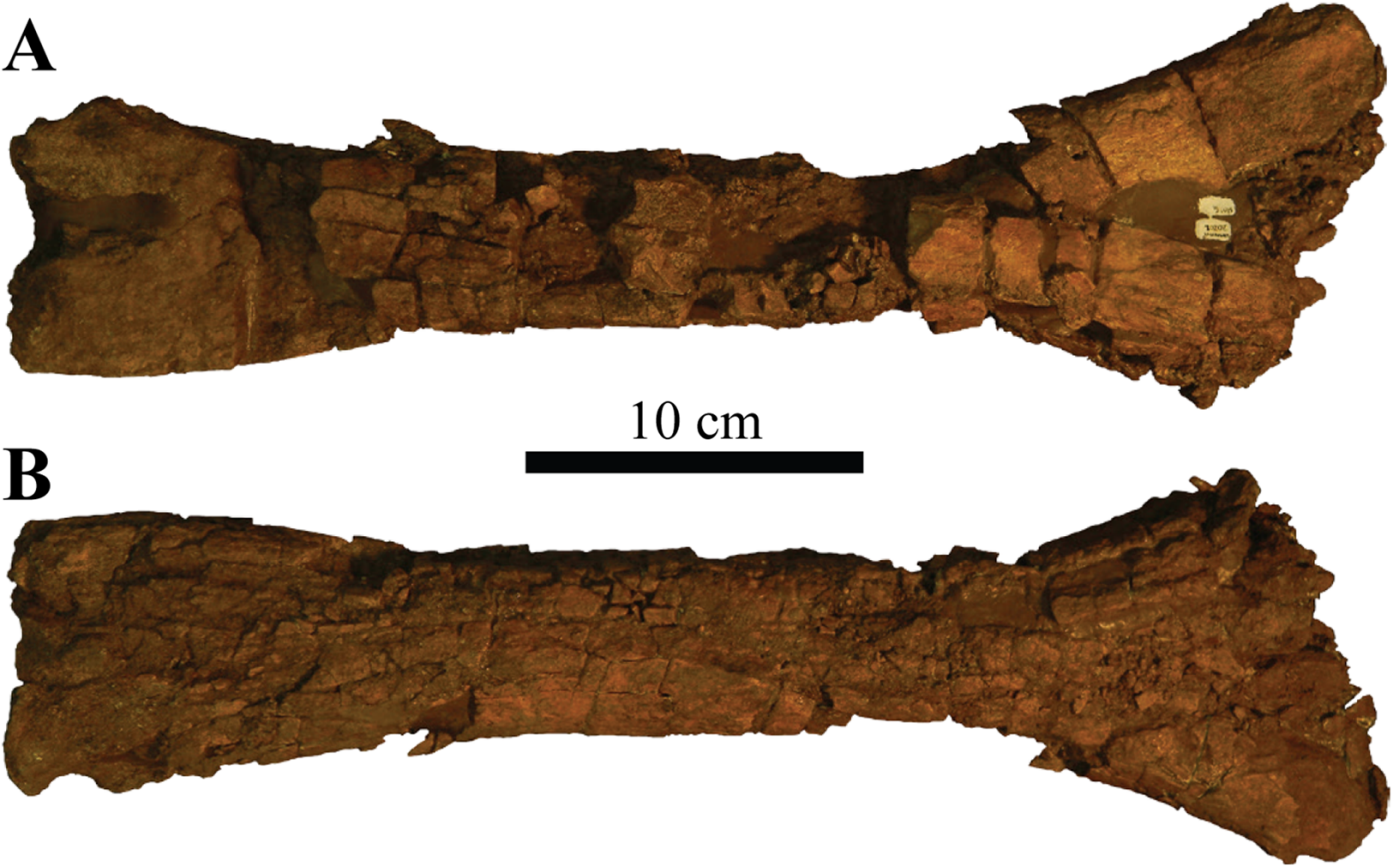
Left fibula. Photographs of the left fibula of *Akainacephalus johnsoni* (UMNH VP 20202) in (A), posterior; and (B), anterior views.

A single fibula was collected but its state of preservation is very poor and it experienced severe breakage and surface weathering, causing most important anatomical features to be missing (Fig. 23). Thus, the anatomical description is largely based on comparison with other ankylosaurid specimens of *Ankylosaurus magniventris* (AMNH FARB 5214) and *Euoplocephalus tutus* (AMNH FARB 5266).

The fibula is long and narrow. Proximally, the shaft expands to form an asymmetrical anteroposteriorly wide surface. A small depression appears to be present on this surface and could potentially represent the medial face based on the morphology of *Ankylosaurus magniventris* (AMNH FARB 5214) (Brown, 1908; Carpenter, 2004). Minor anteroposterior expansion due to mediolateral compression occurs along the distal portion of the fibula, which contains the malleolus of the fibula, but the articular surfaces are highly damaged. The fibula is relatively long (397 mm) but ∼14% shorter than the femur (464 mm), a condition which is not uncommon in ankylosaurid dinosaurs. However, the femur/fibula length ratio is 1.17, which is unusually low compared to other ankylosaurids. For example, *Euoplocephalus tutus* (e.g., AMNH FARB 5266 (Arbour & Currie, 2013a: fig. 12; Coombs, 1986: fig. 2)) has a ratio of 1.55 and *Ankylosaurus magniventris* (AMNH FARB 5214 (Carpenter, 2004: fig. 18)) a ratio of 1.53.

### Pedal phalanx

**Figure 24:**
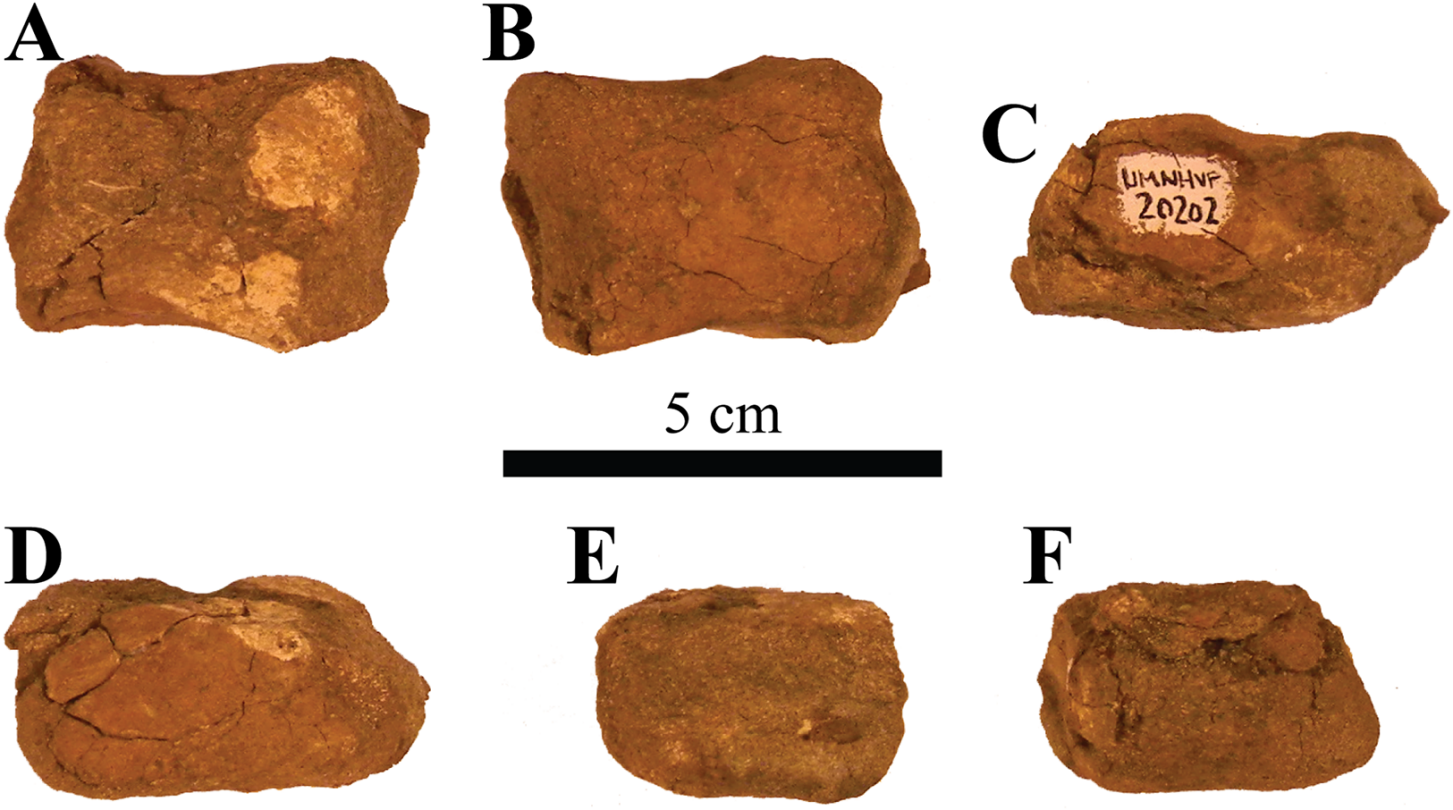
Pedal phalanx. Photographs of a single pedal phalanx of *Akainacephalus johnsoni* (UMNH VP 20202) in (A), anterior; (B), posterior; (C), dorsal; (D), ventral; (E), lateral; and (F), medial views.

A single, small metapodial element is preserved (Fig. 24) and shows heavy weathering around its lateral and medial margins, making exact identification difficult. The element is transversely wider than long and is slightly concave along its anterior and posterior articular surfaces. Based on this morphology, it is probably a proximal pedal phalanx (Currie et al., 2010).

### Ungual

A small fragment was collected with UMNH VP 20202 that could potentially represent the left lateral and anterior-most part of an ungual. The fragment is broken posteriorly and medially and displays dense internal bone structures and vascularity. Towards the midline, the bone appears excavated, suggesting a hollow cavity in part of the ungual. Anteriorly, the element contains a distinct, blunt keel that runs transversely along the anterior surface. The ventral surface is flat and becomes convex anteriorly, has a slightly weathered but smooth surface texture compared to the dorsal surface, which is entirely convex and displays several vascular canals.

## Postcranial Armor

### Cervical half rings

**Figure 25:**
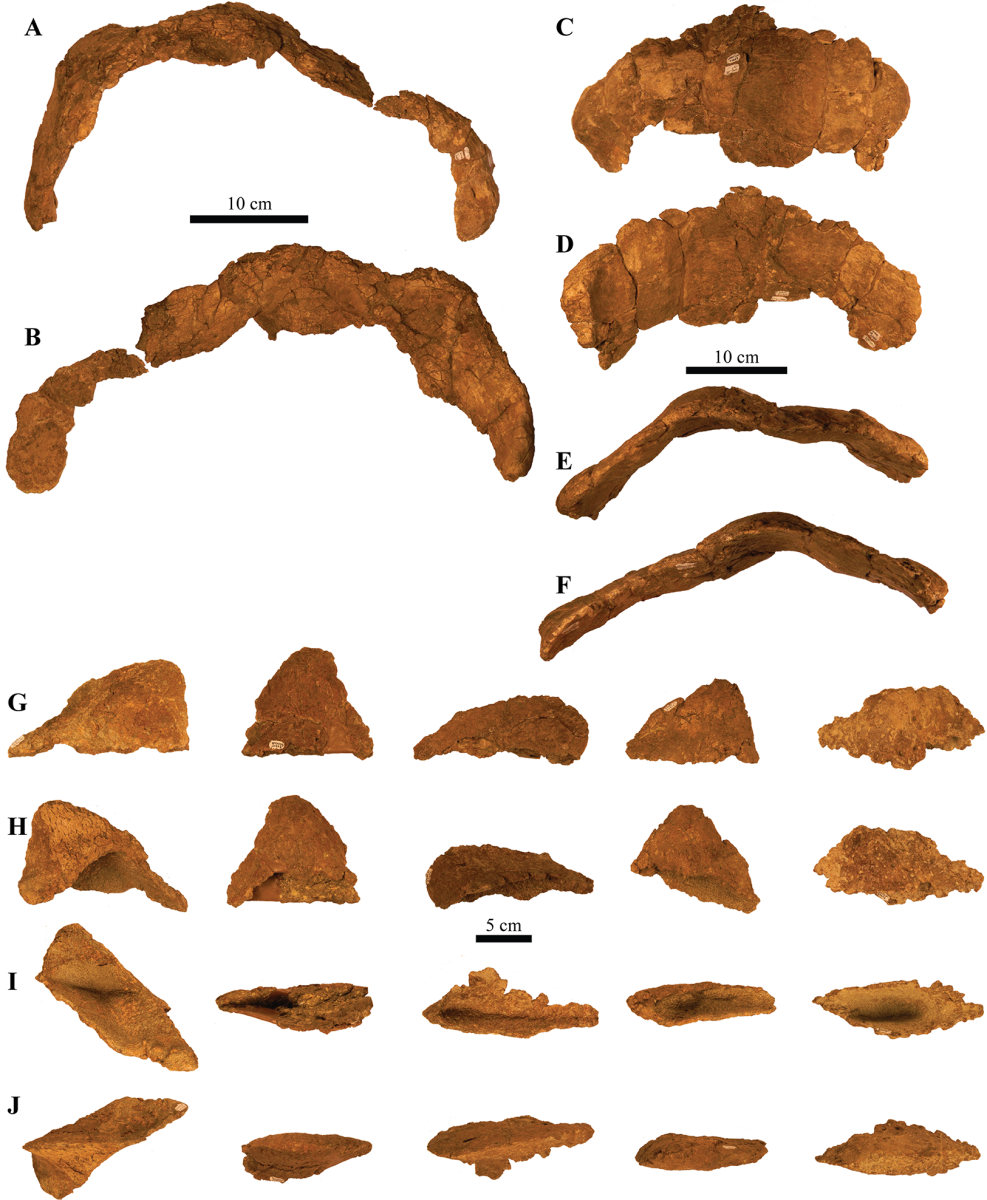
Cervical half rings and postcranial osteoderms. Photographs of postcranial osteoderms of *Akainacephalus johnsoni* (UMNH VP 20202). Nearly complete cervical half ring in (A), anterior; and (B), posterior views. Right lateral portion of the partial half ring in (C), dorsal; (D), ventral; (E), posterior; and (F), anterior views. Various sharply keeled, type 1 (pup-tent), osteoderms associated with UMNH VP 20202. The osteoderms are displayed in (G), dorsal; (H), ventral; (I), medial; and (J), lateral views. No consensus currently exists regarding the exact position of type 1 osteoderms, but they appear to be positioned either laterally on the body along the sacral and proximal portion of the tail or dorsolaterally along the thoracic and pectoral regions as seen in *Euoplocephalus* and *Scolosaurus* (Maryańska, 1977; Ford, 2000).

A partial anterior cervical half ring and a nearly complete posteriorcervical half ring is preserved (Figs. 25A–25F). Both cervical half rings are broken along the anterior and posterior margins, so the exact shape of these surfaces is unclear. The incomplete element (Figs. 25A–25D) is possibly the anterior-most (first) half ring, and the near-complete element (Figs. 25E and 25F) is likely the posterior (second) half ring. The presence of dorsoventrally raised and thickened surfaces along the dorsal and lateral regions of each half ring suggests they are comprised of six individual secondary osteoderms, similar to *Euoplocephalus tutus* (CMN 210, UALVP 31), *Anodontosaurus lambei* (CMN 8530), and *Scolosaurus cutleri* (Penkalski & Blows, 2013). The secondary osteoderms are heavily fused with the dorsal surfaces of the half rings and possibly experienced resorption to some extent, however, the dorsal surface of each half ring is severely weathered, making it challenging to assess the morphology of individual osteoderms. Several ankylosaurid dinosaurs such as *Euoplocephalus tutus* (e.g., AMNH FARB 5337, UALVP 31, UALVP 45931, USNM 7943, TMP 2007.12.52) display distinct and clearly visible saw-tooth sutures across the junctures of the half ring segments, fusing together the individual bony segments that make up the cervical band (see Figs. 13H–13K and 13T–13V in Arbour & Currie, 2013a). These sutures were not observed in *Akainacephalus johnsoni*.

The overall morphology of the half rings is ventrally concave and dorsally convex; however, when observed more closely in anterior and posterior view, the loci that accommodate the secondary osteoderms are thickened and elevated compared to the empty interstitial areas, resulting in a profile that is rather sinusoidal (Figs. 25A, 25E and 25F). These dorsally elevated loci are bulbous and oval, with an anteroposteriorly oriented long axis, suggesting that the overlying secondary osteoderms would have had excavated bases and possibly a similar oval circumference. A total of two large foramina are present and situated dorsal to the ventral-most articular surfaces for the secondary osteoderms.

### Other noncervical osteoderms

A total of 14 noncervical partial osteoderms are preserved and provide some insight regarding the morphology and possible locations of armor across the body of UMNH VP 20202. The majority of the osteoderms are anteroposteriorly elongate and mediolaterally compressed. All the osteoderms are tall, with strongly keeled dorsal margins (Figs. 25G–25J), and contain deeply excavated bases, the so-called “pup-tent” morphology (Ford, 2000), and they contain slightly vascular and heavily pitted surface textures. Their sizes range between a few centimeters and >10 cm in length. Little is known about the exact locations and positions of postcranial osteoderms in ankylosaurid dinosaurs because they commonly dissociate and disarticulate from the body after death. Only a few known specimens, including *Scolosaurus cutleri* Nopcsa, 1928 (NHMUK R5161, holotype), *Dyoplosaurus acutosquameus*, Parks, 1924 (ROM 784, holotype), *Zuul crurivastator*, Arbour and Evans, 2017, *Pinacosaurus grangeri* Gilmore, 1933 (IVPP V16853, PIN 614 (“*Syrmosaurus*”: Maleev, 1954)), cf. *Pinacosaurus*, Arbour and Currie, 2013b (MPC 100/1305 (“*Saichania chulsanensis*”: Carpenter et al., 2011)), and ZPAL. MgDI/113 (previously referred to as *Tarchia* cf. *gigantea* Maleev, 1956), preserve osteoderms articulated with the rest of the skeleton, providing at least partially detailed anatomical information regarding the position and orientation of postcranial ornamentation among ankylosaurid dinosaurs. Combined with other studies on ankylosaurian osteoderms by other workers (Maryańska, 1969; Carpenter, 1982; Burns, 2008; Arbour, Burns & Sissons, 2009; Penkalski, 2014, Arbour et al., 2013; Burns & Currie, 2014; Brown et al., 2017; Brown, 2017) these data provide additional insights on the positions of postcranial osteoderms preserved within *Akainacephalus johnsoni*. Thus, based on the morphology and base width of the preserved osteoderms in *Akainacephalus johnsoni*, it appears that their locations are predominantly positioned along the dorsal (wider base) and lateral (narrower base) portions of the body, and possibly the forelimbs (Lambe, 1902; Parks, 1924; Nopcsa, 1928; Sternberg, 1929; Carpenter, 1982; Ford, 2000; Carpenter, 2004). Preserved osteoderms are subdivided into three distinct morphologies based on morphotypes described in detail by Maryańska (1969) and include: type 1, “pup-tent” shaped (Ford, 2000) with S-twisted keels and a backswept apex; type 2, dorsoventrally compressed triangular; and type 5, flat-based with an off-centered, longitudinal keel.

Five type 1 osteoderms, referred to as “pup-tent” shaped (Figs. 25G–25J) by Ford (2000), are taller than wide with narrow, deeply excavated bases, and thin-walled. Most are incomplete, with portions of the base broken away. The osteoderms are asymmetrical in lateral view, with tall, sharply pronounced keels that are anteriorly convex and nearly twice as long as the short concave anterior margin. This morphology results in dorsoposteriorly backswept and rounded apexes (Figs. 25G and 25H). In dorsal view, the keels are sinusoidal (= S-twist in Maryańska, 1969) compared to the straight keels observed in the type 2 and type 5 osteoderms described below. Surface texture ranges from minimally rugose with some dorsoventral furrowing in some osteoderms to rugose and densely distributed, shallow pitting in others (Figs. 25G and 25H). Type 1 osteoderms appear to be positioned on the thoracic region of the body as seen in *Euoplocephalus tutus* (Ford, 2000) or the pectoral region in *Scolosaurus cutleri* (Penkalski & Blows, 2013).

Eight partial type 2 osteoderms of various sizes (55–112 mm long and 65–91 mm tall) are dorsoventrally compressed with narrow but deeply excavated concave bases and display a triangular dorsoventral morphology that terminates laterodistally in a distinct apex. Their surface texture is slightly rugose with minor transverse running, shallow furrows, and minimal pitting. These morphologies are typical for osteoderms positioned along the lateral margins of the thorax and tail, as is apparent in specimens of *Scolosaurus cutleri* (Penkalski & Blows, 2013), *Dyoplosaurus acutosquameus* (ROM 784), *Tarchia teresae* (Penkalski & Tumanova, 2017), and *Saichania chulsanensis* (Maryańska, 1977), but the type 2 osteoderms in *Akainacephalus johnsoni* lack the two distinct dorsal grooves that were observed by Arbour et al. (2013) in *Tarchia*. The entire external margins are keeled along the posterior and anterior sides, including the apex. One side is convex and the other side is concave, but it is difficult to determine the posterior and anterior face because the orientation of these osteoderms varies among different ankylosaurid taxa. For example, the lateral osteoderms on the proximal and distal tail of ZPAL MgD I/113 (*Tarchia* cf. *gigantea* (Arbour et al., 2013: fig. 4)) and proximal tail of *D. acutosquameus* (Parks, 1924: plate 1; Arbour, Burns & Sissons, 2009: fig. 1) show convex anterior and concave posterior faces with a posterolaterally projected apex. This condition contrasts with the orientation of lateral caudal osteoderms observed in *Saichania chulsanensis* (Arbour et al., 2013: fig. 4), in which the anterior face is concave and the posterior convex, followed by an anterolaterally projecting apex.

A poorly preserved, single, type 5 dorsal osteoderm is heavily crushed and distorted. The remaining fragments suggest a round, flat base with a distinctly off-centered, straight keel that runs longitudinally along its dorsal surface, a condition and morphology that somewhat resemble the dorsally positioned osteoderms in *Scolosaurus* (Penkalski & Blows, 2013). The visible surface texture is smooth and accompanied by small, shallow pits.

## Phylogenetic Analysis

In order to reconstruct the phylogenetic relationships of *Akainacephalus johnsoni* among other ankylosaurid dinosaurs, we conducted a phylogenetic analysis using cladistic parsimony. Characters for *Akainacephalus johnsoni* were scored and coded using the character descriptions and character matrix constructed by Loewen & Kirkland (2013), providing the most comprehensive taxon and character sampling for Ankylosauridae to date, with 35 taxa and 293 characters (Supplemental Material S2). However, character scorings for the majority of the in-group ankylosaurid taxa were verified or modified through personal observations of original specimens or casts and specimens in published literature, and are reflected in the matrix used for the phylogenetic analysis and interpretation of its results.

A total of 11 nonankylosaurid taxa were also included to make sure character-states were optimized correctly at the base of Ankylosauridae. The Jurassic ornithischian *Lesothosaurus diagnosticus* was constrained as the outgroup taxon because it is just basal to the Thyreophora–Neornithischia split and known from nearly complete remains (Crompton, 1968; Thulborn, 1971; Galton, 1978; Sereno, 1991). The 10 nonankylosaurid thyreophoran taxa include the early thyreophorans *Scutellosaurus* (Colbert, 1981), *Emausaurus* (Haubold, 1990), and *Scelidosaurus* (Owen, 1859), the stegosaurs *Huayangosaurus* (Dong, Tang & Zhou, 1982) and *Tuojiangosaurus* (Dong et al., 1977), the basal ankylosaurian *Minmi* (Molnar, 1980), the nodosaurids *Europelta* (Kirkland et al., 2013) and *Cedarpelta* (Carpenter et al., 2001), and the polacanthids *Mymoorapelta* (Kirkland & Carpenter, 1994) and *Gargoyleosaurus* (Carpenter, Miles & Cloward, 1998). The analysis contains 24 putative ankylosaurid taxa and include recently described taxa such as *Ziapelta sanjuanensis* (Arbour et al., 2014) and *Zaraapelta nomadis* (Arbour, Currie & Badamgarav, 2014), *Akainacephalus johnsoni* (described herein), as well as the recently re-evaluated *Dyoplosaurus acutosquameus* (Arbour, Burns & Sissons, 2009), *Scolosaurus cutleri* (Penkalski & Blows, 2013), and *Oohkotokia horneri* (Penkalski, 2014). *Pinacosaurus grangeri*, *Pinacosaurus mephistocephalus*, *Tarchia kielanae*, and *Tarchia teresae*, were all coded as separate operational taxonomic units. We treat *M. ramachandrani* as a valid taxon and separate operational taxonomic unit because we agree with Penkalski & Tumanova (2017) that it is not a synonym of *Tarchia kielanae* (Arbour, Currie & Badamgarav, 2014; Arbour & Currie, 2016). *Tianzhenosaurus youngi* (Pang & Cheng, 1998) and *Shanxia tianzhenensis* (Barrett et al., 1998) are considered synonyms of *Saichania chulsanensis* in Arbour, Currie & Badamgarav (2014) and Arbour & Currie (2016), however, the characters used to unite these taxa are poorly defined, and given rather general descriptions. *Tianzhenosaurus*, *Shanxia*, and *Saichania* are considered valid taxa and have been treated as individual operational units. Similarly, we consider *O. horneri* a separate taxon, not a juvenile synonym with *Scolosaurus cutleri* (contra Arbour & Currie, 2013a, 2016). The main focus of the phylogenetic analysis is to provide a phylogenetic hypothesis for the position of *Akainacephalus johnsoni*, not to discuss the in-depth the phylogenetic relationships of Ankylosauridae, which is far beyond the scope of this descriptive paper and is better suited for a more in-depth phylogenetic contribution which is currently in preparation by Loewen & Kirkland (2013).

The matrix (Supplemental Material S3) was constructed in Microsoft Excel and formatted in Mesquite v.3.01 build 658 (Maddison & Maddison, 2011). The analysis was conducted using TNT v.1.1 (Goloboff, Farris & Nixon, 2008), and carried out under the “traditional search” option, with 10,000 replications of Wagner trees under the tree bisection reconnection (TBR) algorithm, saving 10 trees per replication, followed by an additional round of TBR on those saved trees. All characters were unweighted, and characters 1, 2, 3, 7, 10, 13, 16, 18, 23, 25, 30, 36, 38, 48, 49, 54, 64, 87, 98, 101, 103, 104, 105, 140, 141, 143, 145, 148, 149, 156, 162, 165, 174, 177, 194, 201, 205, 209, 217, 229, 231, 232, 236, 237, 238, 260, 268, and 279 were ordered. Bremer values were also recorded, together with a bootstrap analysis (utilizing sampling with replacement and 10,000 replications).

**Figure 26:**
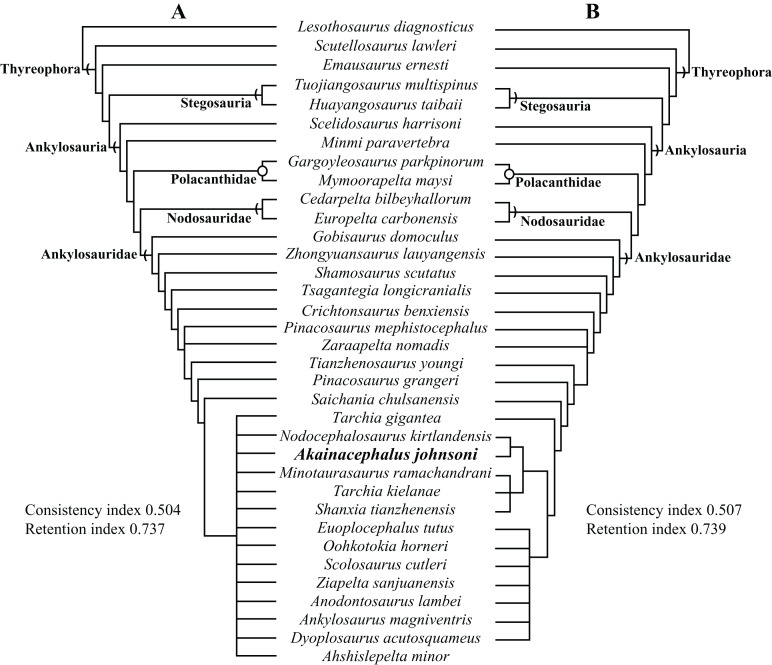
Results of phylogenetic analyses for *Akainacephalus johnsoni*. Phylogenetic position of *Akainacephalus johnsoni* in (A), a strict consensus of 21 equally most parsimonious trees, including the wildcard taxon *Ahshislepelta minor*, placing *A. johnsoni* within a large polytomy, consisting of crown group taxa that include Asian and all Laramidian ankylosaurids; and (B), the resulting strict consensus of six equally most phylogenetic trees, from which the wildcard taxon *Ahshislepelta minor* has been pruned. The crown group taxa are slightly better resolved in the pruned analysis, in which *Akainacephalus johnsoni* forms a clade with its sister taxon *Nodocephalosaurus kirtlandensis*, nested within the clade that also includes the Asian taxa *Minotaurasaurus ramachandrani*, *Tarchia kilanae*, and *Shanxia tianzhenensis*, suggesting a close taxonomic relationship with *Nodocephalosaurus kirtlandensis* and Asian taxa.

The analysis recovered 21 equally most parsimonious trees (length = 613), recording consistency index and retention index values of 0.504 and 0.737, respectively. Examining the strict consensus of these trees (Fig. 26A and Supplemental Material S1; Fig. S1), the base of the resulting phylogeny does not differ greatly from previous analyses (e.g., Carpenter, 2001; Hill, Witmer & Norell, 2003; Thompson et al., 2012; Arbour & Currie, 2016) of thyreophoran relationships. The ankylosaurids *P. mephistecephalus* and *Ziapelta sanjuanensis* form a small polytomy with the branch leading to *Tianzhenosaurus youngi* plus all other ankylosaurids (Fig. 26A). All other ankylosaurid taxa sister to *Saichania chulsanensis* form a large polytomy, including *Akainacephalus johnsoni* (Fig. 26A). In a majority of the most parsimonious trees, *A. johnsoni* is nested within a clade of Asian taxa. The high proportion of missing data for *Ahshislepelta minor* caused it to act as a wildcard taxon, collapsing the interrelationships of this large clade of ankylosaurid dinosaurs.

After removal of *Ahshislepelta minor*, the pruned dataset resulted in six most parsimonious trees (length = 610) (Supplemental Material S1; Figs. S2–S7) with a consistency index value of 0.507 and retention index value of 0.739. A strict consensus of these trees did not resolve the deeply nested polytomy of *P. mephistocephalus, Z. nomadis*, and the branch leading to *Tianzhenosaurus* plus all other ankylosaurids (Fig. 26B and Supplemental Materials S1; Fig. S8) but substantially more structure among the ankylosaurid taxa above *S. chulsanensis* that formed a large polytomy in the original analysis, placing *Tarchia teresae* as more closely related to a clade including all North American taxa than to *S. chulsanensis* (Fig. 26B). *Akainacephalus johnsoni* forms a clade with the southern Laramidian taxon *Nodocephalosaurus kirtlandenesis* (Fig. 26B), and is a sister clade to a polytomy of the Asian taxa *Tarchia kielanae*, *M. ramachandrani*, and *Shanxi tianzhenensis* (Fig. 26B). This southern Laramidian/Asian clade is sister to a clade including all other Laramidian taxa: *Ziapelta sanjuanensis*, *Ankylosaurus magniventris*, *Dyoplosaurus acutosquameus*, *Euoplocephalus tutus*, *Oohkotokia horneri*, *Anodontosaurus lambei*, and *Scolosaurus cutleri*, though their interrelationships are unresolved.

The clade of *A. johnsoni* + *N. kirtlandensis* is supported by five synapomorphies (Supplemental Material S1; Fig. S8): the width across the paroccipital processes is less than 195% of the height from the quadrates to the top of the paroccipital process (character 4[0]); lateral orientation of the external nares (character 10[0]); absence of a suborbital lip that forms a thin, sharp flange on the lateral edge along the ventral surface of the orbit (character 41[0]); posterolateral orientation of the supraorbital boss in anterior view (character 46[1]); and quadrate condyles visible in lateral view, and not obscured by the quadratojugal horn (character 57[0]). The clade (Supplemental Material S1; Fig. S8) that includes *T. kielanae*, *M. ramachandrani*, *S. tianzhenensis*, and *A. johnsoni* + *N. kirtlandensis* is supported by three synapomorphies: no dorsoventral expansion of the distal paroccipital process, compared to the neck of the paroccipital process (character 64[2]); anteroposterior length of the centrum of dorsal vertebrae is greater than 110% of centrum height (character 132[0]); and presence of a longitudinal keel along ventral surface of dorsal vertebrae centra (character 133[1]).

The analysis after the removal of *Ahshislepelta* has a consistency index value of 0.507, and retention index of 0.739. Bootstrap values are low along the branches of Ankylosauridae (Supplemental Material S1; Fig. S9), with most nodes well below 50%; the highest bootstrap value within Ankylosauridae was for the clade including *Crichtonpelta benxiensis* (83%). Bremer support values were generally ≥3 across the base of the phylogeny (Supplemental Material S1; Fig. S9), with exception of the branch leading to the clade including *Emausaurus ernesti* (1). Bremer support values within Ankylosauridae are much lower, almost universally 1 or 2 (Supplemental Material S1; Fig. S9), with the exception of the branches leading to the taxa *Gobisaurus domoculus* (8), *Tsagantegia longicranialis* (4), and *Crichtonpelta benxiensis* (5). The low support values reflect the high level of homoplasy, and the small number of taxa with relatively complete specimens available for study.

**Figure 27:**
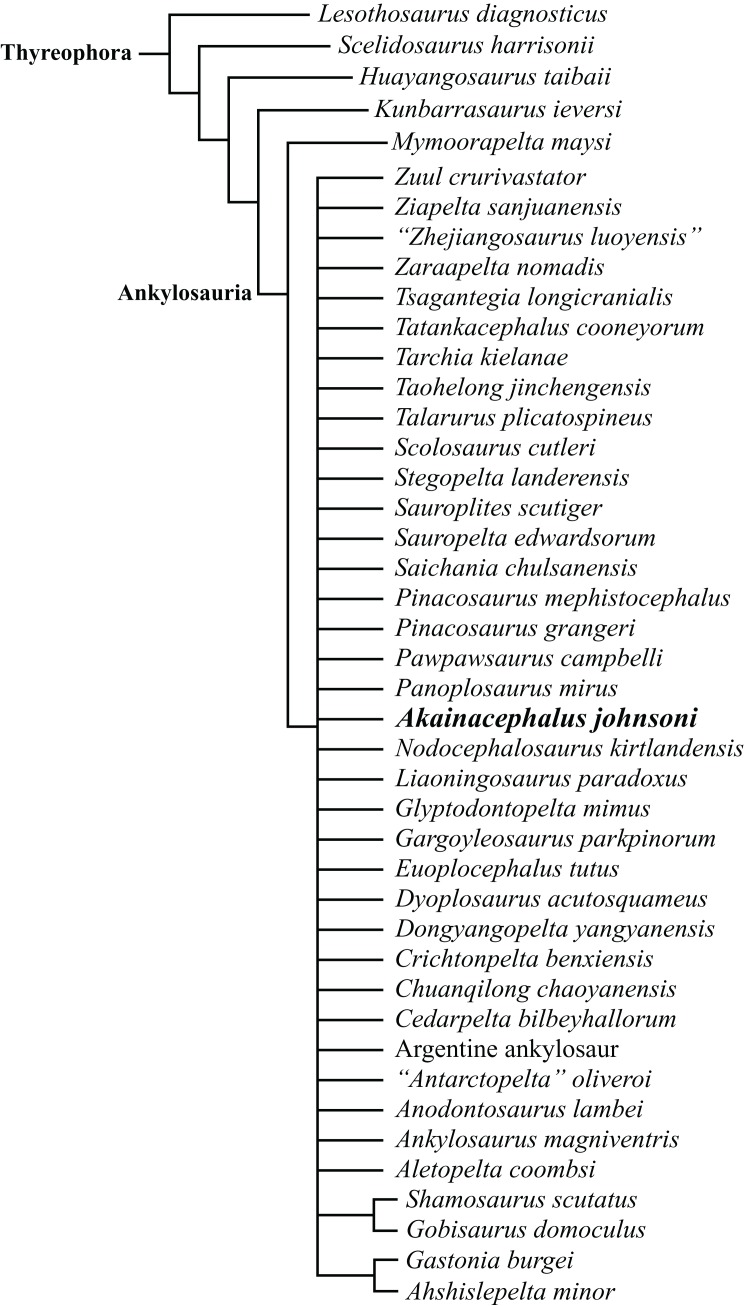
Phylogenetic results using character state codings from Arbour & Evans (2017). Phylogenetic analysis resulting in a strict consensus of 1990 equally most parsimonious trees and the phylogenetic position of *Akainacephalus johnsoni*, implemented into the character matrix from Arbour & Evans (2017). The resulting consensus tree renders *A. johnsoni* poorly resolved, consistently placing it within a large polytomy.

We also tested the phylogenetic position of *Akainacephalus johnsoni*, that is, in the character-taxon matrix of Arbour & Evans (2017), a modified version of the matrix from Arbour & Currie (2016). We analyzed these data in TNT using the same parameters as our original analysis. The resulting phylogeny recovered 1990 most parsimonious trees (length = 469), and the strict consensus tree nested *Akainacephalus johnsoni* in a large polytomy consisting of most ankylosaurid and nodosaurid taxa (Fig. 27). Calculating a maximum agreement subtree (as Arbour & Currie, 2016 did) recovers *Akainacephalus johnsoni* within the Ankylosauridae, which itself consists of three smaller polytomies (Supplemental Material S1; Fig. S10). Surprisingly, none of the phylogenetic trees recovered from our analysis of the Arbour & Evans (2017) character matrix resolved *Akainacephalus johnsoni* as a sister taxon to *Nodocephalosaurus*. Instead, *Nodocephalosaurus kirtlandensis* was consistently resolved as a sister taxon to *Talarurus plicatospineus*. Both *Akainacephalus johnsoni* and the clade *N. kirtlandensis* + *T. plicatospineus* are deeply nested with other Asian ankylosaurids (Supplemental Material S1; Fig. S10), rather than being resolved within the Ankylosaurini, which constsists almost exclusively of Late Cretaceous Laramidian ankylosaurids (Arbour & Currie, 2016; Arbour & Evans, 2017).

We refrain from making further interpretations of this analysis because the dataset appears to be extremely sensitive to taxon and character sampling. Though the strict consensus tree published by Arbour & Evans (2017) shows better resolution than the previous results, this is a highly-pruned dataset compared with the taxonomic sampling of the original Arbour & Currie (2016) analysis. When all taxa from the original dataset are included, the strict consensus tree from their analysis remains largely unresolved, with most taxa falling into three large polytomies (Fig. 27). These authors were only able to make further interpretations of ankylosaurian phylogeny by calculating a 50% majority rule consensus tree and a maximum agreement subtree. The former method, although useful for statistical exploration of the relative frequency of branching relationships in a set of trees, should not be used to make phylogenetic or evolutionary interpretations, because it is a biased summary of parsimonious trees, and thus can lead to potentially false precision, as elegantly discussed by Sumrall, Brochu & Merck, (2001). Maximum agreement subtrees do report phylogenetic structure common to all most parsimonious trees, but do so at the expense of many of the taxa in the original analysis. Furtheremore, when the strict consensus of an analysis including the full set of taxa is poorly resolved (as is the case with the Arbour and Currie matrix), the addition or exclusion of just one taxa can have a large effect on which taxa remain in the resulting maximum agreement subtree. In contrast, our primary analysis recovered considerably more phylogenetic structure in the strict consensus before removing any taxa (using either a priori or a posteriori methods), and thus did not require a technique such as a maximum agreement subtree to examine the overall structure of the tree. In any case, the low tree support for both our analysis and those of Arbour & Currie (2016) and Arbour & Evans (2017) indicate that much remains to be done in elucidating ankylosaurid phylogeny.

## Discussion

**Figure 28:**
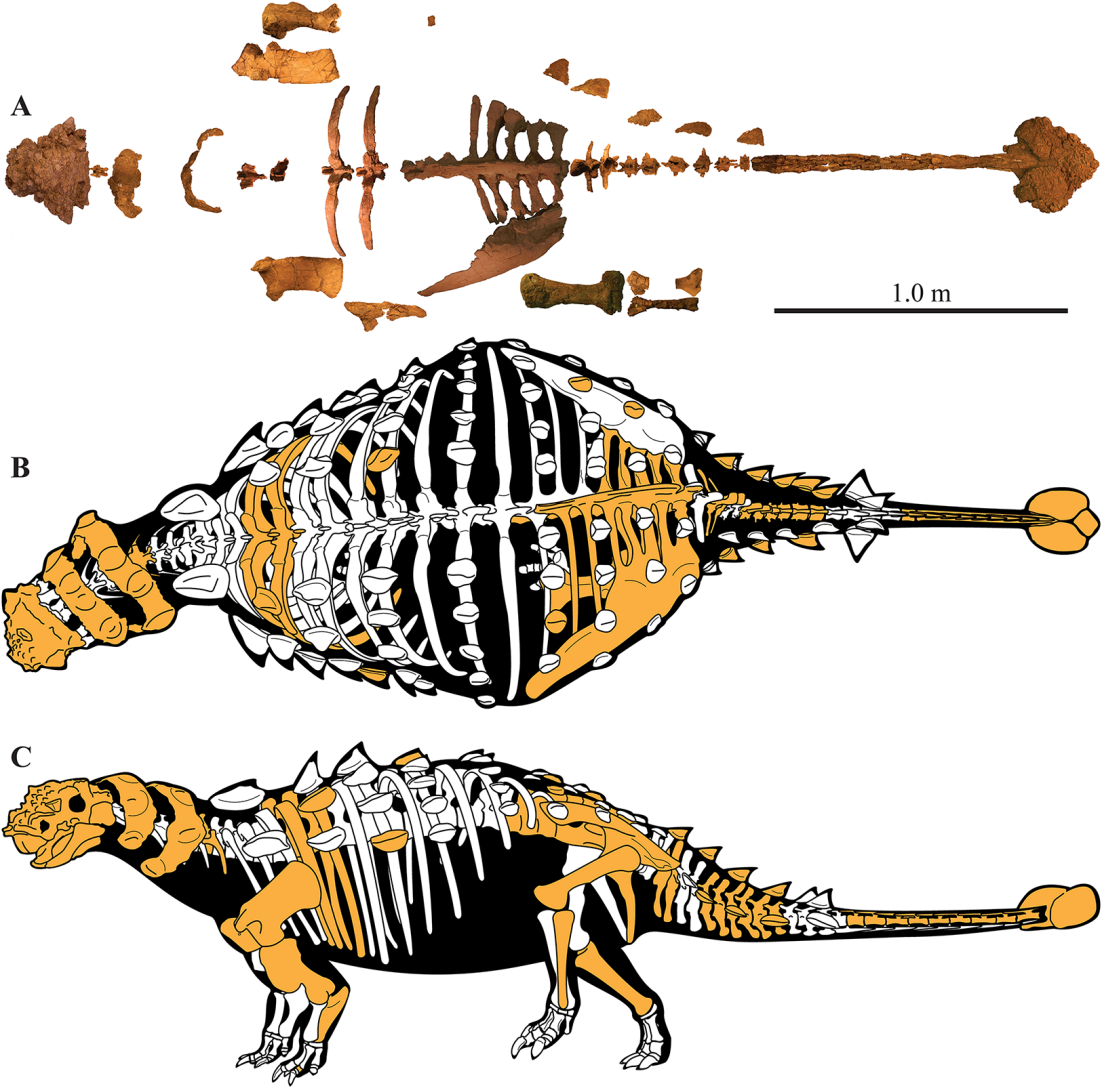
Preserved elements and skeletal reconstructions of *Akainacephalus johnsoni*. A composite showing all holotype skeletal material of *Akainacephalus johnson*i (UMNH VP 20202) anatomically arranged in dorsal view (A). Cartoon illustrating a full body reconstruction for *A. johnsoni* in (B), dorsal; and (C), left lateral view. Preserved material in the skeletal reconstructions is highlighted in orange.

The known taxonomic diversity of Late Cretaceous southern Laramidian akylosaurids continues to expand with the addition of *Akainacephalus johnsoni* (Fig. 28), a new taxon of ankylosaurid dinosaur from the upper Campanian Kaiparowits Formation of southern Utah. A suite of unique anatomical features including the characteristic sculpturing of cranial ornamentation and the unusually wide supraorbital horns, indicate that *Akainacephalus johnsoni* is distinct from other ankylosaurid specimen reported from the Kaiparowits Basin, including UMNH VP 21000, UMNH VP 19472, and UMNH VP 19473, as well as *Nodocephalosaurus kirtlandensis* (Sullivan, 1999), *Ahshislepelta minor* (Burns & Sullivan, 2011a), and *Ziapelta sanjuanensis* (Arbour et al., 2014) from the stratigraphically younger Kirtland Formation of the San Juan Basin in northwestern New Mexico.

*Akainacephalus johnsoni* and *Nodocephalosaurus kirtlandensis* (SMP VP-900) do share several similar anatomical features, such as the morphology of the cranial osteoderms and lateral orientation of the external nares (Fig. 8), suggesting a close taxonomic relationship (Fig. 26B). However, both taxa are temporally separated by nearly three million years; *Akainacephalus johnsoni* is recorded from the lower portion of the middle unit of the Kaiparowits Formation, which is late Campanian in age (76.26 ± 0.10 Ma (Roberts et al., 2013)), whereas *Nodocephalosaurus kirtlandensis* is from the upper-most Campanian-lower-most Maastrichtian (73.04 ± 0.25 Ma (Fassett & Steiner, 1997); 73.49 ± 0.25 Ma (Fowler, 2017)) De-na-zin Member of the Kirtland Formation. Such a large geochronologic gap alone renders it unlikely that *A. johnsoni* and *N. kirtlandensis* belong to the same species. Even igorning this age difference, a number of character states differ between the skulls of *Akainacephalus johnsoni* and *Nodocephalosaurus kirtlandensis* (SMP VP-900), including those in the basioccipital-basisphenoid region and amongst the cranial ornamentation, suggesting two separate taxa. The morphology of the basioccipital-basisphenoid complex in *Akainacephalus johnsoni* forms an anterior tapering triangle, whereas in *Nodocephalosaurus kirtlandensis* (Sullivan, 1999) it is anteroposteriorly oblong. Furthermore, *Nodocephalosaurus kirtlandensis* possesses a deeply concave (saddle-shaped in Sullivan, 1999) basioccipital in lateral view, and possesses a deeply piercing basioccipital foramen. *Akainacephalus johnsoni* possesses a ventrally concave basioccipital but it is shallower and lacks the basioccipital foramen (Fig. 4C). The quadratojugal horn in *N. kirtlandensis* is triangular and somewhat fin-shaped, whereas in *Akainacephalus johnsoni*, this element is more robust and is a ventrally projecting triangular protuberance. Although intraspecific variation in cranial ornamentation is present in ankylosaurid taxa, for example, *Euoplocephalus tutus*, *Anodontosaurus lambei*, *Pinacosaurus grangeri* (Penkalski, 2001; Arbour & Currie, 2013a), the size proportions and morphology of the quadratojugal horns between *Akainacephalus johnsoni* and *Nodocephalosaurus kirtlandensis* (SMP VP-900) (Figs. 8 and 9A–9D) differ significantly and are herein regarded as taxonomically distinct. The near vertical projection of the quadratojugal horns in *Akainacephalus johnsoni* are similar in orientation with the left quadratojugal horn preserved in *Nodocephalosaurus kirtlandensis* (SMP VP-900) but contrasts with the more laterally oriented quadratojugal horns observed in other ankylosaurids such as *Tarchia teresae* (Penkalski & Tumanova, 2017) (Figs. 7E and 7F), *M. ramachandrani* (INBR 21004) (Figs. 7G and 7H), *Saichania chulsanensis* (MPC 100/151), *Oohkotokia horneri* (MOR 433), *Euoplocephalus tutus* (AMNH FARB 5405, ROM 1930), *Ankylosaurus magniventris* (AMNH FARB 5214), and *Anodontosaurus lambei* (CMN 8530). Finally, the condylar end on the ventral surface of the quadrate is thickest medially in *Nodocephalosaurus kirtlandensis* (SMP VP-900), *Ankylosaurus magniventris* (AMNH FARB 5214), *Euoplocephalus tutus* (AMNH FARB 5337, AMH FARB 5405, ROM 1930), and *Anodontosaurus lambei* (TMP. 1997.132.1), whereas in *Akainacephalus johnsoni* the condylar contact appears to be situated along the middle of the ventral surface. *Akainacephalus johnsoni* and *Nodocephalosaurus kirtlandensis* are thus separate taxa, but they share morphological similarities that clearly distinguish them from other Late Cretaceous Laramidian ankylosaurids.

Because the southern Laramidian clade of *Akainacephalus johnsoni* + *Nodocephalosaurus kirtlandensis* is nested within Asian taxa, it suggests that this clade was a separate biogeographic dispersal event from Asia independent from the main radiation of Laramidian ankylosaurids (Ankylosaurini (Arbour & Currie, 2016)). Multiple intercontinental ankylosaurid dispersal patterns contribute to an already complex biogeographic pattern than was previously recognized for Late Cretaceous Laramidian terrestrial and freshwater vertebrate faunas (Gates et al., 2010; Lehman, 1997; Lehman, McDowell & Connelly, 2006; Loewen et al., 2013c; Sampson et al., 2004, 2010).

Geographic provincialism is present throughout western North America during the Campanian, as discussed by Kauffman (1984), Lehman (1997, 2001), and Gates et al. (2010). This resulted in many endemic terrestrial and freshwater vertebrate taxa (Sampson et al., 2010; Loewen et al., 2013c), and ankylosaurid dinosaurs (Arbour et al., 2014), including *Akainacephalus johnsoni*. The exact driving force behind provincialism within the Western Interior remains poorly understood to date, and evidence to support presence of physical geographical barriers appears absent. Latitudinal climatic gradients have been proposed by Gates et al. (2010). Such gradients produce different biomes, regional annual temperatures and quantities of meteoric water, causing physiological constraints on inhabiting organisms, preventing pandemic distributions of taxa. Climate gradient signatures are particularly well demonstrated within the plant fossil record of Campanian aged terrestrial environments throughout the Western Interior Basin (Miller et al., 2013). The dispersal of ankylosaurids into Laramidia appears to be approximately coeval (i.e., Campanian in age) with respect to the dispersal of other dinosaur clades, including tyrannosaurids (Thomson, Irmis & Loewen, 2013; Loewen et al., 2013c), ceratopsians (Sampson et al., 2010), and ornithopod dinosaurs (Ryan & Evans, 2005; Gates et al., 2013). These climate gradients, combined with fluctuations in sea level, may have helped reinforced Campanian provincialism (Loewen et al., 2013c).

The identification of several new ankylosaur taxa from southern Laramidian basins has resulted in a rapid taxonomic increase in diversity during the last 20 years. Currently, at least two diagnostic ankylosaurid taxa and potentially a third taxon have been identified from the Kirtland Formation of the San Juan Basin in northwestern New Mexico: *Nodocephalosaurus kirtlandensis* (Sullivan, 1999), *Ziapelta sanjuanensis* (Arbour et al., 2014), and *Ahshislepelta minor* (Burns & Sullivan, 2011a). Loewen et al. (2013a) assessed the different ankylosaur remains that were recognized and collected from the Dakota, Wahweap, and Kaiparowits formations, and other laterally contemporaneous formations within the GSENM area in southern Utah. Within this area, most ankylosaur material is known from the Kaiparowits Formation, which is largely due to the extensive expeditions that have been part of the Kaiparowits Basin Project that began in 2000, resulting in at least two distinct ankylosaurid taxa.

Although the taxonomic diversity of ankylosaurid material from southern Laramidian basins continues to expand, individual taxa remain largely underrepresented in terms of the number of specimens available for study, a phenomenon that appears to be characteristic for ankylosaurian dinosaurs as a whole, regardless of age or formation in which they are found. When relative abundance and species richness of ankylosaurian dinosaurs are quantitatively compared to the other coeval ornithischian dinosaur assemblages, (e.g., ornithopods and ceratopsians) (Brinkman, 1990; White, Fastovsky & Sheehan, 1998; Ryan & Evans, 2005; Horner, Goodwin & Myhrvold, 2011; Gates et al., 2013, Loewen et al., 2013b) from temporally equivalent stratigraphic horizons/formations, ankylosaurs are always rare and species-poor. For example, ankylosaur specimens recorded from the Kaiparowits Formation encompass <5% of associated dinosaur sites throughout the formation, whereas hadrosaurid and ceratopsian sites are at least an order of magnitude more abundant (Loewen et al., 2013a). When ankylosaurid material from northern Laramidian basins are compared to coeval dinosaur faunas from the Dinosaur Park and Hell Creek formations, ankylosaurid material is rarer by several orders of magnitude, even with the well-known taxon *Euoplocephalus tutus* and specimens referred to *Euoplocephalus* (White, Fastovsky & Sheehan, 1998; Ryan & Evans, 2005; Horner, Goodwin & Myhrvold, 2011; Gates et al., 2013; Loewen et al., 2013b). Thus, considerable future effort is required in Laramidian strata to discover additional taxa and specimens that will improve our understanding of ankylosaurid evolution and paleobiology. These recent finds from southern Laramidia, such as *Akainacephalus johnsoni*, demonstrate the strong potential for new discoveries, despite the overall rarity of ankylosaur fossils.

## Conclusion

*Akainacephalus johnsoni* (UMNH VP 20202) is a new taxon of ankylosaurid dinosaur from the upper Campanian Kaiparowits Formation of southern Utah, USA. It consists of a complete cranium and significant amount of diagnostic postcranial material that can be distinguished from all other known Late Cretaceous Laramidian ankylosaurids. *Akainacephalus johnsoni* is closely related to its stratigraphically younger sister taxon, *Nodocephalosaurus kirtlandensis* from the Kirtland Formation of New Mexico. Both taxa are more closely related to Asian ankylosaurids than they are to other Laramidian ankylosaurids. This suggests multiple ankylosaurid dispersal events from Asia to Laramidia during the Late Cretaceous. Lastly, together with *Dyoplosaurus acutosquameus* and *Scolosaurus cutleri* (∼77 Ma) from northern Laramidia, *Akainacephalus johnsoni* represents one of the older known ankylosaurid dinosaurs (∼76.3 Ma) from the Late Cretaceous of western North America.

## Supplemental Information

10.7717/peerj.5016/supp-1 Supplemental Information 1 Specimens and phylogenetic data.

10.7717/peerj.5016/supp-2 Supplemental Information 2 Character descriptions from Loewen and Kirkland in prep. used to score *Akainacephalus johnsoni*.

10.7717/peerj.5016/supp-3 Supplemental Information 3 Character matrix.

10.7717/peerj.5016/supp-4 Supplemental Information 4 Cranial and postcranial measurements *Akainacephalus johnsoni* (UMNH VP 20202).

## Additional Information and Declarations

### Data Availability

The following information was supplied regarding data availability: The raw data has been supplied as a Supplementary File.

### New Species Registration

The following information was supplied regarding the registration of a newly described species: Publication LSID: urn:lsid:zoobank.org:pub:42CB4F68-9E0D-42BB-943D-8A66E3D9DA12. Akainacephalus johnsoni urn:lsid:zoobank.org:act:11A47631-A9BE-4D58-9F6A-49E0897181AA.

## References

[ref-3] Arbour VM, Burns ME, Sissons RL (2009). A redescription of *Dyoplosaurus acutosquameus* Parks, 1924 (Ornithischia: Ankylosauria) and a revision of the genus. Journal of Vertebrate Paleontology.

[ref-4] Arbour VM, Burns ME, Sullivan RM, Lucas SG, Cantrell AK, Fry J, Suazo TL (2014). A new ankylosaurid dinosaur from the Upper Cretaceous (Kirtlandian) of New Mexico with implications for ankylosaurid diversity in the Upper Cretaceous of western North America. PLOS ONE.

[ref-6] Arbour VM, Currie PJ (2013a). *Euoplocephalus tutus* and the diversity of ankylosaurid dinosaurs in the Late Cretaceous of Alberta, Canada, and Montana, USA. PLOS ONE.

[ref-7] Arbour VM, Currie PJ (2013b). The taxonomic identity of a nearly complete ankylosaurid dinosaur skeleton from the Gobi Desert of Mongolia. Cretaceous Research.

[ref-8] Arbour VM, Currie PJ (2016). Systematics, phylogeny, and palaeobiogeography of the ankylosaurid dinosaurs. Journal of Systematic Palaeontology.

[ref-9] Arbour VM, Currie PJ, Badamgarav D (2014). The ankylosaurid dinosaurs of the Upper Cretaceous Barungoyot and Nemegt formations of Mongolia. Zoological Journal of the Linnean Society.

[ref-10] Arbour VM, Evans DC (2017). A new ankylosaurine dinosaur from the Judith River Formation of Montana, USA, based on an exceptional skeleton with soft tissue preservation. Royal Society Open Science.

[ref-11] Arbour VM, Lech-Hernes NL, Guldberg TE, Hurum JH, Currie PJ (2013). An ankylosaurid dinosaur from Mongolia with in situ armour and keratinous scale impressions. Acta Palaeontologica Polonica.

[ref-12] Arbour VM, Mallon JC (2017). Unusual cranial and postcranial anatomy in the archetypal ankylosaur *Ankylosaurus magniventris*. Facets.

[ref-14] Arbour VM, Zanno LE, Gates T (2016). Ankylosaurian dinosaur palaeoenvironmental associations were influenced by extirpation, sea-level fluctuation, and geodispersal. Palaeogeography Palaeoclimatology Palaeoecology.

[ref-15] Barrett PM, Hailu Y, Upchurch P, Burton AC (1998). A new ankylosaurian dinosaur (Ornithischia: Ankylosauria) from the Upper Cretaceous of Shanxi Province, People’s Republic of China. Journal of Vertebrate Paleontology.

[ref-16] Brinkman DB (1990). Palaeoecology of the Judith River Formation (Campanian) of Dinosaur Provincial Park, Alberta, Canada; Evidence from vertebrate microfossil localities. Palaeogeography Palaeoclimatology Palaeoecology.

[ref-17] Brown B (1908). The Ankylosauridae, a new family of armored dinosaurs from the Upper Cretaceous. Bulletin of the American Museum of Natural History.

[ref-18] Brown CM (2017). An exceptionally preserved armored dinosaur reveals the morphology and allometry of osteoderms and their horny epidermal coverings. PeerJ.

[ref-19] Brown CM, Henderson DM, Vinther J, Fletcher I, Sistiaga A, Herrera J, Summons RE (2017). An exceptionally preserved three-dimensional armored dinosaur reveals insights into coloration and Cretaceous predator-prey dynamics. Current Biology.

[ref-20] Burns ME (2008). Taxonomic utility of ankylosaur (Dinosauria: Ornithischia) osteoderms: *Glyptodontopelta mimus* Ford, 2000: a test case. Journal of Vertebrate Paleontology.

[ref-21] Burns ME, Currie PJ (2014). External and internal structure of ankylosaur (Dinosauria, Ornithischia) osteoderms and their systematic relevance. Journal of Vertebrate Paleontology.

[ref-22] Burns ME, Currie PJ, Sissons RL, Arbour VM (2011). Juvenile specimens of *Pinacosaurus grangeri* Gilmore, 1933 (Ornithischia: Ankylosauria) from the Late Cretaceous of China, with comments on the specific taxonomy of *Pinacosaurus*. Cretaceous Research.

[ref-23] Burns ME, Sullivan RM (2011a). A new ankylosaurid from the Upper Cretaceous Kirtland Formation, San Juan basin, with comments on the diversity of ankylosaurids in New Mexico. Fossil Record 3. New Mexico Museum of Natural History and Science Bulletin.

[ref-24] Burns ME, Sullivan RM (2011b). The tail club of *Nodocephalosaurus kirtlandensis* (Dinosauria: Ankylosauridae), with a review of ankylosaurid tail club morphology and homology. Fossil Record 3. New Mexico Museum of Natural History and Science Bulletin.

[ref-25] Carpenter K (1982). Skeletal and dermal armor reconstruction of *Euoplocephalus tutus* (Ornithischia: Ankylosauridae) from the Late Cretaceous Oldman Formation of Alberta. Canadian Journal of Earth Sciences.

[ref-26] Carpenter K, Farlow JO, Brett-Surman MK (1997). Ankylosaurs. The Complete Dinosaur.

[ref-27] Carpenter K, Carpenter K (2001). Phylogenetic analysis of the Ankylosauria. The Armored Dinosaurs.

[ref-28] Carpenter K (2004). Redescription of *Ankylosaurus magniventris* Brown, 1908 (Ankylosauridae) from the Upper Cretaceous of the Western Interior of North America. Canadian Journal of Earth Sciences.

[ref-29] Carpenter K, Bartlett J, Bird J, Barrick R (2008). Ankylosaurs from the Price River Quarries, Cedar Mountain Formation (Lower Cretaceous), east-central Utah. Journal of Vertebrate Paleontology.

[ref-30] Carpenter K, Hayashi S, Kobayashi Y, Maryańska T, Barsbold R, Sato K, Obata I (2011). *Saichania chulsanensis* (Ornithischia, Ankylsauridae) from the Upper Cretaceous of Mongolia. Palaeontographica Abteilung A.

[ref-31] Carpenter K, Kirkland JI, Lucas SG, Kirkland JI, Estep JW (1998). Review of the Lower and Middle Cretaceous ankylosaurs from North America. Lower and Middle Cretaceous Terrestrial Ecosystems. New Mexico Museum of Natural History and Science Bulletin.

[ref-32] Carpenter K, Kirkland JI, Burge DL, Bird J, Carpenter K (2001). Disarticulated skull of a new primitive ankylosaurid from the Lower Cretaceous of eastern Utah. The Armored Dinosaurs.

[ref-33] Carpenter K, Miles C, Cloward K (1998). Skull of a Jurassic ankylosaur (Dinosauria). Nature.

[ref-34] Colbert KH (1981). A primitive ornithischian dinosaur from the Kayenta Formation of Northern Arizona. Museum of Northern Arizona Press Bulletin Series.

[ref-35] Coombs WP (1971). The ankylosauria.

[ref-37] Coombs WP (1979). Osteology and myology of the hindlimb in the Ankylosauria (Reptillia, Ornithischia). Journal of Paleontology.

[ref-39] Crompton AW (1968). In search of the “insignificant”. Discovery.

[ref-40] Currie PJ, Badamgarav D, Koppelhus EB, Sissons EB, Vickaryous ME (2010). Hands, feet, and behavior in *Pinacosaurus grangeri* (Dinosauria: Ankylosauridae). Acta Palaeontologica Polonica.

[ref-41] Dong Z, Li X, Zhou S, Zhang Y (1977). On the stegosaurian remains from Zigong (Tzekung), Szechuan province. Vertebrata PalAsiatica.

[ref-42] Dong Z, Tang Z, Zhou S (1982). Note on the new Mid-Jurassic stegosaur from Sichuan Basin, China. Vertebrata PalAsiatica.

[ref-43] Eaton JG, Laurin J, Kirkland JI, Tibert NE, Leckie RM, Sageman BB, Goldstrand PM, Moore DW, Straub AW, Cobban WA, Dalebout JD (2001). Crtecaeous and Early Tertiary geology of Cedar and Parowan canyons, western Markagunt Plateau, Utah: Utah Geological Association Field Trip Road Log. Utah Geological Association Publication.

[ref-44] Fassett JE, Steiner MB (1997). Precise age of C33N-C32R magnetic polarity reversal, San Juan Basin, New Mexico and Colorado. New Mexico Geological Society Guidebook.

[ref-45] Ford TL (2000). A review of ankylosaur osteoderms from New Mexico and a preliminary review of ankylosaur armor. New Mexico Museum of Natural History and Science Bulletin.

[ref-46] Fowler DW (2017). Revised geochronology, correlation, and dinosaur stratigraphic ranges of the Santonian-Maastrichtian (Late Cretaceous) formations of the Western Interior of North America. PLOS ONE.

[ref-47] Galton PM (1978). Fabrosauridae, the basal family of ornithischian dinosaurs (Reptilia: Ornithopoda). Paläontologische Zeitschrift.

[ref-48] Gates TA, Lund EK, Boyd CA, DeBlieux DD, Titus AL, Evans DC, Getty MA, Kirkland JI, Eaton JG, Titus AL, Loewen MA (2013). Ornithopod dinosaurs from the Grand Staircase-Escalante National Monument region, Utah, and their role in paleobiogeographic and macroevolutionary studies. At the Top of the Grand Staircase: The Late Cretaceous of Southern Utah.

[ref-49] Gates TA, Sampson SD, Zanno LE, Roberts EM, Eaton JG, Nydam RL, Hutchison JH, Smith JA, Loewen MA, Getty MA (2010). Biogeography of terrestrial and freshwater vertebrates from the Late Cretaceous (Campanian) western interior of North America. Palaeogeography Palaeoclimatology Palaeoecology.

[ref-50] Gilmore CW (1933). Two new dinosaurian reptiles from Mongolia with notes on some fragmentary specimens. American Museum Novitates.

[ref-51] Godefroit P, Pereda-Superbiola XP, Li H, Zhi-Ming D (1999). A new species of the ankylosaurid dinosaur Pinacosaurus from the Late Cretaceous of Inner Mongolia (P.R. China). Bulletin de l’Institut Royal des Sciences Naturelles de Belgique, Sciences de la Terre.

[ref-52] Goloboff PA, Farris JS, Nixon KC (2008). TNT, a free program for phylogenetic analysis. Cladistics.

[ref-53] Haubold H (1990). Ein neuer Dinosaurier (Ornithischia, Thyreophora) aus dem Unteren Jura des nördlichen Mitteleuropa. Revue de Paleobiologie.

[ref-54] Hill RV, Witmer LM, Norell MA (2003). A new specimen of *Pinacosaurus grangeri* (Dinosauria: Ornithischia) from the Late Cretaceous of Mongolia: ontogeny and phylogeny of ankylosaurs. American Museum Novitates.

[ref-55] Horner JR, Goodwin MB, Myhrvold N (2011). Dinosaur census reveals abundant Tyrannosaurus and rare ontogenetic stages in the Upper Cretaceous Hell Creek Formation (Maastrichtian), Montana, USA. PLOS ONE.

[ref-56] Irmis RB, Hutchison JH, Sertich JJW, Titus AL, Titus AL, Loewen MA (2013). Crocodyliforms from the Late Cretaceous of Grand Staircase-Escalante National Monument and vicinity, southern Utah, U.S.A. At the Top of the Grand Staircase: The Late Cretaceous of Southern Utah.

[ref-57] Jinnah ZA, Titus AL, Loewen MA (2013). Tectonic and sedimentary controls, age, and correlatipon of the Upper Cretaceous Wahweap Formation, southern Utah. At the Top of the Grand Staircase: The Late Cretaceous of Southern Utah.

[ref-58] Jinnah ZA, Roberts EM, Deino AL, Larsen JS, Link PK, Fanning CM (2009). New 40Ar/39Ar and detrital zircon U-Pb ages for the Upper Cretaceous Wahweap and Kaiparowits formations in the Kaiparowits Plateau, Utah: implications for regional correlation, provenance, and biostratigraphy. Cretaceous Research.

[ref-59] Johnson KR, Nichols DJ, Hartman JH (2002). Hell Creek Formation: a 2001 synthesis. Geological Society of America Special Paper.

[ref-60] Kauffman EG, Westerman GEG (1984). Paleobiogeography and evolutionary response dynamics in the Cretaceous Western Interior seaway of North America. Jurassic-Cretaceous Biochronology and Paleogegraphy of North America: Geological Association of Canada Special Paper.

[ref-61] Kirkland JI (1998). A polacanthine ankylosaur (Ornithischia: Dinosauria) from the Early Cretaceous (Barremian) of Eastern Utah. New Mexico Museum of Natural History and Science Bulletin.

[ref-62] Kirkland JI, Alcalá L, Loewen MA, Espílez E, Mampel L, Wiersma JP (2013). The basal nodosaurid ankylosaur Europelta carbonensis n. gen., n. sp. from the Lower Cretaceous (lower Albian) Escucha Formation of northeastern Spain. PLOS ONE.

[ref-63] Kirkland JI, Carpenter K (1994). North America’s first pre-Cretaceous ankylosaur (Dinosauria) from the Upper Jurassic Morrison Formation of western Colorado. Brigham Young University Geology Studies.

[ref-64] Lambe LM (1902). New genera and species from the Belly River Series (mid-Cretaceous). Contributions to Canadian Palaeontology. Geological Survey of Canada.

[ref-66] Leahey LG, Molnar RE, Carpenter K, Witmer LM, Salisbury SW (2015). Cranial osteology of the ankylosaurian dinosaur formerly known as *Minmi* sp. (Ornithischia: Thyreophora) from the Lower Cretaceous Allaru Mudstone of Richmond, Queensland, Australia. PeerJ.

[ref-67] Lehman TM, Wolberg DL, Stump E, Rosenberg GD (1997). Late Campanian dinosaur biogeography in the western interior of North America. Dinofest International.

[ref-68] Lehman TM, Tanke DH, Carpenter K (2001). Late Cretaceous dinosaur provinciality. Mesozoic Vertebrate Life.

[ref-69] Lehman TM, McDowell FW, Connelly JN (2006). First isotopic (U-Pb) age for the Late Cretaceous Alamosaurus vertebrate fauna of west Texas, and its significance as a link between two faunal provinces. Journal of Vertebrate Paleontology.

[ref-70] Lively JR (2015). A new species of baenid turtle from the Kaiparowits Formation (Upper Cretaceous, Campanian) of southern Utah. Journal of Vertebrate Paleontology.

[ref-71] Loewen MA, Burns ME, Getty MA, Kirkland JI, Vickaryous MK, Titus AL, Loewen MA (2013a). Review of Late Cretaceous ankylosaurian dinosaurs from the Grand Staircase region, southern Utah. At the Top of the Grand Staircase: The Late Cretaceous of Southern Utah.

[ref-72] Loewen MA, Farke AA, Sampson SD, Getty MA, Lund EK, O’Connor PM, Titus AL, Loewen MA (2013b). Ceratopsid dinosaurs from the Grand Staircase of southern Utah. At the Top of the Grand Staircase: The Late Cretaceous of Southern Utah.

[ref-73] Loewen MA, Irmis RB, Sertich JJW, Currie PJ, Sampson SD (2013c). Tyrant dinosaur evolution tracks the rise and fall of Late Cretaceous oceans. PLOS ONE.

[ref-74] Loewen MA, Kirkland JI (2013). The evolution and biogeographic distribution of Ankylosaurisa: New insights from a comprehensive phylogenetic analysis. Journal of Vertebrate Paleontology, Program and Abstracts.

[ref-75] Junchang L, Qiang J, Yubo G, Zhixin L (2007). A new species of the ankylosaurid dinosaur *Crichtonsaurus* (Ankylosauridae: Ankylosauria) from the Cretaceous of Liaoning Province, China. Acta Geologica Sinica–English Edition.

[ref-76] Maddison WP, Maddison DR (2011). http://mesquiteproject.org.

[ref-77] Maleev EA (1952). A new ankylosaur from the Upper Cretaceous of Mongolia. Doklady Akademiia Nauk, SSSR.

[ref-78] Maleev EA (1954). The armored dinosaurs from the Cretaceous Period in Mongolia (Family Syrmosauridae). Doklady Akademiia Nauk, SSSR.

[ref-79] Maleev EA (1956). Armored dinosaurs of the Upper Cretaceous of Mongolia, Family Ankylosauridae. Trudy Palaeontologicheskoi Instytuta, Akademiia Nauk, SSSR.

[ref-80] Maryańska T (1969). Remains of armoured dinosaurs from the uppermost Cretaceous in Nemegt Basin, Gobi Desert. Palaeontologia Polonica.

[ref-81] Maryańska T (1977). Ankylosauridae (Dinosauria) from Mongolia. Palaeontologia Polonica.

[ref-82] Miles CA, Miles CJ (2009). Skull of *Minotaurasaurus ramachandrani*, a new Cretaceous ankylosaur from the Gobi Desert. Current Science.

[ref-83] Miller IM, Johnson KR, Kline DE, Nichols DJ, Barclay RS, Titus AL, Loewen MA (2013). A late Campanian flora from the Kaiparowits Formation, southern Utah, and a brief overview of the widely sampled but little-known Campanian vegetation of the western interior of North America. At the Top of the Grand Staircase: The Late Cretaceous of Southern Utah.

[ref-84] Molnar RE (1980). An ankylosaur (Ornithischia: Reptilia) from the Lower Cretaceous of southern Queensland. Memoirs of the Queensland Museum.

[ref-85] Nesbitt SJ (2011). The early evolution of archosaurs: relationships and the origin of major clades. Bulletin of the American Museum of Natural History.

[ref-86] Nopcsa F (1915). Die dinosaurier der Siebenbürgischen landesteile Ungarns [Dinosaurs of the Siebenberger regions of Hungary]. Mitteilungen aus dem Jahrbuche der Kgl. Ungarischen Geologischen Reichanstalt.

[ref-87] Nopcsa F (1928). Palaeontological notes on reptiles. Geologica Hungarica Series Palaeontologica.

[ref-88] Osborn HF (1923). Two Lower Cretaceous dinosaurs of Mongolia. American Museum Novitates.

[ref-89] Owen R (1842). Report on British fossil reptiles, part II. Report of the British Association for the Advancement of Science.

[ref-90] Owen R (1859). Palaeontology. Encyclopaedia Britannica.

[ref-124] Pang Q, Cheng Z (1998). A new ankylosaur of Late Cretaceous from Tianzhen, Shanxi. Progress in Natural Science.

[ref-91] Padian K, May CL, Lucas SG, Morales M (1993). The earliest dinosaurs. The Nonmarine Triassic. New Mexico Museum of Natural History & Science Bulletin.

[ref-92] Parks WA (1924). *Dyoplosaurus acutosquameus*, a new genus and species of armored dinosaur; and notes on a skeleton of *Prosaurolophus maximus*. University of Toronto Studies Geological Series.

[ref-93] Penkalski P, Carpenter K (2001). Variation in specimens referred to *Euoplocephalus tutus*. The Armored Dinosaurs.

[ref-94] Penkalski P (2014). A new ankylosaurid from the Late Cretaceous Two Medicine Formation of Montana, USA. Acta Palaeontologica Polonica.

[ref-95] Penkalski P, Blows WT (2013). *Scolosaurus cutleri* (Ornithischia: Ankylosauria) from the Upper Cretaceous Dinosaur Park Formation of Alberta, Canada. Canadian Journal of Earth Sciences.

[ref-125] Penkalski P, Tumanova T (2017). The cranial morphology and taxonomic status of *Tarchia* (Dinosauria: Ankylosauridae) from the Upper Cretaceous of Mongolia. Cretaceous Research.

[ref-96] Roberts EM (2005). Stratigraphic, taphonomic, and paleoenvironmental analysis for the Upper Cretaceous Kaiparowits Formation, Grand Staircase-Escalante National Monument, Utah.

[ref-97] Roberts EM (2007). Facies architecture and depositional environments of the Upper Cretaceous Kaiparowits Formation, southern Utah. Sedimentary Geology.

[ref-98] Roberts EM, Deino AL, Chan MA (2005). ^40^Ar/^39^Ar age of the Kaiparowits Formation, southern Utah, and correlation of contemporaneous Campanian strata and vertebrate faunas along the margin of the Western Interior Basin. Cretaceous Research.

[ref-99] Roberts EM, Sampson SD, Deino AL, Bowring SA, Buchwaldt R, Titus AL, Loewen MA (2013). The Kaiparowits Formation: a remarkable record of Late Cretaceous terrestrial environments, ecosystems, and evolution in western North America. At the Top of the Grand Staircase: The Late Cretaceous of Southern Utah.

[ref-100] Ryan MJ, Evans DC, Currie PJ, Koppelhus EB (2005). Ornithischian dinosaurs. Dinosaur Provincial Park A Spectacular Ancient Ecosystem Revealed.

[ref-101] Sampson SD, Loewen MA, Farke AA, Roberts EM, Forster CA, Smith JA, Titus AL (2010). New horned dinosaurs from Utah provide evidence for intracontinental dinosaur endemism. PLOS ONE.

[ref-102] Sampson S, Loewen M, Roberts E, Smith J, Zanno L, Gates T (2004). Provincialism in Late Cretaceous terrestrial faunas: new evidence from the Campanian Kaiparowits Formation of Utah. Journal of Vertebrate Paleontology Program and Abstracts.

[ref-103] Seeley HG (1887). On the classification of the fossil animals commonly named Dinosauria. Proceedings of the Royal Society of London.

[ref-104] Sereno PC (1986). Phylogeny of the bird-hipped dinosaurs (Order Ornithischia). National Geographic Research.

[ref-105] Sereno PC (1991). Lesothosaurus, “Fabrosaurids,” and the early evolution of Ornithischia. Journal of Vertebrate Paleontology.

[ref-106] Sereno PC (1998). A rationale for phylogenetic definitions, with applications to the higher-level taxonomy of Dinosauria. Neues Jahrbuch für Geologie und Paläontologie–Abhandlungen.

[ref-107] Sereno PC (1999). The evolution of dinosaurs. Science.

[ref-108] Sternberg CM (1929). A toothless armored dinosaur from the Upper Cretaceous of Alberta. Bulletin of the National Museum of Canada.

[ref-109] Sullivan RM (1999). *Nodocephalosaurus kirtlandensis* gen. et sp. nov., a new ankylosaurid dinosaur (Ornithischia: Ankylosauria) from the Upper Cretaceous (upper Campanian) Kirtland Formation of New Mexico. Journal of Vertebrate Paleontology.

[ref-126] Sullivan RM, Fowler DW, Lucas SG, Sullivan RM (2006). New specimens of the rare ankylosaurid dinosaur *Nodocephalosaurus kirtlandensis* (Ornithischia: Ankylosauridae) from the Upper Cretaceous Kirtland Formation (De-Na-Zin member), San Juan Basin, New Mexico. Late Cretaceous vertebrates from the Western Interior.

[ref-110] Sumrall CD, Brochu CA, Merck JW (2001). Global lability, regional resolution, and majority-rule consensus bias. Paleobiology.

[ref-111] Thompson RS, Parish JC, Maidment SCR, Barrett PM (2012). Phylogeny of the ankylosaurian dinosaurs (Ornithischia: Thyreophora). Journal of Systematic Palaeontology.

[ref-112] Thomson TJ, Irmis RB, Loewen MA (2013). First occurrence of a tyrannosaurid dinosaur from the Mesaverde Group (Neslen Formation) of Utah: implications for upper Campanian Laramidian biogeography. Cretaceous Research.

[ref-113] Thulborn RA (1971). Origins and evolution of ornithischian dinsosaurs. Nature.

[ref-127] Tumanova TA (1977). New data on the ankylosaur *Tarchia gigantea*. Paleontological Zhurnal.

[ref-114] Tumanova TA (1983). The first ankylosaur from the Lower Cretaceous of Mongolia. Sovmestnaya Sovetsko-Mongol’skaya Paleontogichekaya Ekpeditsiya. Trudy.

[ref-115] Tumanova TA (1987). The armored dinosaurs of Mongolia. Sovmestnaya Sovetsko-Mongol’skaya Paleontogichekaya Ekpeditsiya. Trudy.

[ref-116] Tumanova TA (1993). A new armored dinosaur from southeastern Gobi. Paleontologicheskii Zhurnal.

[ref-117] Vickaryous MK (2001). Skull morphology of the Ankylosauria.

[ref-118] Vickaryous MK, Maryańska T, Weishampel DB, Weishampel BD, Dodson P, Osmólska H (2004). Ankylosauria. The Dinosauria.

[ref-119] Vickaryous MK, Russell AP (2003). A redescription of the skull of *Euoplocephalus tutus* (Archosauria: Ornithischia): a foundationfor comparative and systematic studies of ankylosaurian dinosaurs. Zoological Journal of the Linnean Society.

[ref-120] Vickaryous MK, Russell AP, Currie PJ, Zhao XJ (2001). A new ankylosaurid (Dinosauria: Ankylosauria) from the Lower Cretaceous of China, with comments on ankylosaurian relationships. Canadian Journal of Earth Sciences.

[ref-121] Wiersma J, Irmis R (2013). A new ankylosaurid dinosaur (Ornithischia: Thyreophora) from the upper Campanian Kaiparowits Formation of Grand Staircase-Escalante National Monument, southern Utah. Journal of Vertebrate Paleontology Program and Abstracts.

[ref-122] White PD, Fastovsky DE, Sheehan PM (1998). Taphonomy and suggested structure of the dinosaurian assemblage of the Hell Creek Formation (Maastrichtian), eastern Montana and western North Dakota. Palaios.

[ref-123] Zanno LE, Loewen MA, Farke AA, Kim G-S, Claessens LPAM, McGarrity CT, Titus AL, Loewen MA (2013). Late Cretaceous theropod dinosaurs of southern Utah. At the Top of the Grand Staircase: The Late Cretaceous of Southern Utah.

