# Supplementary material for: A new southern Laramidian ankylosaurid, Akainacephalus johnsoni gen. et sp. nov., from the upper Campanian Kaiparowits Formation of southern Utah, USA
Source: PeerJ. 2018 Jul 19;6:e5016. doi: 10.7717/peerj.5016 (PMC6063217; doi:10.7717/peerj.5016)
Supplement: Supplemental Information 1 [file peerj-06-5016-s001.docx]

**Supplemental File S1.**

**A new southern Laramidian ankylosaurid *Akainacephalus johnsoni* gen. et sp. nov. from the upper Campanian Kaiparowits Formation of southern Utah**

Jelle P. Wiersma^1,2,3*^, Randall B. Irmis^2,3^

^1^Department of Geosciences, James Cook University, Townsville, Queensland, Australia

^2^Natural History Museum of Utah, Salt Lake City, Utah, United States of America; ^3^Department of Geology & Geophysics, University of Utah, Salt Lake City, Utah, United States of America

*Email: jelle.wiersma@my.jcu.edu.au

**Contents**

**Text S1.** Institutional abbreviations

**Table S1.** Examined specimens for comparison with *Akainacephalus johnsoni* (UMNH VP 20202)*.*

**Figure S1.** Strict Consensus tree from 21 equally most parsimonious phylogenetic trees, including the wildcard taxon *Ahshislepelta minor*.

**Figure S2.** Most parsimonious tree from the pruned dataset (*Ahshislepelta* removed), recovering *Akainacephalus johnsoni* as a sister taxon to *Nodocephalosaurus kirtlandensis*, with synapomorphy list.

**Figure S3.** Most parsimonious tree from the pruned dataset (*Ahshislepelta* removed), recovering *Akainacephalus johnsoni* as a sister taxon to *Nodocephalosaurus kirtlandensis*, with synapomorphy list.

**Figure S4.** Most parsimonious tree from the pruned dataset (*Ahshislepelta* removed), recovering *Akainacephalus johnsoni* as a sister taxon to *Nodocephalosaurus kirtlandensis*, with synapomorphy list.

**Figure S5.** Most parsimonious tree from the pruned dataset (*Ahshislepelta* removed), recovering *Akainacephalus johnsoni* as a sister taxon to *Nodocephalosaurus kirtlandensis*, with synapomorphy list.

**Figure S6.** Most parsimonious tree from the pruned dataset (*Ahshislepelta* removed), recovering *Akainacephalus johnsoni* as a sister taxon to *Nodocephalosaurus kirtlandensis*, with synapomorphy list.

**Figure S7.** Most parsimonious tree from the pruned dataset (*Ahshislepelta* removed), recovering *Akainacephalus johnsoni* as a sister taxon to *Nodocephalosaurus kirtlandensis*, with synapomorphy list.

**Figure S8.** Strict Consensus tree from 6 equally most parsimonious phylogenetic trees.

**Figure S9.** Branch support for the pruned phylogenetic analysis, superimposed on the strict consensus of six equally most parsimonious phylogenetic trees

**Figure S10.** 50% Majority Rule tree resulting from the Arbour and Currie (2016) and Arbour and Evans (2017) data matrices, used to determine an alternative phylogenetic position of *Akainacephalus johnsoni*

**Text S1. Institutional abbreviations.**

**Institutional Abbreviations: AMNH**, American Museum of Natural History, New York, New York, USA; **CMN**, Canadian Museum of Nature, Ottawa, Ontario, Canada; **INBR**, Victor Valley Museum, Apple Valley, California, USA; **MPC**, Paleontological Center, Mongolian Academy of Sciences, Ulanbataar, Mongolia; **UMNH**, Natural History Museum of Utah, Salt Lake City, Utah, USA; **NMMNH**, New Mexico Museum of Natural History and Science, Albuquerque, New Mexico, USA; **TMP** – Royal Tyrrell Museum of Paleontology, Drumheller, Alberta, Canada; **UALVP** – University of Alberta Laboratory for Vertebrate Paleontology, Edmonton, Alberta, Canada; **USNM**, Smithsonian National Museum of Natural History, Washington, DC, USA; **ROM**, Royal Ontario Museum, Toronto, Ontario, Canada; **SMP**, State Museum of Pennsylvania, Harrisburg, Pennsylvania, USA; **ZPAL**, Zaklad Paleobiologii, Polish Academy of Sciences, Warsaw, Poland.

**Table S1. Examined specimens for comparison with *Akainacephalus johnsoni* (UMNH VP 20202)*.***

**
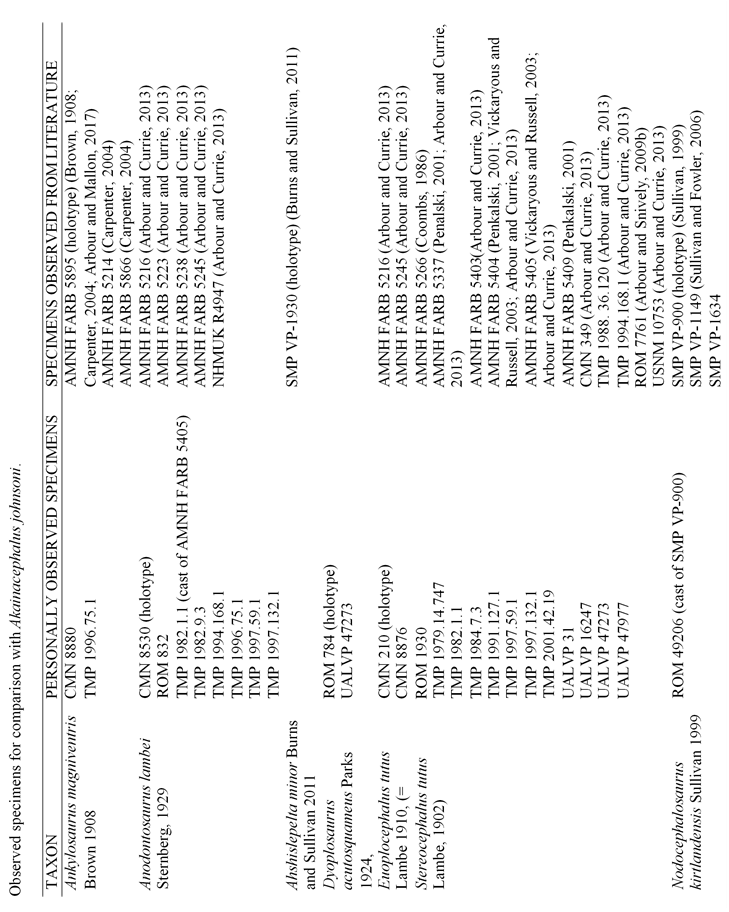
**

**
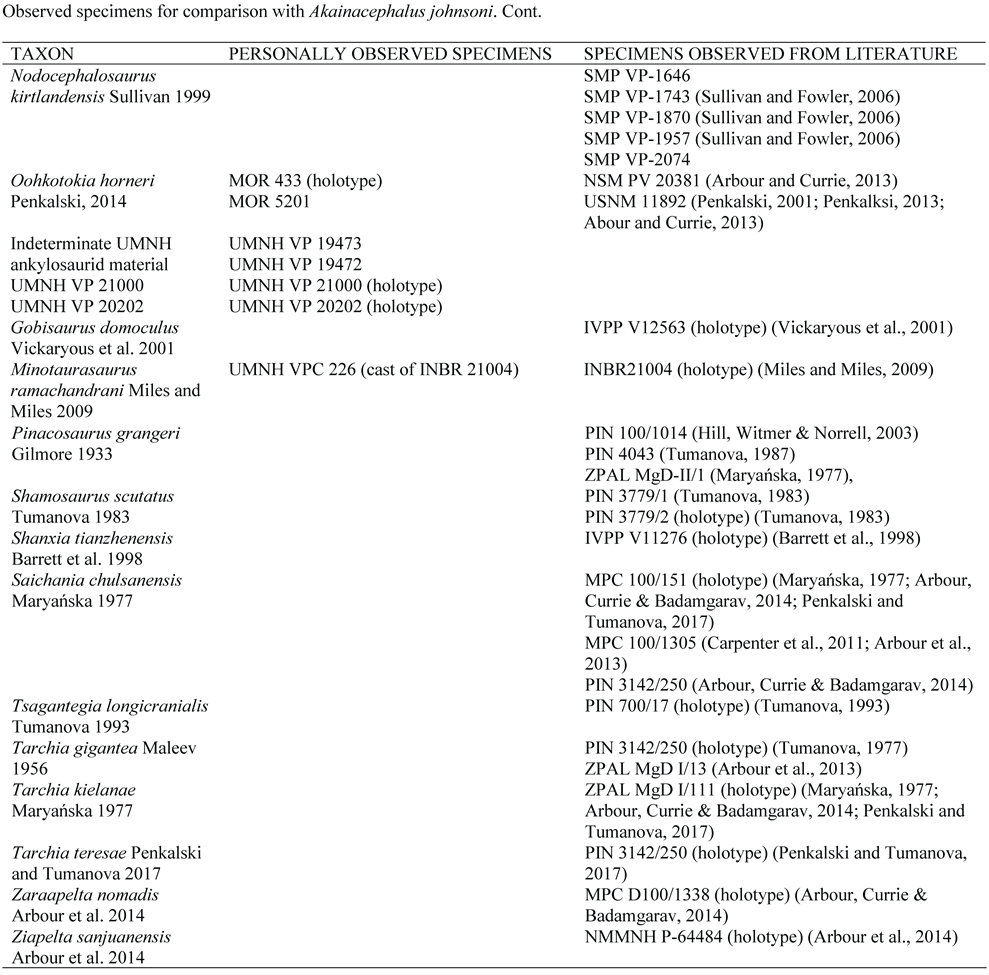
**


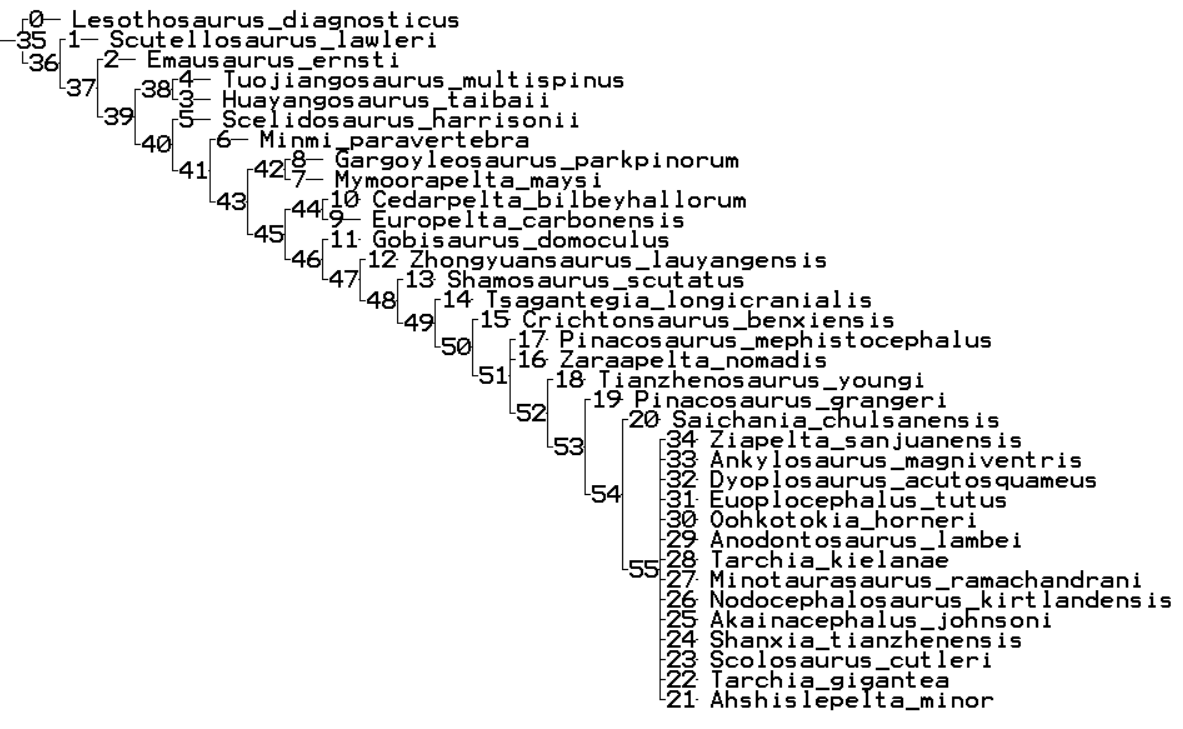


**Figure S1.** Strict Consensus tree from 21 equally most parsimonious phylogenetic trees, including the wildcard taxon *Ahshislepelta minor*.


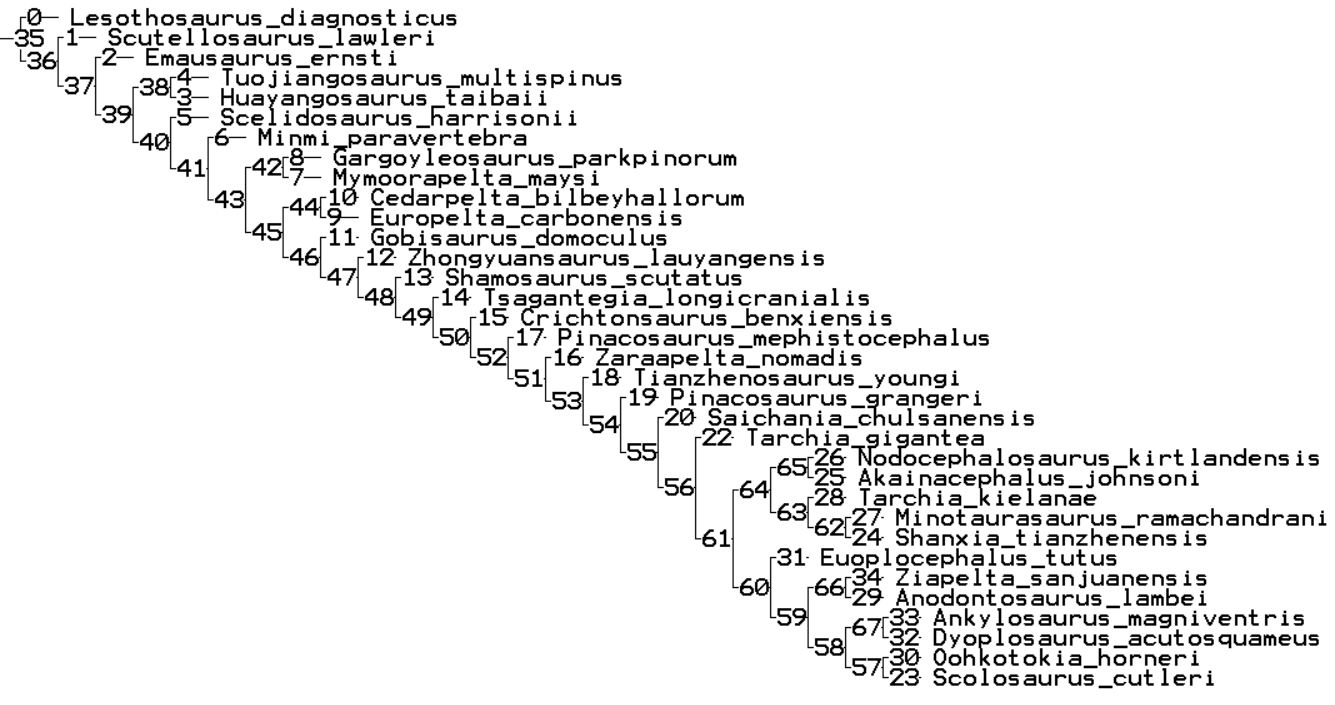


**Figure S2.** Most parsimonious tree from the pruned dataset (*Ahshislepelta* removed), recovering *Akainacephalus johnsoni* as a sister taxon to *Nodocephalosaurus kirtlandensis*, with synapomorphy list.

**Node 37:** No synapomorphies

**Node 38:** 84(1)

**Node 39:** 3(3), 60(1), 72(1), 98(1), 134(1), 145(2), 169(1), 177(1), 179(1), 189(1), 193(1), 196(1), 201(0), 280(1), 281(1), 284(1), 286(0), 288(0)

**Node 40:** 37(1), 97(1), 119(1), 138(1)

**Node 41:** 40(1), 42(1), 82(1), 88(1), 90(1), 92(1), 93(1), 101(1), 120(1), 153(1), 161(1), 162(1), 181(1), 192(1), 199(1), 200(1), 206(1), 219(1), 229(1)

**Node 42:** 1(1), 15(1), 25(1), 30(2), 32(1), 49(2), 61(1), 100(1)

**Node 43:** 51(1), 52(1), 104(1), 115(1), 128(1), 129(1), 152(2), 177(1), 254(1), 260(2)

**Node 44:** 72(1), 98(1), 99(1), 101(2), 122(1), 179(1), 180(1), 196(1), 212(2), 213(1), 230(1), 232(1)

**Node 45:** 6(1), 14(1), 74(1), 144(1), 148(2), 212(1)

**Node 46:** 3(1), 28(1), 44(0), 46(0), 49(1), 50(1), 58(1), 59(1), 73(1), 75(1), 124(1), 132(1), 137(1), 142(1), 158(1), 172(1), 183(1), 188(1), 189(1), 195(1), 197(1), 198(1), 201(2), 220(1), 224(1)

**Node 47:** 9(1), 12(1), 20(2), 23(1), 31(1), 34(1), 41(1), 48(1), 53(1), 56(1), 61(0), 94(1), 119(2)

**Node 48:** 10(1), 64(1), 111(1)

**Node 49:** 3(0), 134(1)

**Node 50:** 17(0), 21(1), 80(0), 102(1), 103(2), 104(1)

**Node 51:** 2(1), 4(0), 43(1), 44(1), 45(1), 48(3), 49(2), 56(0), 57(1), 63(1), 67(1), 70(1)

**Node 52:** 1(3), 60(1), 104(2)

**Node 53:** No synapomorphies

**Node 54:** 35(1), 54(1), 103(3), 104(3)

**Node 55:** 4(1), 55(1)

**Node 56:** 11(1), 129(1), 202(1), 203(1), 204(1), 228(1)

**Node 57:** 168(1), 174(1), 177(1)

**Node 58:** 227(2)

**Node 59:** 233(1), 238(1)

**Node 60:** 109(0), 210(1)

**Node 61:** 1(2), 2(2), 67(0), 103(1), 104(1), 111(2), 140(1), 145(2)

**Node 62:** 5(1), 55(0), 64(2), 121(1), 262(0)

**Node 63:** No synapomorphies

**Node 64:** 70(0), 118(1)

**Node 65:** 132(0), 133(1)

**Node 66:** 4(0), 10(0), 41(0), 46(1), 57(0)

**Node 67:** 250(1)

**Node 68:** 39(0)**,** 64(1)

**Node 69:** 211(1)**,** 262(1)


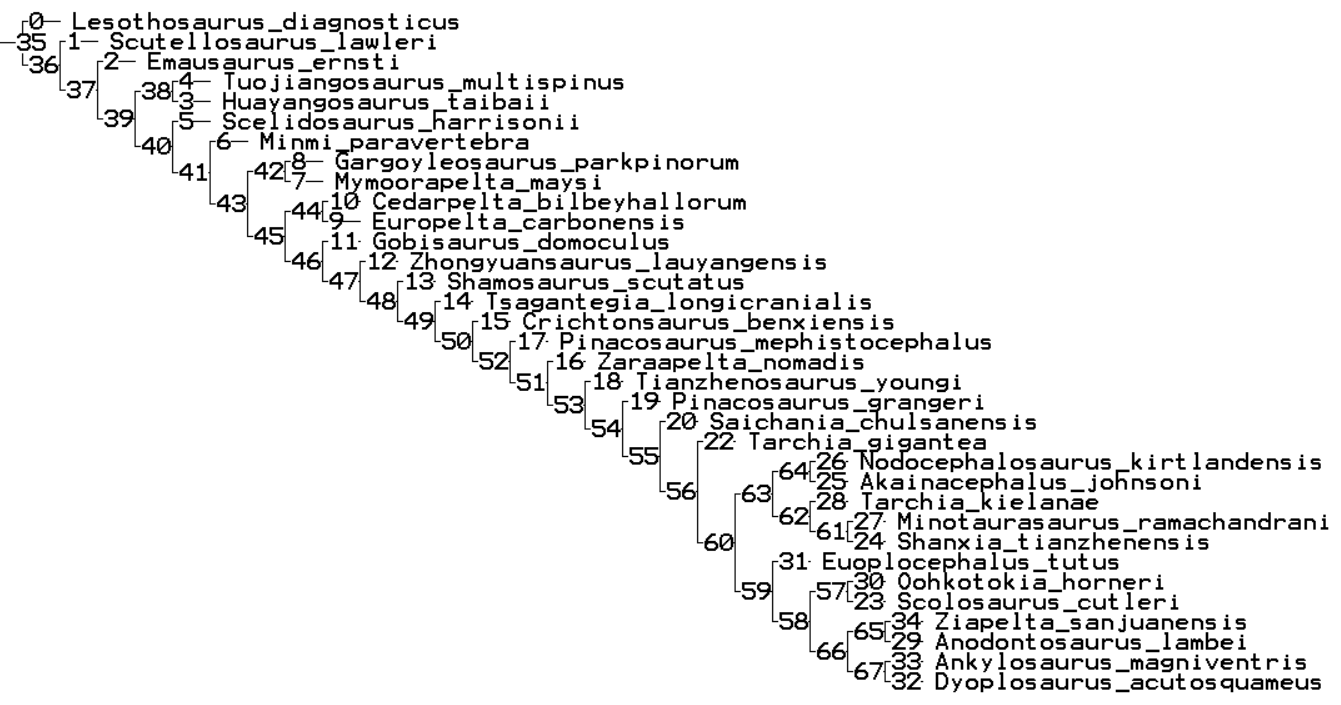


**Figure S3.** Most parsimonious tree from the pruned dataset (*Ahshislepelta* removed), recovering *Akainacephalus johnsoni* as a sister taxon to *Nodocephalosaurus kirtlandensis*, with synapomorphy list.

**Node 37:** No synapomorphies

**Node 38:** 84(1)

**Node 39:** 3(3), 60(1), 72(1), 98(1), 134(1), 145(2), 169(1), 177(1), 179(1), 189(1), 193(1), 196(1), 201(0), 280(1), 281(1), 284(1), 286(0), 288(0)

**Node 40:** 37(1), 97(1), 119(1), 138(1)

**Node 41:** 40(1), 42(1), 82(1), 88(1), 90(1), 92(1), 93(1), 101(1), 120(1), 153(1), 161(1), 162(1), 181(1), 192(1), 199(1), 200(1), 206(1), 219(1), 229(1)

**Node 42:** 1(1), 15(1), 25(1), 30(2), 32(1), 49(2), 61(1), 100(1)

**Node 43:** 51(1), 52(1), 104(1), 115(1), 128(1), 129(1), 152(2), 177(1), 254(1), 260(2)

**Node 44:** 72(1), 98(1), 99(1), 101(2), 122(1), 179(1), 180(1), 196(1), 212(2), 213(1), 230(1), 232(1)

**Node 45:** 6(1), 14(1), 74(1), 144(1), 148(2), 212(1)

**Node 46:** 3(1), 28(1), 44(0), 46(0), 49(1), 50(1), 58(1), 59(1), 73(1), 75(1), 124(1), 132(1), 137(1), 142(1), 158(1), 172(1), 183(1), 188(1), 189(1), 195(1), 197(1), 198(1), 201(2), 220(1), 224(1)

**Node 47:** 9(1), 12(1), 20(2), 23(1), 31(1), 34(1), 41(1), 48(1), 53(1), 56(1), 61(0), 94(1), 119(2)

**Node 48:** 10(1), 64(1), 111(1)

**Node 49:** 3(0), 134(1)

**Node 50:** 17(1), 21(1), 80(0), 102(1), 103(2), 104(1)

**Node 51:** 2(1), 4(0), 43(1), 44(1), 4(1), 48(3), 49(2), 56(0), 57(1), 63(1), 67(1), 70(1)

**Node 52:** 1(3), 60(1), 104(2)

**Node 53:** No synapomorphies

**Node 54:** 35(1), 54(1), 103(3), 104(3)

**Node 55:** 4(1), 55(1)

**Node 56:** 11(1), 129(1), 202(1), 203(1), 204(1), 228(1)

**Node 57:** 227(1)

**Node 58:** 211(1), 262(1)

**Node 59:** 39(0), 64(1)

**Node 60:** 109(0), 170(1), 210(1)

**Node 61:** 1(3), 2(2), 67(0), 103(1), 104(1), 111(2), 140(1), 145(2)

**Node 62:** 5(1), 55(0), 64(2), 121(1), 262(0)

**Node 63:** 227(2)

**Node 64:** 233(1), 238(1)

**Node 65:** No synapomorphies

**Node 66:** 70(0), 118(1)

**Node 67:** 132(0), 133(1)

**Node 68:** 4(0), 10(0), 41(0), 46(1), 57(0)

**Node 69:** 250(1)


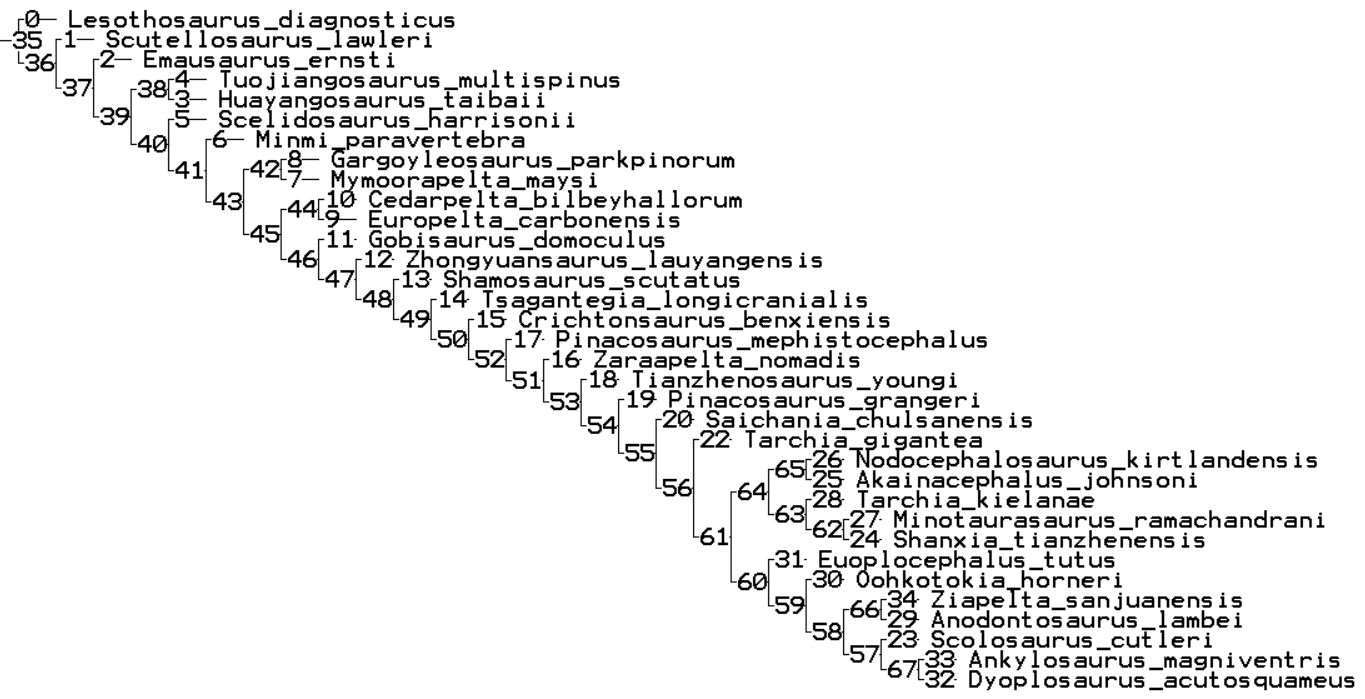


**Figure S4.** Most parsimonious tree from the pruned dataset (*Ahshislepelta* removed), recovering *Akainacephalus johnsoni* as a sister taxon to *Nodocephalosaurus kirtlandensis*, with synapomorphy list.

**Node37:** No synapomorphies

**Node38:** 84(1)

**Node39:** 3(3), 60(1), 72(1), 98(1), 134(1), 145(2), 169(1), 177(1), 179(1), 189(1), 193(1), 196(1), 201(0), 280(1), 281(1), 284(1), 286(0), 288(0)

**Node40:** 37(1), 97(1), 119(1), 138(1)

**Node41:** 40(1), 42(1), 82(1), 88(1), 90(1), 92(1), 93(1), 101(1), 120(1), 153(1), 161(1), 162(1), 181(1), 192(1), 199(1), 200(1), 206(1), 219(1), 229(1)

**Node42:** 1(1), 15(1), 25(1), 30(2), 32(1), 49(2), 61(1), 100(1)

**Node43:** 51(1), 52(1), 104(1), 115(1), 128(1), 129(1), 152(2), 177(1), 254(1), 260(2)

**Node44:** 72(1), 98(1), 99(1), 101(2), 122(1), 179(1), 180(1), 196(1), 212(2), 213(1), 230(1), 232(1)

**Node45:** 6(1), 14(1), 74(1), 144(1), 148(2), 212(1)

**Node46:** 3(1), 28(1), 44(0), 46(0), 49(1), 50(1), 58(1), 59(1), 73(1), 75(1), 124(1), 132(1), 137(1), 142(1), 158(1), 172(1), 183(1), 188(1), 189(1), 195(1), 197(1), 198(1), 201(2), 220(1), 224(1)

**Node47:** 9(1), 12(1), 20(2), 23(1), 31(1), 34(1), 41(1), 48(1), 53(1), 56(1), 61(0), 94(1), 119(2)

**Node48:** 10(1), 64(1), 111(1)

**Node49:** 3(0), 134(1)

**Node50:** 17(1), 21(1), 80(0), 102(1), 103(2), 104(1)

**Node51:** 2(1), 4(0), 43(1), 44(1), 45(1), 48(3), 49(2), 56(0), 57(1), 63(1), 67(1), 70(1)

**Node52:** 1(3), 60(1), 104(2)

**Node53:** No synapomorphies

**Node54:** 35(1), 54(1), 103(3), 104(3)

**Node55:** 4(1), 55(1)

**Node56:** 11(1), 129(1), 202(1), 203(1), 204(1), 228(1)

**Node57:** 168(1), 174(1), 177(1)

**Node58:** 227(2)

**Node59:** 189(0), 191(0)

**Node60:** 39(0), 64(1)

**Node61:** 109(0)

**Node62:** 1(2), 2(2), 67(0), 103(1), 104(1), 111(2), 140(1), 145(2)

**Node63:** 5(1), 55(0), 64(2), 121(1), 262(0)

**Node64:** No synapomorphies

**Node65:** 70(0), 118(1)

**Node66:** 132(0), 133(1)

**Node67:** 4(0), 10(0), 41(0), 46(1), 57(0)

**Node68:** 250(1)

**Node69:** 211(1), 262(1)


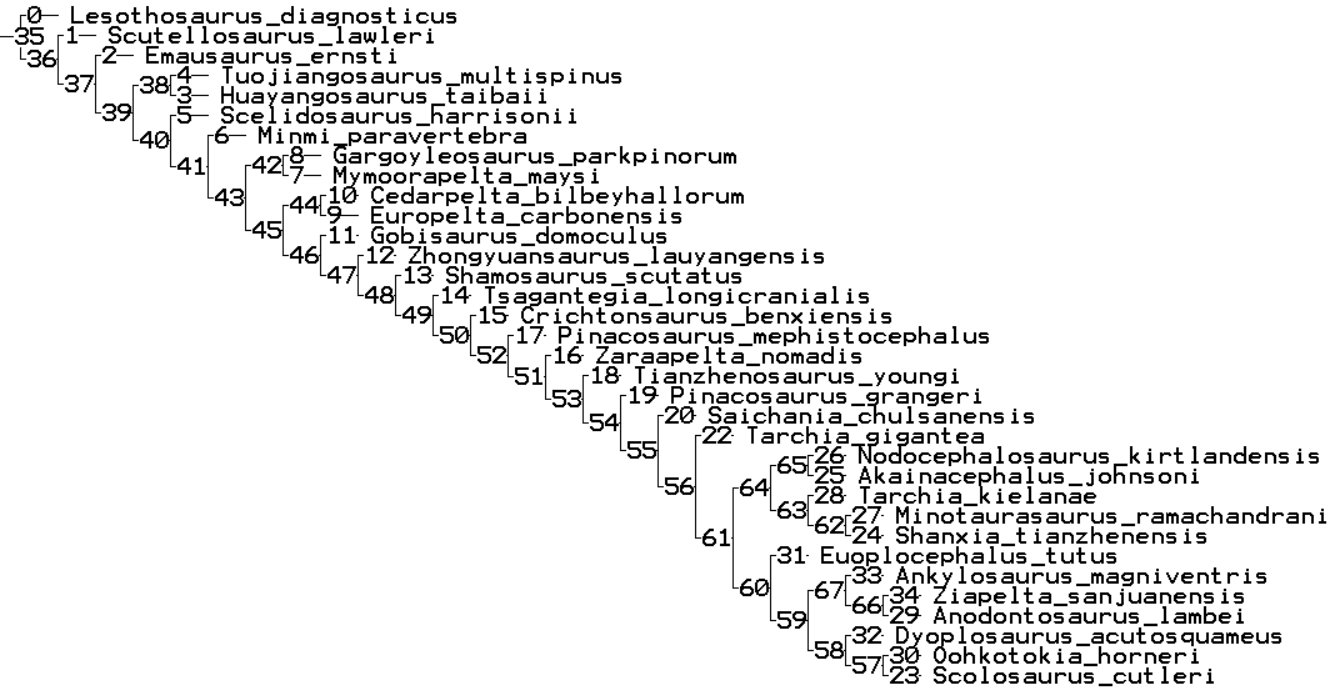


**Figure S5.** Most parsimonious tree from the pruned dataset (*Ahshislepelta* removed), recovering *Akainacephalus johnsoni* as a sister taxon to *Nodocephalosaurus kirtlandensis*, with synapomorphy list.

**Node 37:** No synapomorphies

**Node 38:** 84(1)

**Node 39:** 3(3), 60(1), 72(1), 98(1), 134(1), 145(2), 169(1), 177(1), 179(1), 189(1), 193(1), 196(1), 201(0), 280(1), 281(1), 284(1), 286(0), 288(0)

**Node 40:** 37(1), 97(1), 119(1), 138(1)

**Node 41:** 40(1), 42(1), 82(1), 88(1), 90(1), 92(1), 93(1), 101(1), 120(1), 153(1), 161(1), 162(1), 181(1), 192(1), 199(1), 200(1), 206(1), 219(1), 229(1)

**Node 42:** 1(1), 15(1), 25(1), 30(2), 32(1), 49(2), 61(1), 100(1)

**Node 43:** 51(1), 52(1), 104(1), 115(1), 128(1), 129(1), 152(2), 177(1), 254(1), 260(2)

**Node 44:** 72(1), 98(1), 99(1), 101(2), 122(1), 179(1), 180(1), 196(1), 212(2), 213(1), 230(1), 232(1)

**Node 45:** 6(1), 14(1), 74(1), 144(1), 148(2), 212(1)

**Node 46:** 3(1), 28(1), 44(0), 46(0), 49(1), 50(1), 58(1), 59(1), 73(1), 75(1), 124(1), 132(1), 137(1), 142(1), 158(1), 172(1), 183(1), 188(1), 189(1), 195(1), 197(1), 198(1), 201(2), 220(1), 224(1)

**Node 47:** 9(1), 12(1), 20(2), 23(1), 31(1), 34(1), 41(1), 48(1), 53(1), 56(1), 61(0), 94(1), 119(2)

**Node 48:** 10(1), 64(1), 111(1)

**Node 49:** 3(0), 134(1)

**Node 50:** 17(1), 21(1), 80(0), 102(1), 103(2), 104(1)

**Node 51:** 2(1), 4(0), 43(1), 44(1), 45(1), 48(3), 49(2), 56(0), 57(1), 63(1), 67(1), 70(1)

**Node 52:** 1(3), 60(1), 104(2)

**Node 53:** No synapomorphies

**Node 54:** 35(1), 54(1), 103(3), 104(3)

**Node 55:** 4(1), 55(1)

**Node 56:** 11(1), 129(1), 202(1), 203(1), 204(1), 228(1)

**Node 57:** 168(1), 174(1), 177(1)

**Node 58:** 227(2)

**Node 59:** No synapomorphies

**Node 60:** 189(0), 191(0)

**Node 61:** 109(0), 210(1)

**Node 62:** 1(2), 2(2), 67(0), 103(1), 104(1), 111(2), 140(1), 145(2)

**Node 63:** 5(1), 55(0), 64(2), 121(1), 262(0)

**Node 64:** No synapomorphies

**Node 65:** 70(0), 118(1)

**Node 66:** 132(0), 133(1)

**Node 67:** 4(0), 10(0), 41(0), 46(1), 57(0)

**Node 68:** 250(1)

**Node 69:** 39(0), 64(1)


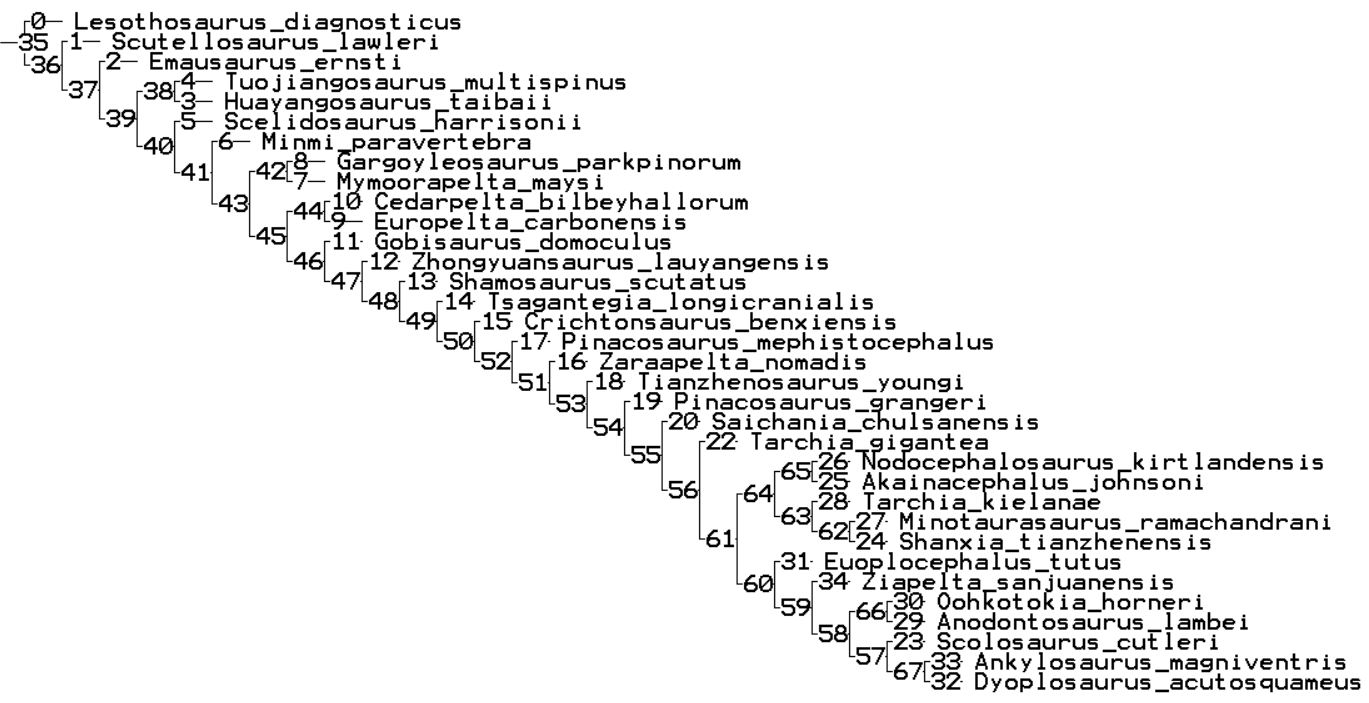


**Figure S6.** Most parsimonious tree from the pruned dataset (*Ahshislepelta* removed), recovering *Akainacephalus johnsoni* as a sister taxon to *Nodocephalosaurus kirtlandensis*, with synapomorphy list.

**Node 37:** No synapomorphies

**Node 38:** 84(1)

**Node 39:** 3(3), 60(1), 72(1), 98(1), 134(1), 145(2), 169(1), 177(1), 179(1), 189(1), 193(1), 196(1), 201(0), 280(1), 281(1), 284(1), 286(0), 288(0)

**Node 40:** 37(1), 97(1), 119(1), 138(1)

**Node 41:** 40(1), 42(1), 82(1), 88(1), 90(1), 92(1), 93(1), 101(1), 120(1), 153(1), 161(1), 162(1), 181(1), 192(1), 199(1), 200(1), 206(1), 219(1), 229(1)

**Node 42:** 1(1), 15(1), 25(1), 30(2), 32(1), 49(2), 61(1), 100(1),

**Node 43:** 51(1), 52(1), 104(1), 115(1), 128(1), 129(1), 152(2), 177(1), 254(1), 260(2),

**Node 44:** 72(1), 98(1), 99(1), 101(2), 122(1), 179(1), 180(1), 196(1), 212(2), 213(1), 230(1), 232(1)

**Node 45:** 6(1), 14(1), 74(1), 144(1), 148(2), 212(1)

**Node 46:** 3(1), 28(1), 44(0), 46(0), 49(1), 50(1), 58(1), 59(1), 73(1), 75(1), 124(1), 132(1), 137(1), 142(1), 158(1), 172(1), 183(1), 188(1), 189(1), 195(1), 197(1), 198(1), 201(2), 220(1), 224(1)

**Node 47:** 9(1), 12(1), 20(2), 23(1), 31(1), 34(1), 41(1), 48(1), 53(1), 56(1), 61(0), 94(1), 119(2)

**Node 48:** 10(1), 64(1), 111(1)

**Node 49:** 3(0), 134(1)

**Node 50:** 17(1), 21(1), 80(0), 102(1), 103(2), 104(1)

**Node 51:** 2(1), 4(0), 43(1), 44(1), 45(1), 48(3), 49(2), 56(0), 57(1), 63(1), 67(1), 70(1)

**Node 52:** 1(3), 60(1), 104(2)

**Node 53:** No synapomorphies

**Node 54:** 35(1), 54(1), 103(3), 104(3)

**Node 55:** 4(1), 55(1)

**Node 56:** 11(1), 129(1), 202(1), 203(1), 204(1), 228(1)

**Node 57:** 168(1), 174(1), 177(1)

**Node 58:** 227(2)

**Node 59:** 238(1)

**Node 60:** 189(0), 191(0)

**Node 61:** 109(0), 210(1)

**Node 62:** 1(2), 2(2), 67(0), 103(1), 104(1), 111(2), 140(1), 145(2)

**Node 63:** 5(1), 55(0), 121(1), 262(0)

**Node 64:** No synapomorphies

**Node 65:** 70(0), 118(1)

**Node 66:** 132(0), 133(1)

**Node 67:** 4(0), 10(0), 41(0), 46(1), 57(0)

**Node 68:** 250(1)

**Node 69:** 211(1), 262(1)


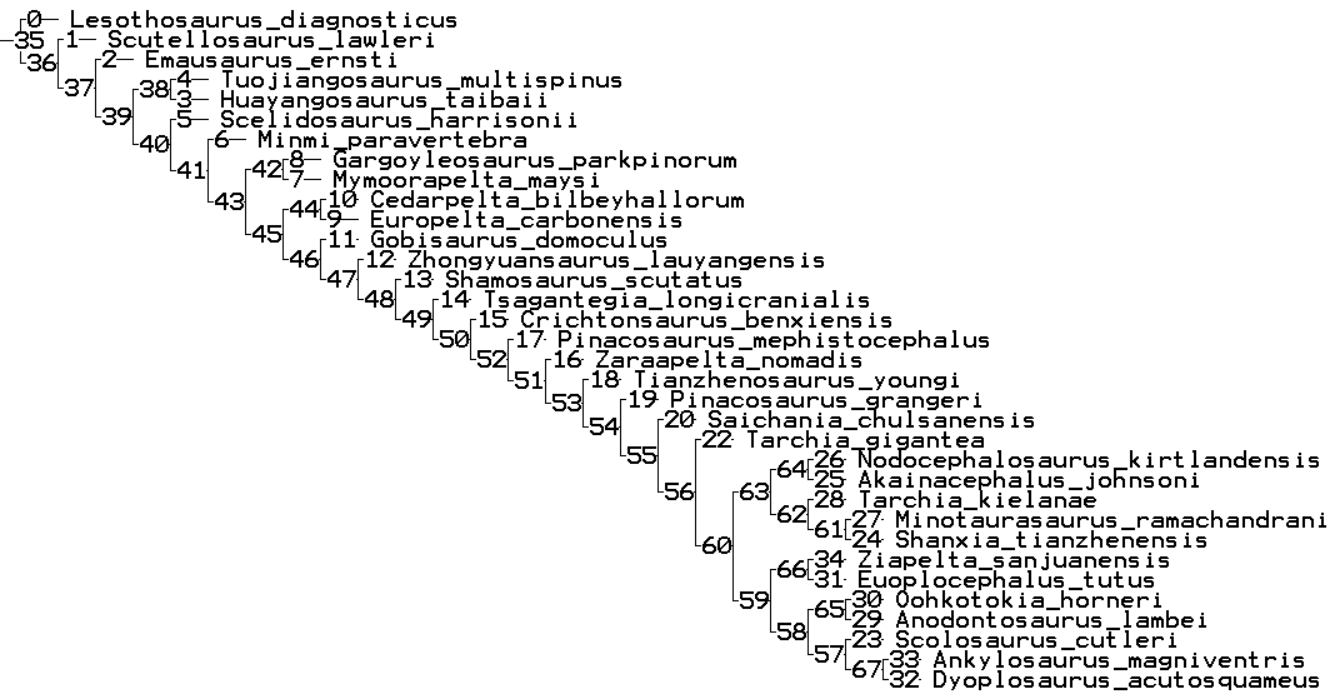


**Figure S7.** Most parsimonious tree from the pruned dataset (*Ahshislepelta* removed), recovering *Akainacephalus johnsoni* as a sister taxon to *Nodocephalosaurus kirtlandensis*, with synapomorphy list.

**Node 37:** No synapomorphies

**Node 38:** 84(1)

**Node 39:** 3(3), 60(1), 72(1), 98(1), 134(1), 145(2), 169(1), 177(1), 179(1), 189(1), 193(1), 196(1), 201(0), 280(1), 281(1), 284(1), 286(0), 288(0)

**Node 40:** 37(1), 97(1), 119(1), 138(1)

**Node 41:** 40(1), 42(1), 82(1), 88(1), 90(1), 92(1), 93(1), 101(1), 120(1), 153(1), 161(1), 162(1), 181(1), 192(1), 199(1), 200(1), 206(1), 219(1), 229(1)

**Node 42:** 1(1), 15(1), 25(1), 30(2), 32(1), 49(2), 61(1), 100(1)

**Node 43:** 51(1), 52(1), 104(1), 115(1), 128(1), 129(1), 152(2), 177(1), 254(1), 260(2)

**Node 44:** 72(1), 98(1), 99(1), 101(2), 122(1), 179(1), 180(1), 196(1), 212(2), 213(1), 230(1), 232(1)

**Node 45:** 6(1), 14(1), 74(1), 144(1), 148(2), 212(1)

**Node 46:** 3(1), 28(1), 44(0), 46(0), 49(1), 50(1), 58(1), 59(1), 73(1), 75(1), 124(1), 132(1), 137(1), 142(1), 158(1), 172(1), 183(1), 188(1), 189(1), 195(1), 197(1), 198(1), 201(2), 220(1), 224(1)

Node 47: 9(1), 12(1), 20(2), 23(1), 31(1), 34(1), 41(1), 48(1), 53(1), 56(1), 61(0), 94(1), 119(2)

**Node 48:** 10(1), 64(1), 111(1)

**Node 49:** 3(0), 134(1)

**Node 50:** 17(1), 21(1), 80(0), 102(1), 103(2), 104(1)

**Node 51:** 2(1), 4(0), 43(1), 44(1), 45(1), 48(3), 49(2), 56(0), 57(1), 63(1), 67(1), 70(1)

**Node 52:** 1(3), 60(1), 104(2)

**Node 53:** No synapomorphies

**Node 54:** 35(1), 54(1), 103(3), 104(3)

**Node 55:** 4(1), 55(1)

**Node 56:** 11(1), 129(1), 202(1), 203(1), 204(1), 228(1)

**Node 57:** 227(1)

**Node 58:** 211(1), 262(1)

**Node 59:** 189(0), 191(0)

**Node 60:** 39(0), 64(1)

**Node 61:** 109(0), 170(1)

**Node 62:** 1(2), 2(2), 67(0), 103(1), 104(1), 111(2), 140(1), 145(2)

**Node 63:** 5(1), 55(0), 64(2), 121(1), 262(0)

**Node 64:** 227(2)

**Node 65:** No synapomorphies

**Node 66:** 70(0), 118(1)

**Node 67:** 132(0), 133(1)

**Node 68:** 4(0), 10(0), 41(0), 46(1), 57(0)

**Node 69:** 250(1)


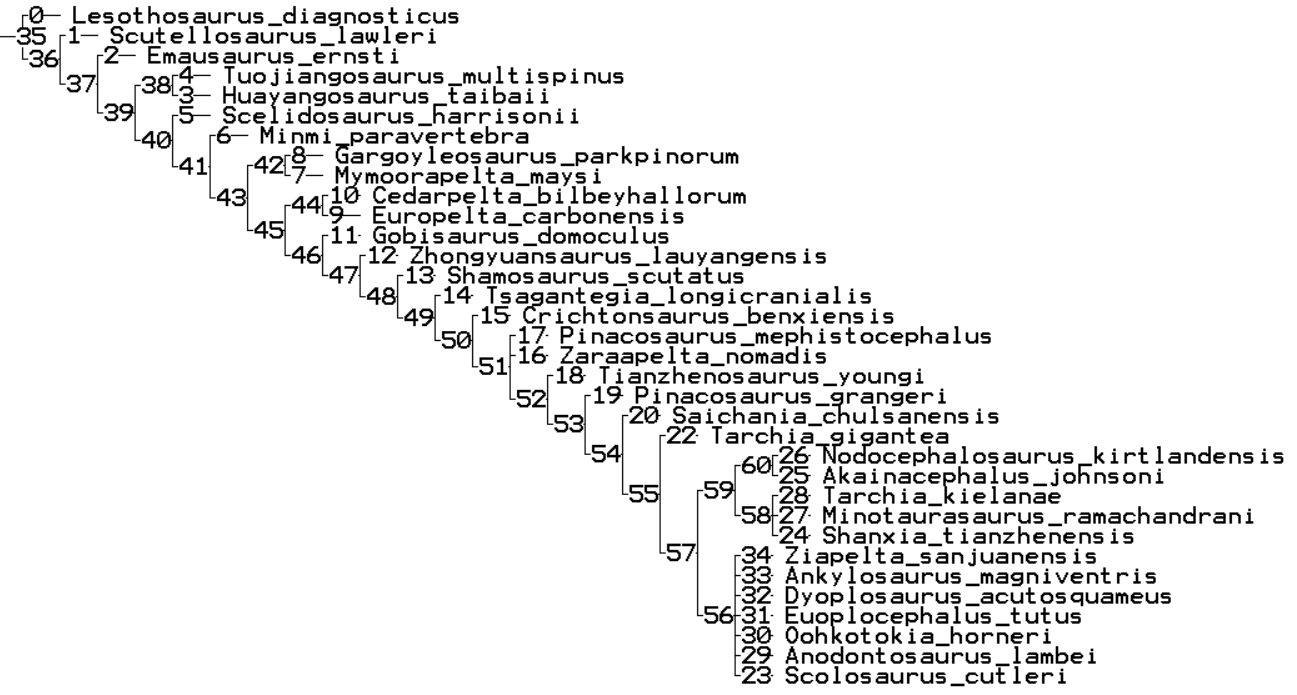


**Figure S8.** Strict Consensus tree from 6 equally most parsimonious phylogenetic trees. The wildcard taxon *Ahshislepelta minor* has been pruned from the dataset.


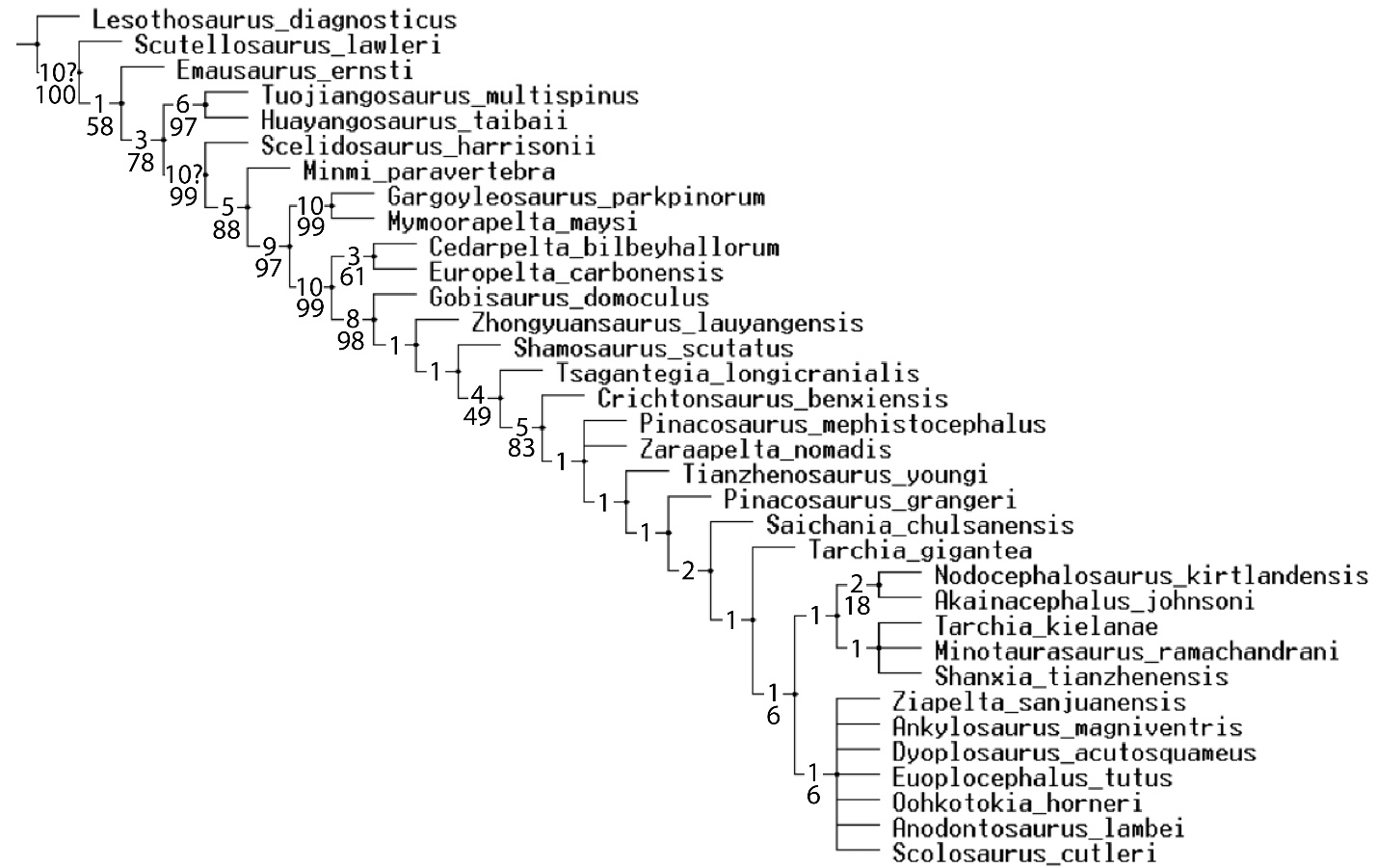


**Figure S9.** Branch support for the pruned phylogenetic analysis, superimposed on the strict consensus of six equally most parsimonious phylogenetic trees. Bremer support values are indicated as the top value on each branch; bootstrap values (10,000 replicates, sampling with replacement) are indicated as the bottom value.


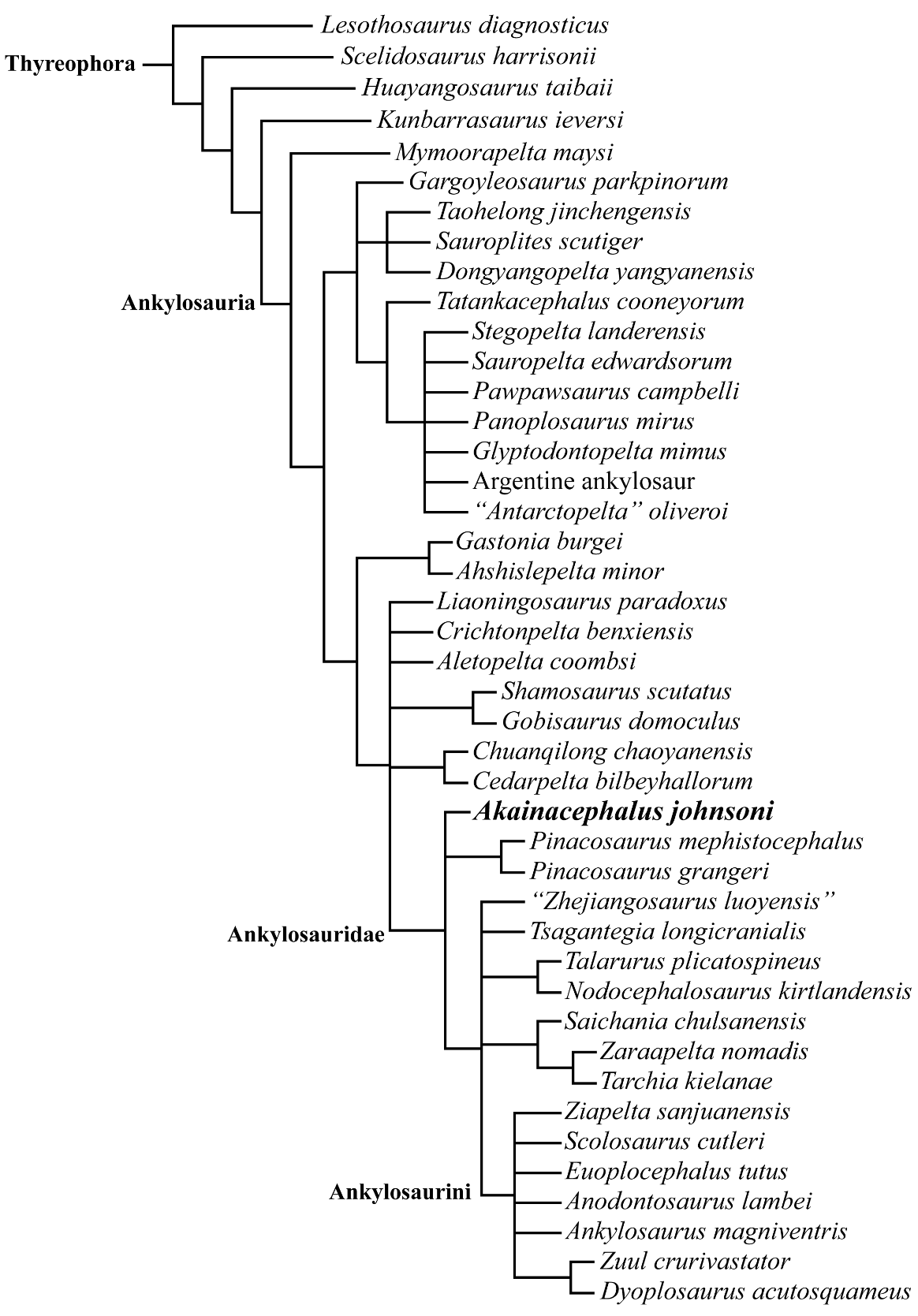


**Figure S10.** 50% Majority rule tree, resolving *Akainacephalus johnsoni* as an ankylosaurid, more closely related to Asian ankylosaurid taxa than Laramidian taxa. Note that the resulting topology does not suggest a close evolutionary relationship between *A. johnsoni* and *Nodocephalosaurus kirtlandensis*.
