# Supplementary material for: A new southern Laramidian ankylosaurid, Akainacephalus johnsoni gen. et sp. nov., from the upper Campanian Kaiparowits Formation of southern Utah, USA
Source: PeerJ. 2018 Jul 19;6:e5016. doi: 10.7717/peerj.5016 (PMC6063217; doi:10.7717/peerj.5016)
Supplement: Supplemental Information 2 [file peerj-06-5016-s002.docx]

**Text S2. Character list used for phylogenetic analysis.** List of character descriptions (Loewen and Kirkland, in prep) and their respective character states used for scoring the new Kaiparowits ankylosaur taxon *Akainacephalus johnsoni*.

1. **Skull, width in dorsal view compared to length:**

(0) – width less than 65% length

(1) – width 70% to 90% length

(2) – width more than 95% to 110% length

(3) – width more than 115% length

**ORDERED**

(Modified from Kirkland 1998:2; Carpenter et al. 1998:1; Sereno 1999:94; Vickaryous et al. 2004:1; Thompson et al. 2011:4)

1. **Skull, maximum dorsoventral height in lateral view compared to skull length from premaxilla to occipital condyle:**

(0) – short, height less than 45% length

(1) – height from 48% to 58% length

(2) – tall, height more than 60% length

**ORDERED**

1. **Skull, width at the center of the orbit in dorsal view compared to width at the posterior position of the squamosals:**

(0) – width of orbits less than 100% width at the squamosal

(1) – width of orbits more than 105% to 125% width at the squamosal

(2) – width of orbits more than 130% to 145% width at the squamosal

(3) – width of orbits more than 150% width at the squamosal

**ORDERED**

(Modified from Kirkland 1998:1; Vickaryous et al. 2004:10; Thompson et al. 2011:5)

1. **Skull, posterior surface, width across paroccipital processes compared to the height from quadrate to the top of the paroccipital process:**
2. – width less than 195% height from quadrate to top of paroccipital process
3. – width greater than 200% height from quadrate to top of paroccipital process
4. **Skull, snout roof in lateral profile rostral to orbits:**

(0) – flat

(1) – domelike

(Kirkland, 1998:3; Carpenter et al. 1998:23; Vickaryous et al. 2004:2; Thompson et al. 2011:14)

1. **Skull, cranial roof in lateral profile between and behind the orbits:**
2. – flat or concave
3. – domelike

(Modified from Vickaryous et al. 2004:3; Thompson et al. 2011:31)

1. **Skull, non-domed cranial roof in lateral profile between and behind the orbits:**
2. – flat
3. – slightly concave
4. – strongly concave

**ORDERED**

1. **Skull roof in dorsal view presence of a distinct posttemporal notch between the postorbital and the squamosal only, regardless of the length of the postorbital and squamosal horns:**
2. – notch absent
3. – notch present
4. **Skull, nuchal shelf:**
5. – does not obscure occiput in dorsal view
6. – obscures occiput in dorsal view

(Kirkland 1999:25; Carpenter et al. 1998:12; Vickaryous et al. 2004:12; Thompson et al. 2011:89)

1. **Skull, external nares orientation:**

(0) – laterally

(1) – anterolaterally

(2) – anteriorly

**ORDERED**

(Modified from Carpenter et al. 1998:10; Thompson et al. 2011:7)

1. **Skull, external nares, visibility in dorsal view:**

(0) – most of the external naris is visible in dorsal view

(1) – almost completely hidden

(Thompson et al. 2011:8)

1. **Skull, external nares, position of anterior border in dorsal view:**

(0) – near the front of the premaxilla

(1) – posteriorly displaced

1. **Skull, presence of the antorbital fenestra:**
2. – present
3. – present but small
4. – absent

**ORDERED**

(Modified from Vickaryous et al. 2004:42; Thompson et al. 2011:1)

1. **Skull, undistorted orbit shape:**
2. – round
3. – sub-rectangular with squared corners
4. **Skull, supratemporal fenestra presence:**
5. – present
6. – absent

(Sereno, 1999:54; Vickaryous et al. 2004:43; Thompson et al. 2011:3)

1. **Skull, expression of laterotemporal fenestra in lateral view:**
2. – completely visible
3. – partially hidden as lateral expansion of the skull has the laterotemporal fenestra facing caudally
4. – completely hidden

**ORDERED**

(Modified from Kirkland 1998:13; Carpenter et al. 1998:6; Vickaryous et al. 2004:4; Thompson et al. 2006:2)

1. **Premaxilla, maximum width of the premaxillary rostrum:**
2. – maximum width of the premaxilla is less than the distance between the caudalmost maxillary teeth
3. – maximum width of the premaxilla is equal to or greater than the distance between the caudalmost maxillary teeth

(Kirkland, 1998:4; Vickaryous et al. 2004:14)

1. **Premaxilla, ventral surface in rostral view:**
2. – ventral surface of premaxilla is flat or convex so that wide premaxillary notch is absent
3. – premaxilla ventral surface is concave so that wide premaxillary notch is present
4. – premaxilla ventral surface is concave so that very narrow premaxillary notch is present

**ORDERED**

(Modified from Vickaryous et al. 2004:15; Thompson et al. 2011:20)

1. **Premaxilla, presence of a deflected cutting edge posterolaterally:**
2. – absent
3. – present
4. **Premaxilla and maxilla, cutting edge of beak:**
5. – contains teeth or the cutting edge is restricted to an extreme rostral position
6. – extend caudally, so that the cutting surface is continuous with maxillary tooth row
7. – extends caudally, lateral to maxillary tooth row so that maxillary teeth are medial to the cutting surface

(Modified from Sereno 1999:101; Kirkland 1998:6; Carpenter et al. 1998:14; Vickaryous et al. 2004:16; Thompson et al. 2011:21)

1. **Premaxilla, maximum anteroposterior length of the premaxillary rostrum:**
2. – premaxillary palate length equal to or greater than premaxillary palate width
3. – premaxillary palate length less than premaxillary palate width

(Modified from Vickaryous et al. 2004:13 Thompson et al. 2011:18)

1. **Premaxilla, flat premaxillary shelf forming roof over extreme anterior end of palate:**
2. – absent
3. – present
4. **Premaxilla, shape of the premaxillary palate:**
5. – sub-triangular to elongated
6. – sub-quadrangular
7. – sub-oval

**ORDERED**

(Modified from Sereno 1999:80; Thompson et al. 2011:19)

1. **Premaxilla, fusion:**
2. – unfused so that midline suture is visible
3. – fused so that midline suture is completely obscured
4. **Premaxilla, overall shape in dorsal view:**
5. – V-shaped
6. – U-shaped
7. – squared or rectangular with flat rostral surface

**ORDERED**

1. **Premaxilla, anterolateral corner forms lateral flange that projects laterally to become the widest point on the premaxilla so that cutting surface on the ventral margin is bifurcated:**
2. – absent
3. – present
4. **Maxillary tooth row orientation relative to each other:**
5. – linear rostrally, diverge caudally
6. – curved into an hourglass-shape, diverge rostrally and caudally, converge midway along the tooth row

(Modified from Vickaryous et al. 2004:16; Thompson et al. 2011:24)

1. **Maxilla, anterolateral corner forms lateral flange as continuation of cutting surface of snout:**
2. – absent
3. – present
4. **Maxilla, anterolateral corner flange, orientation:**
5. – lateral
6. – vertical
7. **Maxilla, tooth row inset medially from lateral surface:**
8. – absent
9. – present, slightly inset
10. – present, strongly inset

**ORDERED**

(Modified from Carpenter et al. 1998: 16; Vickaryous et al. 2004:22; Maidment et al. 2008:5; Thompson et al. 2011:25)

1. **Maxilla, presence of paranasal sinus cavities:**
2. – absent
3. – present

(Vickaryous et al. 2004:26; Thompson et al. 2011:12)

**Nasal and Palate.**

1. **Nasal, length vs. width:**
2. – nasals long, length more than 2 times width
3. – nasals short, length less than 1.5 times width
4. **Nasal, internasal fusion in adults:**

(0) – unfused

(1) – fused

1. **Nasal, sagittal internasal cavity septum:**

(0) – incomplete, does not separate nasal passages

(1) – present complete

(Vickaryous et al. 2004:20; Thompson et al. 2011:10)

1. **Palate, secondary palate complex between tooth rows:**
2. – absent
3. – complex secondary palate formed by palatine and vomers between the tooth rows
4. **Palpebral, presence of a rod-shaped palpebral:**
5. – rod-shaped palpebral present
6. – plate-shaped palpebral, possibly mobile, contacting only the prefrontal
7. – plate-shaped and totally fused into the orbit to become the anterior supraorbital

**ORDERED**

(Modified from Sereno 1999:5; Parish 2005:23; Thompson et al. 2011:27)

1. **Form of palpebral articulation:**
2. – mobile contact with prefrontal
3. – extensive sutural contact with prefrontal, frontal and postorbital, palpebral forms anterodorsal rim of the orbit

(Sereno, 1986, 1999:9; Parish 2005:24; Thompson et al. 2011:28)

1. **Supraorbitals, number of supraorbitals:**
2. – one large supraorbital (the palpebral) and prefrontal present
3. – two supraorbitals and prefrontal present
4. – three supraorbitals and prefrontal present

**ORDERED**

1. **Orbits, angle of orbital axis:**

(0) – laterally oriented, angle of the surface of the orbit subequal to sagittal plane

(1) – anterolaterally oriented

(Parish 2005:11; Thompson et al. 2011:13)

1. **Orbit, presence of a preoccular wall present in anterior wall of internal orbit separating the orbit from the antorbital space:**
2. – absent
3. – present
4. **Orbit, presence of a suborbital lip forming a thin, sharp flange on the lateral edge of the ventral surface of the orbit:**
5. – absent
6. – present
7. **Postorbital, postoccular wall present in caudal wall of internal orbit:**
8. – absent
9. – present

(Vickaryous et al. 2004:41; Thompson et al. 2011:15)

1. **Postorbital, supraorbital postorbital boss:**
2. – absent or minimal
3. – present

(Modified from Vickaryous et al. 2004:5)

1. **Postorbital, supraorbital boss form:**
2. – rounded protuberance
3. – longitudinal ridge or peak

(Modified from Vickaryous et al. 2004:5)

1. **Postorbital, supraorbital boss, overall orientation of the boss in anterior view:**
2. – laterally oriented
3. – dorsolaterally oriented

(Modified from Vickaryous et al. 2004:5)

1. **Postorbital, supraorbital boss, overall orientation of the boss in dorsal view:**
2. – laterally
3. – posterolaterally
4. **Postorbital, supraorbital boss, position of apex of boss compared to the dorsal margin of the orbit:**
5. – positioned dorsal to the dorsal portion of the orbit
6. – positioned ventral to the dorsal portion of the orbit
7. **Squamosal, squamosal boss:**

(0) – absent

(1) – present, rounded protuberance

(2) – present, low or equilateral pyramidal protuberance

(3) – elongated triangle longer than wide

**ORDERED**

(Modified from Vickaryous et al. 2004:6)

1. **Suborbital boss or cornice:**
2. – absent
3. – present, rounded protuberance
4. – present, deltaic protuberance

**ORDERED**

(Modified from Carpenter et al. 1998:5; Vickaryous et al. 2004:7)

1. **Suborbital boss composition:**
2. – formed from jugal only
3. – formed by jugal and quadratojugal
4. **Jugal, medial surface, large medially facing pocket:**
5. – absent
6. – present
7. **Quadratojugal, visible in lateral view:**
8. – visible posterior to jugal
9. – not visible, quadratojugal is medial to jugal
10. **Quadrate, lateral profile:**
11. – bowed, anteriorally convex, caudally concave
12. – linear

(Vickaryous et al. 2004:38; Thompson et al. 2011:33)

1. **Quadrate, inclination of quadrate in lateral view:**
2. – near perpendicular to skull roof 70-90º
3. – anterolaterally from 60º to 40º
4. – nearly horizontal, less than 30º from skull roof

**ORDERED**

(Modified from Lee 1996:10; Kirkland 1998:14; Carpenter et al 1998:20; Parish 2005: 32; Thompson et al. 2011:34)

1. **Quadrate, cross-sectional shape of the anterior surface of the shaft of the quadrate:**
2. – transversely concave
3. – not concave

(Lee 1996:12; Parish 2005:33; Thompson et al. 2011:35)

1. **Quadrate, fusion of the dorsal end of the quadrate to the paroccipital process:**
2. – unfused
3. – fused

(Vickaryous et al. 2004:39; Thompson et al. 2011:41)

1. **Quadrate, exposure of quadrate condyle in lateral view:**
2. – visible
3. – obscured by the suborbital boss

(Vickaryous et al. 2004:40; Thompson et al. 2011:36)

1. **Quadrate, shape of condylar (articular) surface:**
2. – overall condyle is sub-oval
3. – condyles elongated laterally, width more than 3 times that of anteriorposter length
4. **Quadrate, condylar (articular) end, position of the anteroposteroally thickest point in ventral view:**
5. – the medial condyle is larger so that the thickest point is located medially
6. – in the middle of the condylar end
7. **Foramen magnum orientation:**
8. – directly posteriorally
9. – posterioventrally

(Vickaryous et al. 2004:37; Thompson et al. 2011:62)

1. **Foramen magnum, posterior thickening of the dorsal margin of the foramen magnum relative to surrounding bone forming a dorsal shelf or collar above foramen magnum:**
2. – little difference
3. – distinctly thickened

(Modified from Parish 2005:49; Thompson et al. 2011:53)

1. **Paroccipital process, orientation of long axis in posterior view:**
2. – directed ventrolaterally
3. – directed horizontally
4. **Paroccipital process, orientation of long axis in dorsal view:**
5. – directed posterolaterally
6. – directed laterally

(Vickaryous et al. 2004:33; Thompson et al. 2011:51)

1. **Paroccipital process, dorsoventral expansion of distal paroccipital processes compared to the neck:**
2. – expanded to more than 200% the dorsoventral height of the neck
3. – expanded, but less than 150% the dorsoventral height of the neck
4. – not expanded

**ORDERED**

(Modified from Parish 2005:48; Thompson et al. 2011:52)

1. **Basioccipital, form of the ventral surface of basioccipital-basisphenoid:**
2. – transversely convex
3. – ventral surface has a distinct medial depression

(Modified from Parish 2005:51; Thompson et al. 2011:55**)**

1. **Basioccipital, distinct medial longitudinal ridge on ventral surface:**
2. – absent
3. – present

(Modified from Parish 2005:51; Thompson et al. 2011:55**)**

1. **Basioccipital, basioccipital foramen:**
2. – absent
3. – present
4. **Occipital condyle, composition:**
5. – multiple elements are evident by sutures in the occipital condyle
6. – basioccipital is the only contributor to the occipital condyle excluding the suture

(Vickaryous et al. 2004:34; Thompson et al. 2011:54)

1. **Occipital condyle, morphology in occipital view:**
2. – reniform
3. – ovoid/round

(Vickaryous et al. 2004:35)

1. **Occipital condyle, orientation of the neck of the occipital condyle:**
2. – directly caudally
3. – caudoventrally

(Modified from Vickaryous et al. 2004:36; Thompson et al. 2011:61)

1. **Occipital condyle, orientation of the articular surface:**
2. – directly caudally
3. – caudoventrally

1. **Basisphenoid, length between basal tubera and basipterygoids compared to length of basioccipital:**
2. – long, greater than basioccipital length
3. – short, less than basioccipital length

(Vickaryous et al. 2004:31; Thompson et al. 2011:56)

1. **Basisphenoid, basal tubera morphology:**
2. – medially separated rounded rugose stubs
3. – continuous transverse rugose ridge

(Vickaryous et al. 2004:32; Thompson et al. 2011:57)

1. **Basipterygoid, basipterygoid-pterygoid fusion:**
2. – unfused
3. – fused

(Modified from Vickaryous et al. 2004:30; Thompson et al. 2011:44)

1. **Basipterygoid, size of basipterygoid processes:**
2. – long, twice or more as long as wide
3. – short, less than twice as long as wide

(Parish 2005:55; Thompson et al. 2011:58)

1. **Pterygoid, extensive medial contact between pterygoids to form pterygoid shield:**

(0) – absent

(1) – pterygoids joined medially forming a pterygoid shield

(Modified from Parish 2005:40; Thompson et al. 2011:42)

1. **Pterygoid, interpterygoid vacuity:**

(0) – pterygoids separate posteromedially, forming an interpterygoid vacuity

(1) – absent

(Modified from Parish 2005:40; Thompson et al. 2011:42)

1. **Pterygoid, posterior margin of the pterygoid:**
2. – anterior to the ventral margin of the pterygoid process of the quadrate
3. – in transverse alignment with or posterior to the ventral margin of the pterygoid process of the quadrate

(Modified from Sereno, 1999:83; Vickaryous et al. 2004:28)

1. **Pterygoid, pterygoid foramen:**
2. – absent
3. – present

(Hill 2003:21; Thompson et al. 2011:47)

1. **Pterygoid, orientation of the pterygoid flange in anterior view:**
2. – anterolateral flange is obliquely oriented
3. – flange is oriented vertically (parasagittally)
4. – flange is oriented nearly laterally

(Modified from Vickaryous et al. 2004:29; Thompson et al. 2011:43)

1. **Pterygoid, orientation of surface between posterior margin and pterygoid flanges:**

(0) – nearly horizontal, forming caudal secondary palate

(1) – posteroventrally angled

(Modified heavily from Vickaryous et al. 2004:21; Thompson et al. 2011:49)

1. **Predentary, size of predentary ventral process:**

(0) – distinct, prong shaped process

(1) – rudimentary eminence

(Sereno 1986, 1999:66; Parish 2005:72; Thompson et al. 2011:76)

1. **Dentary, depth of the dentary symphysial ramus relative to the maximum depth of the dentary in lateral view:**

(0) – deep, symphysial ramus more than 50% maximum dentary depth

(1) – shallow, less than 45% maximum dentary depth

(Sereno 1986, 1999:17; Parish 2005:64; Thompson et al. 2011:69)

1. **Dentary, shape of dorsal margin of the dentary in lateral view:**
2. – straight
3. – sinuous or convex

(Sereno 1999:4; Parish 2005:65; Thompson et al. 2011:70)

1. **Dentary, shape of ventral margin of the dentary in lateral view excluding the symphasis:**

(0) – straight

(1) – sigmoidal or concave

(Modified from Parish 2005:66; Thompson et al. 2011:71)

1. **Dentary, shape of the alveolar margin in dorsal view:**

(0) – straight

(1) – laterally concave

(2) – laterally convex or sigmoidal

(Modified from Parish 2005:67; Thompson et al. 2011:72)

1. **Dentary, presence of a horizontal shelf lateral to the tooth row:**
2. – present as a rounded protrubance
3. – present, as a distinct ridge, but with no lateral expansion to form a lateral shelf
4. – present, as a distinct ridge with lateral expansion to form a lateral shelf

**ORDERED**

1. **Dentary, size and projection of the dorsal surangular process:**

(0) – small with no dorsal projection

(1) – well developed with a medially positioned dorsal projection

(Parish 2005:71; Thompson et al. 2011:75)

1. **Surangular, lateral ridge on dorsolateral surface of surangular:**
2. – absent
3. – present

(Butler et al., 2010:106)

1. **Surangular, coronoid process:**

(0) – absent

(1) – present

1. **Surangular, coronoid process height:**

(0) – low

(1) – higher than 30% length

1. – super high almost as high as long
2. **Internal mandibular fossa, presence of dorsal roof formed by coronoid process:**

(0) – absent

(1) – present across entire coronoid dorsal surface

1. **External mandibular fenestra:**
2. – present
3. – absent

(Vickaryous et al. 2004:44; Thompson et al. 2011:68)

1. **Premaxilla, premaxillary teeth:**
2. – present
3. – absent

(Kirkland, 1998:20; Carpenter et al 1998:15; Vickaryous et al. 2004:17; Thompson et al. 2011:63)

1. **Maxilla, tooth row extends to rostral end of maxilla:**
2. – present or extends to within one alveolus width to the anterior end of the dentary
3. – absent, diastema at least two times individual alveolus length is present
4. **Dentary, teeth extend nearly to the symphasis or predentary contact:**
5. – present
6. – absent, there is a diastema between the symphasis and the rostralmost tooth
7. **Dentary or maxillary teeth, presence of cingulum:**
8. – absent
9. – present

(Modified from Vickaryous et al. 2004:19; Thompson et al. 2011:64)

1. **Maxillary and dentary teeth, relative size:**
2. – relatively large
3. – relatively small
4. – tiny

**ORDERED**

1. **Cranial sutures on posterior skull roof:**
2. – visible
3. – obliterated

(Modified from Sereno 1986, 1999:63; Hill et al. 2003: 36; Thompson et al. 2011:17)

1. **Cranial sutures on lateral skull:**
2. – visible
3. – obliterated
4. **Cranial ornamentation:**
5. – absent
6. – minimal
7. – extensive with scale impressions

**ORDERED**

1. **Cranial ornamentation, distinct pattern of scale polygons:**
2. – polygons absent
3. – distinct pattern of polygons
4. **Cranial ornamentation, bone remodeling under the scale impressions:**
5. – absent or minimal remodeling
6. – modified to add bone perpendicular to scale but the impression is still flat
7. – extensive remodeling with rounded bulbous scale impressions
8. – extensive remodeling with peaked bulbous scale impressions

**ORDERED**

1. **Cranial ornamentation, form of scale impressions on nasal region of skull roof:**

(0) – absent

(1) – flat

(2) – rounded

(3) – peaked

**ORDERED**

1. **Cranial ornamentation, form of bulbous scale impressions on frontoparietal region of skull roof:**

(0) – flat

(1) – rounded

(2) – peaked

**ORDERED**

1. **Cranial ornamentation, number of scale impressions on skull roof:**
2. – 20 or less
3. – 30 or more
4. **Cranial ornamentation, presence of ornamentation on the external surface of the premaxillae:**
5. – absent, smooth
6. – present as rugose ornamentation

(Vickaryous et al. 2004:62)

1. **Cranial ornamentation, presence of a “beak line” separating nasal armor from premaxillary armo**r**:**

(0) – absent, armor uniform across premaxillary nasal suture

(1) – transverse line separating relative smooth premaxilla from heavily rugose nasal

1. **Cranial ornamentation, nasal ornamentation compared to premaxillary ornamentation:**

(0) – nasal ornamentation similar to that of premaxilla

(1) – nasal ornamentation much more pronounced than premaxillary ornamentation

1. **Cranial ornamentation, nasal region, raised ring of scales surrounding the dorsal and caudal rim of the external naris:**

(0) – absent

(1) – present

1. **Cranial ornamentation, nasal region, presence of a large midline ornamentation between the external nares:**
2. – absent
3. – multiple (more than 6) polygons between the nares
4. – a large trapezoidal mid-nasal scale impression present
5. – a single nasal scale covers most of the internarial region

(Modified from Vickaryous et al. 2004:9; Thompson et al. 2011:81)

1. **Cranial ornamentation, character of armor between naris and orbits, presence of two thin transverse plates between naris and parietal scale when scale impressions are present in the region:**

(0) – more than three scales on each side of midline between naris and parietal plate

(1) – two thin transverse plates dominate each side between naris and parietal plate

1. **Cranial ornamentation, presence of a large midline frontal scale:**

(0) – absent

(1) – present

1. **Cranial ornamentation, presence of a large frontal-parietal scale:**
2. – consists of three or more flat scales
3. – one large scale present

(Modified from Kirkland 1998:27; Carpenter et al. 1998:9; Vickaryous et al. 2004:8)

1. **Cranial ornamentation, distinct pattern of rostrolaterally trending lines radiating from the midline of the caudal region of the parietal:**
2. – absent
3. – distinct radiating lines present
4. **Cranial ornamentation, presence of a distinct circumorbital ring scale complex:**
5. – absent
6. – distinct ring of scales around orbit
7. **Cranial ornamentation, small scale impressions between squamosal horn and quadratojugal horn:**
8. – absent
9. – present

(Modified from Arbour and Currie 2013:171)

1. **Cranial ornamentation, presence of a horn in a depression between the postorbital and squamosal horns:**
2. – absent
3. – present

1. **Cranial ornamentation, nuchal sculpturing:**
2. – absent
3. – present as a rounded thickening at the parietosupraoccipital suture
4. – present, forms horizontal shelf overhanging the supraoccipital

**ORDERED**

(Modified from Vickaryous et al. 2004:11; Thompson et al. 2011:88)

1. **Mandibular ornamentation, presence of ornamentation on lateral surface of mandible:**
2. – absent
3. – present

(Modified from Sereno, 1986, 1999:65; Vickaryous et al. 2004:45; Thompson et al. 2011:91)

1. **Mandibular ornamentation, anterior extent of distinct boss on lateral surface of mandible:**
2. – does not approach anterior end of dentary tooth row
3. – approaches anterior end of tooth row

(Carpenter et al. 1999; Parish 2005:83; Thompson et al. 2011:60)

1. **Mandibular ornamentation, ventral extent of distinct boss on lateral surface of mandible:**

(0) – doesn’t extend below the ventral edge of the angular and dentary

(1) – extends well below the ventral edge of the angular and dentary

1. **Atlas, fusion to axis:**
2. – separate
3. – fused

(Vickaryous et al. 2004:46; Thompson et al. 2011:94)

1. **Atlantal neural arch, fusion to atlas:**
2. – unfused, open
3. – fused in adult

(Modified from Sereno 1999:19; Parish 2005:84; Thompson et al. 2011:97:92)

1. **Atlantal neural arches, median contact between both sides:**
2. – no median contact
3. – median contact two sides fused together into complete arch

(Sereno 1986, 1999:68; Parish 2005:85; Thompson et al. 2011:93)

1. **Cervical vertebrae, anterioposterior length of the centrum compared to dorsoventral centrum height:**
2. – long, length greater than 110% centrum height
3. – short, length less than height

(Modified from Carpenter et al. 1999; Parish 2005:88; Thompson et al. 2011:96)

1. **Cervical vertebrae, mediolateral width compared to anteroposterior centrum length:**
2. – longer than wide
3. – wider than long

(Modified from Kirkland et al. 1998; Parish 2005:87; Thompson et al. 2011:95)

1. **Cervical vertebrae, alignment of vertebral centrum faces of anterior cervical vertebrae:**
2. – anterior and posterior faces are parallel and aligned
3. – anterior face elevated dorsally compared to the posterior face
4. – posterior face elevated dorsally compared to the anterior face

(Vickaryous et al. 2004:47; Thompson et al. 2011:97)

1. **Cervical vertebrae, saggital keel on ventral surface:**
2. – absent
3. – present
4. **Cervical vertebrae, fossa on ventral surface:**
5. – absent
6. – present
7. **Cervical vertebrae, fossa on ventral surface, presence of the keel:**
8. – absent
9. – present
10. **Dorsal vertebrae, ratio of anteroposterior centrum length to posterior centrum height:**
11. – long, length more than 110% centrum height
12. – short, subequal to or shorter than tall

(Parish 2005:89; Thompson et al. 2011:98)

1. **Dorsal vertebrae, longitudinal keel on ventral surface of centra:**
2. –absent
3. – present

(Modified from Parish 2005:90; Thompson et al. 2011:99)

1. **Posterior free dorsal vertebrae, cross-sectional shape of neural canal:**
2. – circular
3. – elliptical, with long axis running dorsoventrally

(Carpenter 1990; Parish 2005:91; Thompson et al. 2011:100)

1. **Posterior free dorsal vertebrae, presence of ossified tendons along neural spine:**
2. – absent
3. – present

(Maidment et al. 2010)

1. **Posterior free dorsal vertebrae, presence of paravertebrae along neural spine:**
2. – absent
3. – present

(Thompson et al. 2011:104)

1. **Dorsal ribs, fusion with centra:**
2. – absent
3. – present

(Vickaryous et al. 2004:48; Thompson et al. 2011:102)

1. **Dorsal ribs, cross-sectional shape of proximal end:**
2. – triangular
3. – L-shaped or T-shaped

(Parish 2005:92; Thompson et al. 2011:101)

1. **Sacrum, presence of a synsacrum of co-ossified dorsal, sacral and caudal vertebrae:**
2. – absent
3. – present

(Vickaryous et al. 2004:61 Parish 2005:94; Thompson et al. 2011:103)

1. **Sacrum, number of fused sacrodorsals in the presacral rod:**
2. – 3 or less sacrodorsals
3. – 4 fused sacrodorsals
4. – 5 or more fused sacrodorsals

**ORDERED**

1. **Sacrum, number of true sacral vertebrae:**
2. – 5 or more
3. – 4
4. – 3

**ORDERED**

(Modified from Sereno 1999:69; Parish 2005:96; Thompson et al. 2011:106)

1. **Sacrum, number of fused vertebrae fused in the sacrum:**
2. – 3 or less
3. – 4 or more fused
4. **Sacrum, forms a ventrally concave arch in lateral view:**
5. – absent
6. – present, slight arch
7. – present, strong arch

**ORDERED**

1. **Sacrum, longitudinal groove in ventral surface of the sacrum:**
2. – absent
3. – present

(Parish 2005:95; Thompson et al. 2011:105)

1. **Proximal caudal (caudal 1) vertebrae, length:**
2. – long, length more than 110% centrum height
3. – short, length from 95% to 60% centrum height
4. – very short length less than 50% centrum height

**ORDERED**

1. **Proximal caudal (caudal 1) vertebrae, alignment of vertebral centrum faces of vertebrae:**

(0) – anterior and posterior faces are parallel and aligned

(1) – anterior face elevated dorsally compared to the posterior face

1. **Anterior caudal vertebrae, neural spine distal mediolateral expansion of the dorsal end:**
2. – not expanded
3. – expanded so that distal mediolateral width is more than 20% dorsoventral height of spine

(Modified from Carpenter 2001; Parish 2005:97; Thompson et al. 2011:107)

1. **Anterior caudal vertebrae, neural spine height:**

(0) – very short length less than 50% centrum height

(1) – short, more than 90% but less than 200% centrum height

(2) – more than 220% centrum height

**ORDERED**

1. **Proximal caudals, length of transverse process compared to neural spine height:**
2. – less than 80% neural spine length
3. – sub-equal
4. – approximately twice the length

**ORDERED**

(Sereno 1999:70; Parish 2005:99; Thompson et al. 2011:109)

1. **Caudal vertebrae, transverse process, orientation in dorsal view:**
2. – anteriorly projecting
3. – caudally projecting
4. – laterally projecting

(Carpenter 2001; Parish 2005:98; Thompson et al. 2011:108)

1. **Caudal vertebrae, transverse process, orientation in anterior view:**
2. – laterally projecting
3. – ventrally projecting
4. – dorsally projecting

(Modified after Maidment et al. 2008:30)

1. **Caudal vertebrae, transverse process, curvature in anterior view:**
2. – straight
3. – dorsally concave
4. – ventrally concave
5. **Caudal series, persistence of transverse processes down the length of the caudal series:**
6. – not present beyond the mid-length of the series
7. – present beyond the mid-length of the series

(Parish 2005:100; Thompson et al. 2011:110)

1. **Distal caudal postzygapophysis shape:**
2. – short with a sub-triangular end, wedge-shaped
3. – long with a rounded end, tongue-shaped

(Sereno 1999:110; Parish 2005:102; Thompson et al. 2011:112)

1. **Distal caudals, extent of pre- and postzygapophyses over their adjacent centra to form a “handle”:**
2. – extend over less than 45% the length of the adjacent centrum
3. – extend over more than 45% the length of the adjacent centrum

(Modified from Sereno 1999:109; Parish 2005:103; Thompson et al. 2011:113)

1. **Distal caudals, fusion of distal caudals:**
2. – absent
3. – present between individual caudals
4. – present more than 5 distal caudals fused

**ORDERED**

1. **Proximal caudal chevrons, fusion to caudal centra:**
2. – fusion absent, articulated
3. – fused

(Modified from Parish 2005:101; Thompson et al. 2011:111)

1. **Proximal caudal chevrons, expanded at distal tips:**
2. – absent
3. – present
4. **Distal caudal chevrons, shape:**
5. – rod shaped
6. – inverted T-shaped

(Sereno 1986, 1999:71; Parish 2005:104; Thompson et al. 2011:114)

1. **Distal caudal chevrons, fusion:**
2. – absent
3. – present
4. **Distal tail, presence of ossified tendons in distal region of tail:**
5. – absent
6. – present

(Sereno 1999:97; Parish 2005:105; Thompson et al. 2011:115)

**Shoulder Girdle.**

1. **Scapula, acrominon process, development of raised bone perpendicular to the blade of scapula to form tab:**
2. – low ridge
3. – “swolen” process
4. – distinct raised flange

**ORDERED**

(Modified from Vickaryous et al. 2004:52; Thompson et al. 2011:123)

1. **Scapula, acrominon process, form of distinct raised flange:**
2. – knob-like rounded flange
3. – blade-like tab or flange
4. **Scapula, acromion orientation in cross-sectional view of scapular shaft:**
5. – perpendicular to lateral surface of the scapula
6. – refolded laterally to almost parallell the scapular surface
7. **Scapula, position of the base of the acromion process of scapula:**
8. – positioned on the dorsal margin of the scapula
9. – distinct space between the dorsal margin of the scapula and the acromion base
10. – wide space present between the dorsal margin of the scapula and the acromion base, with acromion clearly directed towards the glenoid

**ORDERED**

(Modified from Kirkland 1998:32; Parish 2005:115; Thompson et al. 2011:124)

1. **Scapula, dorsal process of scapula distinct from scapular blade near suture with coracoid:**
2. – dorsal expansion from dorsal edge of scapular blade equal or less than 75% of the minimum dorsoventral dimension of the scapular blade
3. – dorsal expansion from dorsal edge of scapular blade more than 80% of the minimum dorsoventral dimension of the scapular blade but less than 125%
4. – dorsal expansion from dorsal edge of scapular blade more than 150% of the minimum dorsoventral dimension of the scapular blade
5. **Scapula, ventral process of scapula at the caudoventral margin of glenoid near suture with coracoid:**
6. – absent
7. – present

(Modified from Parish 2005:113; Thompson et al. 2011:122)

1. **Scapula, orientation of glenoid:**
2. – ventrolateral
3. – ventral

(Sereno 1999:87; Parish 2005:112; Thompson et al. 2011:121)

1. **Scapula, overall shape of scapular blade in lateral view:**
2. – relatively straight or concave dorsal surface
3. – dorsally convex
4. **Scapula, dorsoventral expansion of distal end of scapula shaft:**
5. – distally expanded to more than 150% the minimum dorsoventral dimension of the scapular blade
6. – absent

(Modified from Sereno 1986, 1999:20; Parish 2005:117: Thompson et al. 2011:126)

1. **Scapula, extent of dorsoventral expansion of distal end of scapula along the long axis of the shaft:**
2. – whole scapula is expanded to form paddle shape
3. – expanded only along the distal 33% of shaft
4. **Scapula and coracoid, fusion:**
5. – articulated
6. – fused

(Parish 2005:110; Thompson et al. 2011:120)

1. **Scapula, presence of scapulocoracoid buttress:**
2. – absent
3. – present

(Parish 2005:116; Thompson et al. 2011:125)

1. **Coracoid, length:**
2. – axis perpendicular to scapular suture is shorter than axis parallel to scapular suture
3. – subequal to axis perpendicular to scapular suture is 105% to 110% longer than axis parallel to scapular suture
4. – axis perpendicular to scapular suture is 120% of axis parallel to scapular suture but is much less than 70% scapula length
5. – axis perpendicular to scapular suture is more than 120% of axis parallel to scapular suture and about 80% scapula length
6. – axis perpendicular to scapular suture is more than 120% of axis parallel to scapular suture and almost as long as the scapula itself within 90% scapula length

**ORDERED**

(Modified from Parish 2005:106; Thompson et al. 2011:116)

1. **Coracoid, shape of dorsal border of coracoid in profile:**
2. – rounded, convex
3. – pointy
4. – straight
5. **Coracoid, shape of ventral border of coracoid in lateral view:**
6. – rounded, convenx
7. – rounded, concave
8. – straight

(Modified from Vickaryous et al. 2004:53; Thompson et al. 2011:117)

1. **Coracoid, presence of anteroventral process:**
2. – absent
3. – short process present with distinct notch between glenoid and process
4. – long process present with distinct notch between glenoid and process

**ORDERED**

(Parish 2005:108; Thompson et al. 2011:118)

1. **Sternal, fusion of bilateral sternal elements:**
2. – not fused
3. – fused

(Sereno 1986, 1999: 112; Vickaryous et al. 2004:60; Thompson et al. 2011:127)

1. **Forelimb, robusticity:**
2. – slender
3. – robust
4. **Forelimb, length of distal limb elements short with respect to the humerus:**
5. – long lower limb, radius or ulna more than 70% length of humerus
6. – shortened lower limbs
7. **Humerus, separation of humeral head and deltopectoral crest in anterior view:**
8. – continuous
9. – separated by a distinct notch or peak

(Parish 2005:119; Thompson et al. 2011:128)

1. **Humerus, separation of humeral head and medial tubercle in anterior view:**
2. – continuous
3. – separated by a distinct notch or peak

(Parish 2005:119; Thompson et al. 2011:129)

1. **Humerus, length of deltopectoral crest relative to overall length of the humerus:**
2. – short less than 50%
3. – long approximately equal to or greater than 50%

(Kirkland, 1998:35; Vickaryous et al. 2004:54; Thompson et al. 2011:130)

1. **Humerus, orientation of deltopectoral crest projection:**
2. – lateral
3. – anterolateral

(Sereno 1999:113; Parish 2005:121; Thompson et al. 2011:131)

1. **Humerus, shape of the radial (medial) condyle of distal humerus in distal view (or the proximal end of radius):**
2. – non-circular
3. – circular

(Coombs, 1978; Parish 2005:122; Thompson et al. 2011:132)

1. **Ulna, length of olecranon process (along the articular surface) to total ulnar length:**
2. – short olecranon
3. – long
4. **Metacarpals, ratio of the length of metacarpal V to metacarpal III:**
5. – middle of hand long, MC V shorter than 50% length of MC III
6. – middle of hand short, MC V longer than 55% length of MC III

(Sereno 1999:6; Parish 2005:123; Thompson et al. 2011:133)

1. **Manus, shape of manual unguals:**
2. – claw shaped
3. – hoof shaped

(Sereno 1999:7; Parish 2005:125; Thompson et al. 2011:135)

1. **Ilium, length of the preacetabular process of ilium compared to the postacetabular process (measured from the pubic peduncle at the rostral end of the acetabulum):**
2. – preacetabular ilium shorter than the rest of the ilium ≤ 50% ilium length
3. – preacetabular ilium longer than the rest of the ilium > 50 % ilium length

(Modified from Sereno, 1999:21; Parish 2005:126; Thompson et al. 2011:136)

1. **Ilium, lateral expansion:**
2. – absent
3. – present)

(Modified from Maidment et al. 2018:52.53)

1. **Ilium, angle of lateral deflection of the preacetabular process of the ilium in dorsal view:**
2. – 0º to 20º
3. – more than 30º

(Sereno 1986, 1999:21; Parish 2005:127; Thompson et al. 2011:137)

1. **Ilium, orientation of the preacetabular portion of the ilium:**
2. – near vertical
3. – curled over laterally

(Kirkland 1998:45; Thompson et al. 2011:138)

1. **Ilium, form of the preacetabular portion of the ilium:**
2. – straight process
3. – pronounced ventral curvature

(Parish 2005:129; Thompson et al. 2011:139)

1. **Ilium, form of supraacetabular shelf:**
2. – absent, or oriented vertically
3. – forms a horizontal shelf dorsal to the acetabulum
4. – partially encircles the acetabulum laterally obscures it (supraacetabular flange)

**ORDERED**

(Modified from Kirkland, 1998:45; Vickaryous et al. 2004:55; Thompson et al. 2011:138 & 140)

1. **Ilium, closeure of acetabulum:**
2. – open
3. – closed

(Sereno, 1986; 1999:74; Vickaryous et al. 2004:56; Thompson et al. 2011:141)

1. **Ilium, postacetabular ilium length, relative to diameter of acetabulum:**
2. – postacetabular blade longer than acetabulum
3. – postacetabular blade shorter than acetabulum

(Modified from Sereno, 1999:114; Parish 2005:132; Thompson et al. 2011:142)

1. **Pubis, contribution to lateral rim acetabulum:**
2. – 20% or more
3. – virtually excluded

(Sereno, 1999:117; Vickaryous et al. 2004:59; Thompson et al. 2011:146)

1. **Pubis, overall size:**
2. – large
3. – reduced

(Kirkland 1998:46; Parish 2005:133; Thompson et al. 2011:143)

1. **Pubis, robusticity of the body of the pubis:**
2. – gracile
3. – massive and robust

(Modified from Carpenter 2001; Parish 2005:135; Thompson et al. 2011:145)

1. **Pubis, rotation of the body of the pubis:**
2. – unrotated
3. – dorsolaterally rotated

(Modified from Carpenter 2001; Parish 2005:135; Thompson et al. 2011:145)

1. **Pubis, preacetabular pubic process:**
2. – 3 times longer than the body of the pubis
3. – present longer by 1.2 to 1.8 times the length of the body of the pubis
4. – absent or shorter than body of pubis (not the postpubic process)

**ORDERED**

1. **Pubis, preacetabular pubic process, deflection from sagittal plane:**
2. – sagittally oriented
3. – lateral deflection

(Modified from Vickaryous et al. 2004:56; Parish 2005:134; Thompson et al. 2011:144)

1. **Pubis, preacetabular pubic process, dorsal deflection:**
2. – anteriorally oriented
3. – dorsally oriented

(Modified from Vickaryous et al. 2004:56; Parish 2005:134; Thompson et al. 2011:144)

1. **Pubis, preacetabular pubic process integration into acetabulum:**
2. – free
3. – integrated into acetabulum

(Modified from Vickaryous et al. 2004:56; Parish 2005:134; Thompson et al. 2011:144)

1. **Pubis, opistopubic posterior process:**
2. – long bladelike
3. – long rodlike
4. – short reduced

**ORDERED**

1. **Pubis, post pubic process distal expansion:**
2. – present
3. – absent
4. **Ischium, shaft of ischium:**
5. – little to no curvature
6. – pronounced curvature

(Kirkland, 1998:37; Vickaryous et al. 2004:57; Thompson et al. 2011:147)

1. **Ischium, shape of the dorsal margin of ischium:**
2. – straight
3. – concave
4. – convex

(Modified from Sereno 1999:115; Parish 2005:137; Thompson et al. 2011:148)

1. **Ischium, dorsal surface presence of distinct triangular process:**
2. – absent
3. – present as insipient triangular process
4. – present as distinct triangular process

**ORDERED**

1. **Ischium, distal expansion:**
2. – distal end is distally expanded or blunt
3. – absent, distal end is tapered
4. **Femur, separation of femoral head from greater trochanter:**
5. – continuous
6. – separated by a distinct notch or change in slope

(Parish 2005:139; Thompson et al. 2011:150)

1. **Femur, angle between long axis of femoral head and long axis of shaft:**
2. – less than 100º
3. – 100º to 120º
4. – greater than 120º

(Modified from Parish 2005:138; Thompson et al. 2011:149)

1. **Femur, differentiation of the anterior trochanter of the femur:**
2. – separated from femoral shaft by a deep groove laterally and dorsally
3. – fused to femoral shaft

(Kirkland 1998:36; Parish 2005:140; Thompson et al. 2011:151)

1. **Femur, oblique ridge on lateral femoral shaft, distal to anterior trochanter:**
2. – absent
3. – present

(Parish 2005:141; Thompson et al. 2011:152)

1. **Femur, form of the fourth trochanter:**
2. – pendant
3. – ridge-like

(Sereno 1999:24; Parish 2005:142; Thompson et al. 2011:153)

1. **Femur, location of the fourth trochanter on the femoral shaft:**
2. – proximal to half-way down the femoral shaft
3. – distal, well over half-way down the femoral shaft

(Parish 2005:143; Thompson et al. 2011:154)

1. **Femur, relation of lower limb, (tibua or fibula) to femoral length:**
2. – extremely long, lower limb greater than 105% femoral length
3. – relatively long, lower limb from 95% to 75% femoral length
4. – short, lower limb less than 70% femoral length

**ORDERED**

1. **Tibia, maximum distal width of the tibia, compared to the maximum proximal width:**
2. – narrower or subequal
3. – wider

(Sereno 1999:188; Parish 2005:144; Thompson et al. 2011:155)

1. **Tibia, 70 degree twist between distal and proximal ends:**
2. – absent
3. – present
4. **Tibia, contact between tibia and astragalus:**
5. – articulated
6. – fused, with suture obliterated

(Parish 2005:145; Thompson et al. 2011:156)

1. **Contact between fibula and calcanium:**

(0) – articulated

(1) – fused, with suture obliterated

1. **Astragalas, contact between astragalas and calcanium:**

(0) – articulated

(1) – fused, with suture obliterated

1. **Pes, number of digits with claws:**
2. – 4
3. – 3

(Currie et al. 2011)

1. **Pes, shape of pedal unguals:**
2. – claw shaped
3. – hoof shaped

(Sereno 1999:7; Parish 2005:125; Thompson et al. 2011:135)

1. **Dermal armor, presence of a solid based large armor element with a flat plate for a base and a thin spine emanating from the center (the “splate” of Blows 1987):**

(0) – absent

(1) – present

(Modified from Parish 2005:158; Thompson et al. 2011:169)

1. **Dermal armor, marginal ornamentation on dorsal scutes rim or ridges around the periphery of the osteoderm:**
2. – absent, or smooth
3. – present, rim around plate
4. **Dermal armor, surface texture:**
5. – absent, or smooth
6. – mildly to moderately rugose
7. – extremely rugose

(Burns 2008)

1. **Dermal armor, pitting:**
2. – absent
3. – present, sparse
4. – present, extensive

(Burns 2008; Sereno (1999: 78)

1. **Cervical armor, fusion of osteoderms on dorsal surface of neck region into neck bands or “rings”:**
2. – unfused
3. – sutured together, sometimes to a quarter-ring but not into a half-ring
4. – present, fused into a half-ring

**ORDERED**

(Modified from Kirkland, 1998:38; Vickaryous et al. 2004:49; Thompson et al. 2011:163)

1. **Cervical armor, number of fused cervical armor bands even if one is moved back onto the shoulder:**
2. – 3 or more
3. – 2

(Modified from Thompson et al. 2011:162)

1. **Anteriormost cervical armor band, presence of raised perpendicular ornamentation on medialmost plate (shape in transverse cross-section of medialmost plate (plate 1)):**
2. – present, large keel or spike
3. – present, low keel
4. – low raised bump or rounded swelling

**ORDERED**

1. **Anteriormost cervical armor band, presence of raised perpendicular ornamentation on plate lateral to the medialmost plate (shape in transverse cross-section of plate lateral to medialmost plate (plate 2)):**
2. – present, large keel or spike
3. – present, low keel
4. – low raised bump or rounded swelling

**ORDERED**

1. **Anteriormost cervical armor band, presence of raised perpendicular ornamentation on plate two positions lateral to the medialmost plate (shape in transverse cross-section of plate two positions away from medialmost plate (plate3)):**
2. – present, large keel or spine or spike
3. – present, low keel or raised bump
4. **Anteriormost cervical armor band, presence of raised perpendicular ornamentation on plate three positions lateral to the medialmost plate (shape in transverse cross-section of plate two positions away from medialmost plate (plate4)):**
5. – plate 4 is absent
6. – present, large keel or spine
7. **Cervical armor, osteoderms capping anteriormost cervical armor ring abut each other:**
8. – absent
9. – present

(Modified from Arbour and Currie 2013:172)

1. **Second cervical armor band, presence of raised perpendicular ornamentation on medialmost plate (shape in transverse cross-section of medialmost plate (plate 1)):**
2. – present, large keel or spike
3. – present, low keel
4. – low raised bump or rounded swelling

**ORDERED**

1. **Second cervical armor band, presence of raised perpendicular ornamentation on plate lateral to the medialmost plate (shape in transverse cross-section of plate lateral to medialmost plate (plate 2)):**
2. – present, large keel or spike
3. – present, low keel
4. – low raised bump or rounded swelling

**ORDERED**

1. **Second cervical armor band, presence of raised perpendicular ornamentation on plate two positions lateral to the medialmost plate (shape in transverse cross-section of plate two positions away from medialmost plate (plate3)):**
2. – present, large keel or spike
3. – present, low keel
4. – low raised bump or rounded swelling

**ORDERED**

1. **Second cervical armor band, presence of raised perpendicular ornamentation on plate three positions lateral to the medialmost plate (shape in transverse cross-section of plate two positions away from medialmost plate (plate4)):**
2. – plate 4 is absent
3. – present, large keel or spine
4. **Cervical armor, osteoderms on second cervical armor ring abut each other:**
5. – absent
6. – present

(Modified from Arbour and Currie 2013:172)

1. **Third cervical armor band, presence of raised perpendicular ornamentation on medial most plate (shape in transverse cross-section of medial most plate (plate 1)):**
2. – present, large keel or spike
3. – present, low keel
4. **Third cervical armor band, presence of raised perpendicular ornamentation on plate lateral to the medial most plate (shape in transverse cross-section of plate lateral to medial most plate (plate 2)):**
5. – present, large keel or spike
6. – present, low keel
7. **Third cervical armor band, presence of raised perpendicular ornamentation on plate two positions lateral to the medialmost plate (shape in transverse cross-section of plate two positions away from medialmost plate (plate3)):**
8. – present, large keel or spike
9. – present, low keel
10. **Third cervical armor band, presence of raised perpendicular ornamentation on plate three positions lateral to the medialmost plate (shape in transverse cross-section of plate two positions away from medialmost plate (plate4)):**
11. – plate 4 is absent
12. – present, large keel or spine
13. **Cervical armor, osteoderms on third cervical armor ring abut each other:**
14. – absent
15. – present

(Modified from Arbour and Currie 2013:172)

1. **Third cervical armor band, moved onto shoulder:**
2. – absent
3. – present
4. **Cervical armor, presence of true cervical spines on the cervical armor bands:**
5. – absent
6. – present
7. **Cervical armor, cervical spines, bifurcation:**
8. – absent
9. – present
10. **Cervical armor, orientation of lateral spines:**
11. – laterally or caudolaterally
12. – anterolaterally
13. **Cervical armor, small secondary ossicles fused to cervical armor bands between primary cervical plates 1-3:**
14. – absent
15. – present

(Modified from Arbour and Currie 2013:173)

1. **Thoracic armor, form of base:**
2. – thin and/or hollow
3. – solid or with only small excavation

(Modified after Kirkland, 1998:41)

1. **Thoracic armor, lateral parascapular shoulder spines:**
2. – absent
3. – present without base
4. – present with broad flattened base
5. **Thoracic armor, presence of bifurcated lateral shoulder spines:**
6. – absent
7. – present

(Carpenter; 1990)

1. **Thoracic armor, lateral shoulder spines, presence of a posterior groove:**
2. – conical with a sub-round cross-section
3. – posterior groove

(Modified from Thompson et al. 2011:165)

1. **Thoracic armor, upright dorsal spines:**
2. – absent
3. – present

(Kirkland, 1998:40)

1. **Ventral armor, sheet of armor on the belly:**
2. – absent
3. – present
4. **Sacral spikes on a base, in animals with multiple parasagittal rows of armor:**
5. – absent
6. – present

1. **Sacral spikes on a base, in animals with multiple parasagittal rows of armor length:**
2. – absent or short
3. – longer than wide
4. **Sacral armor, spacing of true sacral osteoderms excluding skin impressions:**
5. – adjacent
6. – abutting each other with true osteoderms abutting

(Modified from Arbour et al. 2011)

1. **Sacral armor, true sacral shield of osteoderm fusion:**
2. – unfused
3. – patches of multiple osteoderms fused but not the complete shield
4. – remodeled into true, fused continuous sacral shield

**ORDERED**

(Modified from Kirkland 1998:42; Parish 2005:155; Thompson et al. 2011:166)

1. **Sacral armor, form of ossicles in sacral armor:**
2. – irregular ossicles
3. – sub-hexagonal ossicles of similar sizes

(Parish 2005:156; Thompson et al. 2011:167)

1. **Lateral triangular caudal armor with hollow bases on tail excluding the distal region:**
2. – absent
3. – present

(Kirkland, 1998:43)

1. **Lateral armor, presence and persistence of large lateral caudal plates:**
2. – present and extend well down the tail
3. – present but only proximal most two or three are large while the rest are small
4. **Lateral caudal plate, spacing:**

(0) – closely spaced

(1) – widely spaced

1. **Lateral caudal plate, symmetry:**

(0) – asymmetrical, recurved

(1) – symmetrical

1. **Distal tail club, presence:**

(0) – absent

(1) – present

(Sereno, 1986; 1999:98; Kirkland, 1998:44; Vickaryous et al. 2004:51; Thompson et al. 2011:170, 88)

1. **Distal tail club, symmetry of lateral plates in dorsal view:**

(0) – semicircular

(1) – triangular

(Arbour and Currie 2013:175)

1. **Distal tail club, proportions of lateral plates in dorsal view:**

(0) – anteriorposterior length longer than 120% mediolateral width

(1) – subequal

(2) – wider than long

**ORDERED**

(Modified from Arbour and Currie 2013:176)

1. **Distal tail club, proportions of caudal plate in dorsal view:**

(0) – longer than wide or subequal

(1) – wider than long

1. **Dentary, shape of rostroventral margin of the dentary, development of a pronounced chin:**
2. – absent
3. – present
4. **Dentary, posterior tooth row visible in lateral view:**

(0) – visible in lateral view

(1) – teeth obscured by thin lateral lamina

(Modified from Maidment et al. 2008:14)

1. **Dentary, orientation of alveoli:**

(0) – alveoli face dorsally

(1) – alveoli face dorsomedially

(Maidment et al. 2008:15)

1. **Quadrate, fossa present on pterygoid flange:**
2. – absent
3. – present

(Sereno, 1986, 1999:29; Maidment et al. 2008:8)

1. **Posterior cervical vertebrae, elongation of the postzygapophyses:**
2. – absent
3. – elongated to project caudal to the centrum face

(Maidment et al. 2008:22)

1. **Posterior free dorsal vertebrae, expansion of pedicoel in anterior view:**
2. – unexpanded
3. – expanded

(Sereno, 1986, 1999:31)

1. **Posterior free dorsal vertebrae, expansion of pedicoel between neural canal and transverse processes compared to the dorsoventral height of the neural canal in anterior view:**
2. – area between neural canal and transverse processes is shorter than neural canal
3. – area between neural canal and transverse processes is at least as tall as the neural canal
4. **Posterior free dorsal vertebrae, neural spine distal mediolateral expansion of the dorsal end:**
5. – absent
6. – present
7. **Anterior midcaudal (caudal 5-7) vertebrae, alignment of vertebral centrum faces of anterior cervical vertebrae:**

(0) – anterior and posterior faces are parallel and aligned

(1) – anterior face elevated dorsally compared to the posterior face

1. **Caudal vertebrae, transverse process, presence of dorsal flange in anterior view:**
2. – absent
3. – present insipient
4. – present pronounced

**ORDERED**

1. **Scapula, orientation of overall acromium process in lateral view:**
2. – subparallel to scapular blade
3. – perpendicular to scapular blade covering dorsal process and continuing to the glenoid
4. **Scapula, dorsal process, anterioposterior extent along dorsal surface of scapula:**
5. – extends less than the length of the coracoid
6. – greater to or equal to the length of the coracoid
7. **Scapula, dorsal process, forms peak away from the suture with the coracoid:**
8. – absent dorsal surface of dorsal process confluent with the dorsal surface of the coracoid
9. – present, dorsal process forms a peak distal to the coracoid suture
10. **Scapula, change in angle between scapular blade and dorsal coracoid suture:**
11. – sweeping curve
12. – 70º to 90º forming “step”
13. **Ilium, supraacetabular shelf form:**
14. – straight lateral edge
15. – semicircular in dorsal view to form semicircular flange

(Modified from Maidment et al. 2008:54)

1. **Ilium, semicircular supraacetabular flange orientation:**
2. – flange projects laterally
3. – flange folded over

(Modified from Maidment et al. 2008:55)

1. **Ilium, postacetabular ilium form of caudal end:**
2. – tapered
3. – blunt

(Modified from Maidment et al. 2008:57)

1. **Ilium, ventromedial flange backing the acetabulum:**
2. – absent
3. – present

(Modified from Maidment et al. 2008:58)

1. **Dermal armor, dominated by a single pair of parasagittal rows of armor:**
2. – present, a single row on each side of the midline make up the majority of armor
3. – multiple rows of armor on each side of the body
4. **Thorassic armor, form of single row of parasagittal armor:**
5. – spikes
6. – plates
7. **Thorassic armor, form of single row of parasagittal plates:**
8. – taller than long and pointed
9. – longer than tall to subequal with rounded tops
10. **Sacral armor, form of single row of parasagittal armor:**
11. – spikes
12. – plates
13. **Caudal armor, form of single row of parasagittal armor, excluding the distal caudal portion:**
14. – spikes
15. – plates
16. **Distal caudal tail spikes, presence:**

(0) – absent

(1) – present
